# Sputum Proteome Signatures of Mechanically Ventilated Intensive Care Unit Patients Distinguish Samples with or without Anti-pneumococcal Activity

**DOI:** 10.1128/mSystems.00702-20

**Published:** 2021-03-02

**Authors:** Jolien Seinen, Rudolf Engelke, Mohammed R. Abdullah, Franziska Voß, Stephan Michalik, Vishnu M. Dhople, Willem Dieperink, Anne Marie G. A. de Smet, Uwe Völker, Jan Maarten van Dijl, Frank Schmidt, Sven Hammerschmidt

**Affiliations:** a Department of Medical Microbiology, University of Groningen, University Medical Center Groningen, Groningen, The Netherlands; b Department of Molecular Genetics and Infection Biology, Interfaculty Institute for Genetics and Functional Genomics, Center for Functional Genomics of Microbes, University of Greifswald, Greifswald, Germany; c Proteomics Core, Weill Cornell Medicine-Qatar, Education City, Doha, Qatar; d Department of Functional Genomics, Interfaculty Institute for Genetics and Functional Genomics, Center for Functional Genomics of Microbes, University Medicine Greifswald, Greifswald, Germany; e Department of Critical Care, University of Groningen, University Medical Center Groningen, Groningen, The Netherlands; Princeton University

**Keywords:** *Streptococcus pneumoniae*, mechanical ventilation, sputum, antimicrobial peptides, immunoproteomics, proteomics

## Abstract

Mechanically ventilated patients are at risk of contracting pneumonia. Therefore, these patients often receive prophylactic systemic antimicrobial therapy. Intriguingly however, a previous study showed that antimicrobial activity in bronchoalveolar aspirates (here referred to as “sputa”) from ventilated patients was only partially explained by antibiotic therapy. Here we report that sputa from these patients presented distinct proteome signatures depending on the presence or absence of antimicrobial activity. Moreover, we show that the same distinction applied to antibodies against Streptococcus pneumoniae, which is a major causative agent of pneumonia. Specifically, the investigated sputa that inhibited growth of S. pneumoniae, while containing subinhibitory levels of the antibiotic cefotaxime, presented elevated levels of proteins implicated in innate immune defenses, including complement and apolipoprotein-associated proteins. In contrast, S. pneumoniae-inhibiting sputa with relatively high cefotaxime concentrations or noninhibiting sputa contained higher levels of proteins involved in inflammatory responses, such as neutrophil elastase-associated proteins. In an immunoproteomics analysis, 18 out of 55 S. pneumoniae antigens tested showed significantly increased levels of IgGs in inhibiting sputa. Hence, proteomics and immunoproteomics revealed elevated levels of antimicrobial host proteins or S. pneumoniae antigen-specific IgGs in pneumococcal growth-inhibiting sputa, thus explaining their anti-pneumococcal activity.

**IMPORTANCE** Respiratory pathogens like Streptococcus pneumoniae can cause severe pneumonia. Nonetheless, mechanically ventilated intensive care patients, who have a high risk of contracting pneumonia, rarely develop pneumococcal pneumonia. This suggests the presence of potentially protective antimicrobial agents in their lung environment. Our present study shows for the first time that bronchoalveolar aspirates, “sputa,” of ventilated patients in a Dutch intensive care unit were characterized by three distinct groups of proteome abundance signatures that can explain their anti-pneumococcal activity. Importantly, this anti-pneumococcal sputum activity was related either to elevated levels of antimicrobial host proteins or to antibiotics and S. pneumoniae-specific antibodies. Further, the sputum composition of some patients changed over time. Therefore, we conclude that our study may provide a novel tool to measure changes that are indicative of infection-related conditions in the lungs of mechanically ventilated patients.

## INTRODUCTION

Mechanically ventilated patients are at risk of developing pneumonia. This relates to ineffective clearance of the lungs due to the insertion of an endotracheal tube, the use of narcotics, suppressed coughing, and the supine position of the patient (1–3). As a consequence, the lung alveoli of mechanically ventilated patients may fill with fluid, mucus, and pus, especially once pneumonia develops upon colonization and infection of the lungs by opportunistic pathogens (http://www.who.int/news-room/fact-sheets/detail/pneumonia). To prevent pneumonia and other infections, patients may be subjected to prophylactic systemic antimicrobial therapy (e.g., with cephalosporins), and selective decontamination of the digestive tract with nonabsorbable antimicrobial agents (e.g., tobramycin, colistin, and amphotericin B) (3, 4). In addition, the accumulating fluid is routinely aspirated to support respiration and increase patient comfort (3).

Antimicrobial activity in the upper and lower airways may protect patients against pneumonia. Therefore, we have previously performed a systematic analysis of antimicrobial activity in the bronchoalveolar aspirate (here referred to as “sputum”) that was collected from 53 mechanically ventilated patients at the Department of Critical Care of the University Medical Center Groningen (UMCG) (5). Of note, only one third of the included patients developed pneumonia, suggesting that the majority of these patients were effectively protected against pulmonary infection. Antimicrobial activity in the collected sputa was tested on two renowned causative agents of pneumonia, Streptococcus pneumoniae and Staphylococcus aureus, and on a sputum-resident Streptococcus anginosus isolate. Intriguingly, the detected antimicrobial activity in the investigated sputa was particularly effective against S. pneumoniae, and it could only be partially explained by antibiotic therapy as evidenced by quantification of cefotaxime concentrations in sputa of patients who had received this antibiotic as a monotherapy (*n* = 25) or in combination with ciprofloxacin (*n* = 3) (5). Furthermore, the detected antimicrobial activity could not be correlated with bacteriocin production by the sputum-resident microbiota (5). This raised the intriguing question whether host factors, such as human antimicrobial peptides and proteins, contribute to the detected anti-pneumococcal activity in the investigated sputa. Importantly, this would explain why pneumococcal pneumonia in mechanically ventilated patients is relatively rare, despite the fact that S. pneumoniae is one of the major causative agents of severe respiratory tract infections (6–9).

The aim of the present study was to investigate whether the sputa from mechanically ventilated patients show particular proteome and immunoproteome signatures that can be associated with the presence or absence of anti-pneumococcal activity. To this end, 36 sputa from 27 patients included in our previous study (5) were subjected to proteome analysis. All of these 27 patients had received cefotaxime systemically. Nonetheless, only 16 of the investigated sputum samples showed anti-pneumococcal activity, whereas this activity was undetectable in the 20 other samples (5). The results of our present study indicate that the sputa with or without anti-pneumococcal activity displayed distinct proteome signatures. Relatively high levels of proteins involved in innate immune responses characterize sputa inhibiting growth of S. pneumoniae but containing low or undetectable levels of cefotaxime. In contrast, S. pneumoniae-inhibiting sputa with relatively high cefotaxime concentrations and noninhibiting sputa were characterized by relatively high levels of proteins involved in inflammatory activities. Remarkably, S. pneumoniae-inhibiting sputa with relatively high cefotaxime concentrations displayed high anti-pneumococcal immunoglobulin G (IgG) titers.

## RESULTS

### Differential protein abundance profiles distinguish three groups of sputum samples.

**FIG 1:**
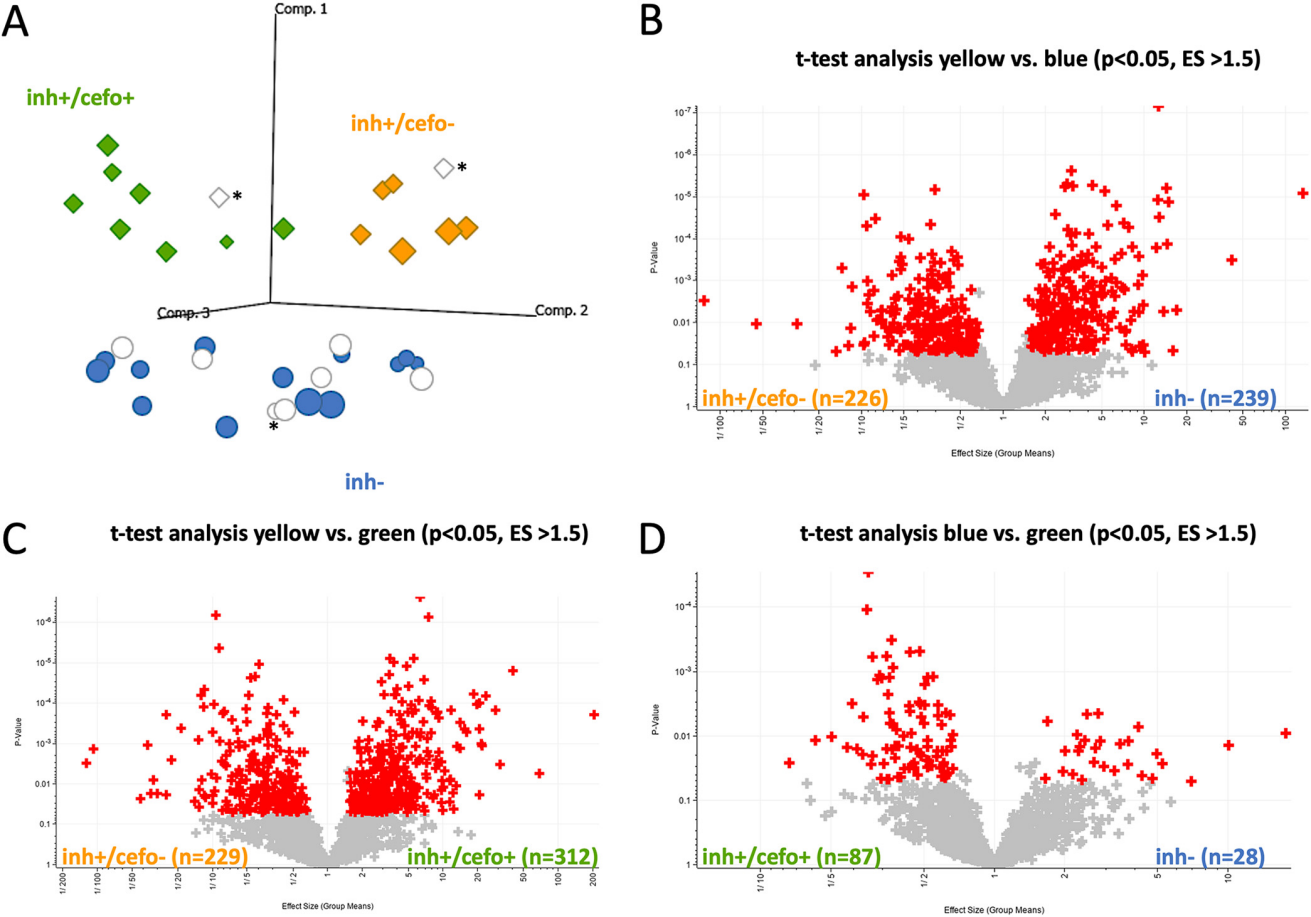
Partial least squares (PLS) analysis of sputum proteomes. Sputum samples from ICU patients were mechanically disrupted and subjected to LC-MS/MS measurements after processing as indicated in Materials and Methods. Proteins were identified using Genedata Refiner MS in combination with Mascot. Genedata Analyst was used for statistical analysis. (A) The PLS analysis distinguishes between the predefined sputum samples that inhibit growth of S. pneumoniae (diamonds) and noninhibiting sputum samples (circles). Further, two groups of S. pneumoniae-inhibiting sputum samples were distinguished that are characterized by cefotaxime concentrations above (green) or below (yellow) the MIC for S. pneumoniae. Of note, several noninhibiting samples contained cefotaxime concentrations above the MIC for S. pneumoniae (blue circles), while others contained cefotaxime below the MIC (white circles). Samples for which the presence of cefotaxime was not determined are indicated by an asterisk. Comp., component. (B to D) Volcano plots showing proteins that were present at different levels in the three sample groups identified by PLS. Each plus sign refers to a single protein, red plus signs indicate proteins that were significantly different in the respective group (*t* test, *P* value ≤ 0.05, effect size [ES] ≥ 1.5). All identified proteins, effect sizes, and *P* values are listed in Table S3 in the supplemental material.

**FIG 2:**
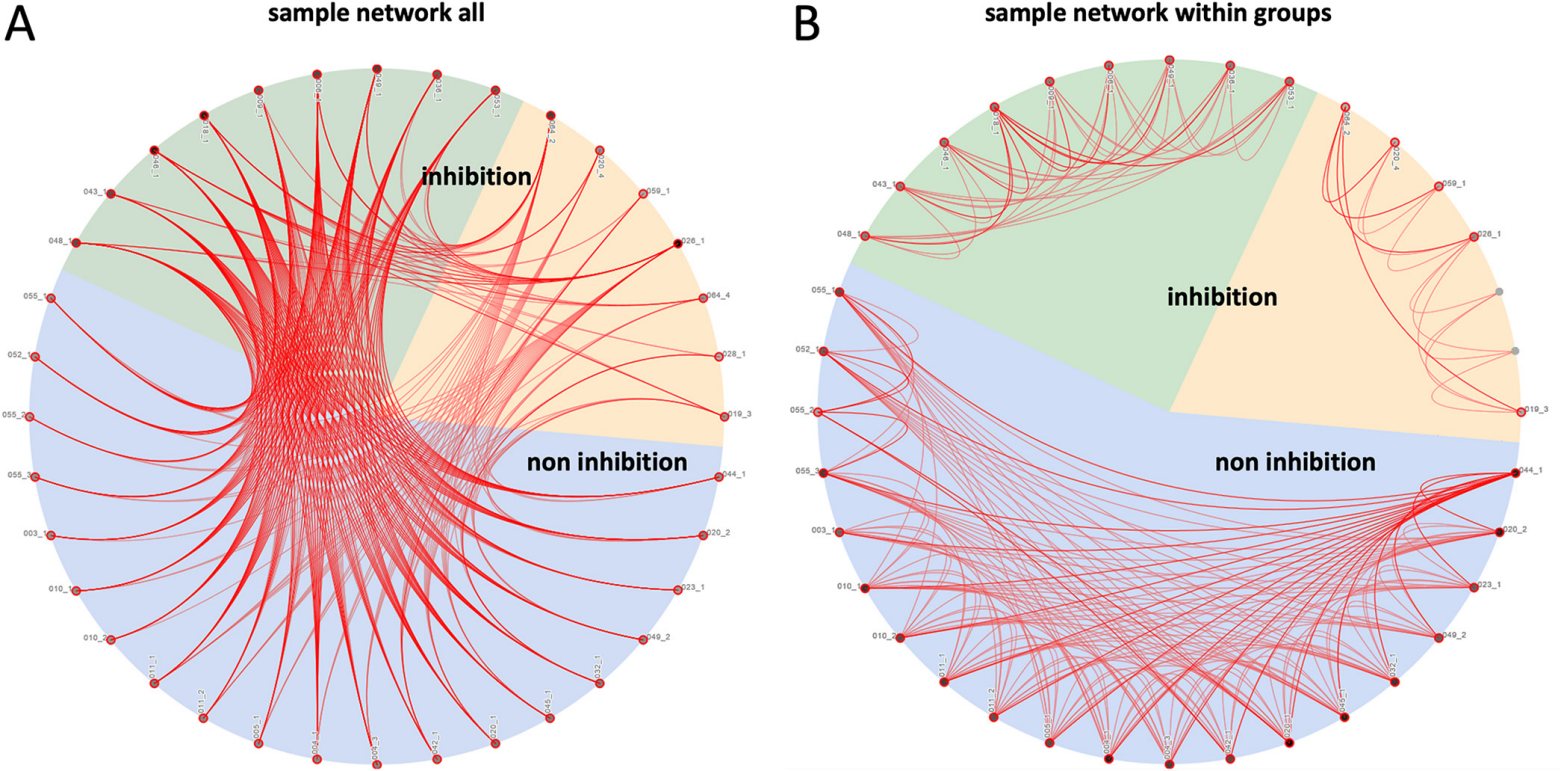
Correlation network of sputum samples. Possible correlations between the proteome data for different investigated sputa were analyzed by Genedata Analyst. The background colors in the pie charts refer to the sample groups identified by PLS, as shown in Fig. 1A. The red lines show correlations between sputum samples. (A) Network that shows how all sputum samples correlate. (B) Network that shows how all sputum samples correlate within their sample group.

**FIG 3:**
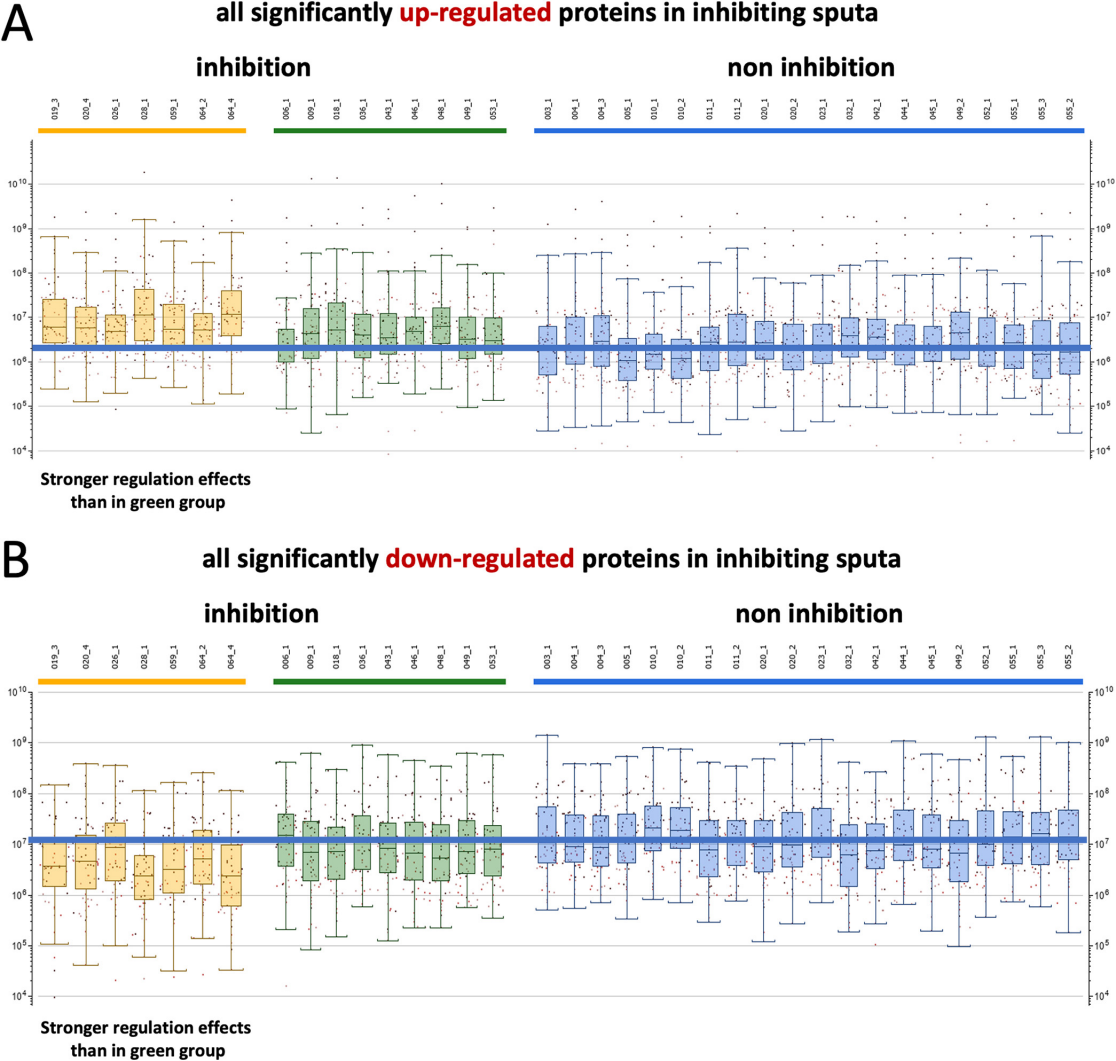
Differential protein levels per sputum sample and PLS sputum sample group. All proteins present at significantly elevated or lowered levels in inhibiting and noninhibiting sputum samples (see also Fig. S2 and Table S3) were plotted according to the sample groups identified by PLS using the green, yellow, and blue color code as described in the legend to Fig. 1A. This analysis highlights higher differential protein levels for the inh+/cefo− sputum sample group compared to the inh+/cefo+ sputum sample group. (A) Proteins present at elevated levels in inhibiting sputum samples. (B) Proteins present at lowered levels in inhibiting sputum samples. In both panels A and B, the 20 most regulated proteins per sputum sample are indicated as dots.

To assess the sputum proteome of mechanically ventilated patients, all of whom had received cefotaxime systemically as monotherapy (*n* = 24) or in combination with ciprofloxacin (*n* = 3) (Table 1), the proteins in these sputum samples were extracted and subjected to liquid chromatography coupled to tandem mass spectrometry (LC-MS/MS) analysis. A total of 1,922 proteins was identified over 36 sputum samples tested. One outlier sample was excluded from further analysis because of low protein intensity values. The raw data were quantile normalized to make the row intensity distribution equal for all selected samples (see Fig. S1 in the supplemental material). Subsequent group annotations by partial least squares (PLS) allowed the maximization of intergroup variances of predefined sputum sample groups with (inh+) or without (inh−) antimicrobial activity against S. pneumoniae (Fig. 1A; see Fig. S2 and Table S2 in the supplemental material). Proteins whose abundance differed significantly for these two sputum sample groups were identified by *t* tests (*n* = 128; Fig. S2B). Strikingly, the PLS analysis distinguished two groups of S. pneumoniae-inhibiting sputum samples, thereby separating samples with quantified cefotaxime concentrations below (inh+/cefo−) or above (inh+/cefo+) the MIC for S. pneumoniae TIGR4 (Fig. 1A and Table S2). When available, multiple sputum samples from particular patients were analyzed, and in most cases, these samples were assigned to the same PLS-identified sample group. However, the sputum samples from patients 020 and 049 were distributed over the inh− and inh+/cefo− or the inh− and inh+/cefo+ sputum sample groups, respectively (Fig. S2A). Proteins that differed significantly in the three PLS-identified sputum sample groups (inh−, inh+/cefo−, and inh+/cefo+) are highlighted in red in the three volcano plots of Fig. 1B to D. The highest numbers of proteins detected with significantly different abundance (*n* = 465) were observed upon comparison of the inh+/cefo− (yellow) group with the inh− (blue) group (Fig. 1B), and comparison of the inh+/cefo− (yellow) group with the inh+/cefo+ (green) group (*n* = 541; Fig. 1C). Relatively few proteins with significantly different abundance were identified upon comparison of the inh+/cefo+ (green) group with the inh− (blue) group (*n* = 115; Fig. 1D). Further, a correlation network analysis showed that most of the investigated sputum samples shared common features (Fig. 2A). However, in accordance with the outcome of the PLS analysis, more correlations were observed between the inh+/cefo+ sample group (marked by green shading) and the inh− sample group (blue shading) than between these two groups and the inh+/cefo− sample group (yellow shading; Fig. 2B). To further differentiate the three distinct groups of sputum samples at the proteome level, the regulation effects observed for all proteins detected at significantly elevated or lowered levels in inhibiting or noninhibiting sputa were plotted per sample group as defined in the PLS analysis of Fig. 1. The resulting plots, as shown in Fig. 3, highlight higher differential protein abundance levels for the inh+/cefo− (yellow) sputa than for the inh+/cefo+ (green) or inh− (blue) sputa. Together, the proteome analyses separate the S. pneumoniae-inhibiting sputum samples with low cefotaxime levels from the other investigated sputum samples, suggesting that the former samples (inh+/cefo−) are enriched in host-derived proteins with anti-pneumococcal activity. Importantly, this separation cannot be explained by the previously observed pneumococcal growth inhibition or the quantified cefotaxime concentration alone as visualized by color coding in the PLS plots of Fig. S3.

**TABLE 1:** Baseline characteristics and clinical variables of included ICU patients (*n* = 27)^*a*^

Variable	*n* (%) or median [IQR] {range}
Gender	
Male	19 (70.4)
Female	8 (29.6)
Age, median (years)	55.0 [36.0–69.0] {20.0–85.0}
Hospital LOS (days)	19.0 [8.6–32.4] {0.8–69.6}
ICU LOS (days)	7.5 [4.9–19.5] {0.8–45.9}
Admission diagnosis	
Neurological	20 (74.1)
Respiratory	4 (14.8)
Medical	1 (3.7)
Cardiological	2 (7.4)
ICU outcome	
Hospital transfer	21 (77.8)
Deceased	5 (18.5)
Nursing home	1 (3.7)
Mech. Vent. (hours)	116.0 [78.0–285.0] {18.0–1057.0}
COPD	2 (7.4)
Pneumonia	9 (33.3)
SAPS II	50.0 [38.0–60.0] {19.0–72.0}
APACHE IV^*b*^	75.0 [56.8–84.3] {29.0–117.0}
I.V. antibiotics	27 (100)
SDD topical antibiotics	27 (100)
Corticosteroids	6 (22.2)
Leukocytes	
Sample^*c*^^,^^*d*^	13.0 [9.4–17.9] {7.1–30.0}
Lowest^*e*^	8.0 [6.6–9.5] {3.7–10.9}
Highest^*f*^	17.9 [15.1–22.9] {8.1–45.6}
CRP	
Sample^*c*^^,^^*d*^	74.0 [37.0–126.0] {1.8–319.0}
Lowest^*e*^	4.3 [1.2–31.0] {0.4–117.0}
Highest^*f*^	135.0 [101.0–196.0] {16.0–319.0}

a IQR, interquartile range; LOS, length of stay; ICU, intensive care unit; Mech. Vent., mechanical ventilation; COPD, chronic obstructive pulmonary disease; SAPS, simplified acute physiology score; APACHE, acute physiology and chronic health evaluation; I.V., intravenous; SDD, selective decontamination of the digestive tract; CRP, C-reactive protein.

b Available for 26 patients.

c Available for 25 patients.

d Leukocytes/CRP measured in blood at the time of first sputum sample collection.

e Lowest leukocytes/CRP measured in blood during ICU admission.

f Highest leukocytes/CRP measured in blood during ICU admission. Of note, SDD is applied for preventing secondary colonization by Gram-negative bacteria, Staphylococcus aureus, or yeasts, and it involves (i) oropharyngeal and gastrointestinal application of nonabsorbable antimicrobial agents (i.e., tobramycin, colistin, and amphotericin B), and (ii) prophylactic treatment to prevent infections by commensal respiratory pathogens through systemic administration of cephalosporins (in particular cefotaxime) during the first 4 days of a patient’s stay in the intensive care unit. Patients 003, 004, 006, 009, 010, 011, 019, 020, 023, 026, 028, 032, 036, 042, 043, 044, 045, 046, 048, 049, 052, 053, 055, and 064 received cefotaxime as a monotherapy; patients 005, 018 and 059 received cefotaxime in combination with ciprofloxacin as previously described (5).

10.1128/mSystems.00702-20.1 FIG S1 Nonlinear data normalization. The box plots show how the raw sputum proteome data of the different samples (A) were quantile-normalized and classified as inhibiting (orange) or noninhibiting sputa (blue) (B). One outlier sample was excluded from further analysis because of low protein intensity values. Download FIG S1, DOCX file, 0.2 MB. Copyright © 2021 Seinen et al. 2021 Seinen et al. https://creativecommons.org/licenses/by/4.0/ This content is distributed under the terms of the Creative Commons Attribution 4.0 International license.

10.1128/mSystems.00702-20.2 FIG S2 Comparison of proteins between inhibiting and noninhibiting sputum samples. (A) PLS graph as shown in Fig. 1A, including run labels. (B) Volcano plot showing the proteins with different abundance in inhibiting sputum samples (yellow/green) and noninhibiting sputum samples (blue). Each plus sign refers to a single protein, and red plus signs indicate proteins that were significantly different in that particular group (*t* test, *P* value ≤ 0.05, effect size [ES] ≥ 1.5) All identified proteins, effect sizes, and *P* values are listed in Table S3. Download FIG S2, DOCX file, 0.2 MB. Copyright © 2021 Seinen et al. 2021 Seinen et al. https://creativecommons.org/licenses/by/4.0/ This content is distributed under the terms of the Creative Commons Attribution 4.0 International license.

10.1128/mSystems.00702-20.3 FIG S3 Partial least squares (PLS) of inhibiting and noninhibiting sputum samples. Inhibiting sputum samples are indicated by diamonds, and noninhibiting sputum samples are indicated by circles. (A) PLS graph showing measured pneumococcal growth inhibition zones, as demonstrated with the purple color gradient. (B) PLS graph showing measured cefotaxime concentrations, as demonstrated by the orange/brown color gradient. Download FIG S3, DOCX file, 0.2 MB. Copyright © 2021 Seinen et al. 2021 Seinen et al. https://creativecommons.org/licenses/by/4.0/ This content is distributed under the terms of the Creative Commons Attribution 4.0 International license.

10.1128/mSystems.00702-20.7 TABLE S2 Overview of included patients and sputum samples. Data adapted from Seinen et al. (J. Seinen, W. Dieperink, S. A. Mekonnen, P. Lisotto, et al., Virulence 10:879−889, 2019, https://doi.org/10.1080/21505594.2019.1682797). The pneumococcal growth inhibition was determined by spotting sputum samples onto a lawn of S. pneumoniae TIGR4. The cefotaxime concentration in patient sputa was quantified by high-performance liquid chromatography (HPLC)-MS/MS, the minimal inhibitory concentration of cefotaxime for S. pneumoniae TIGR4 is 0.015 μg/ml (Seinen et al.). A superscript # indicates that the respective sputum sample did not separate into pellet and supernatant fractions upon centrifugation. ND*, not determined; ND**, no cefotaxime detected. Download Table S2, DOCX file, 0.03 MB. Copyright © 2021 Seinen et al. 2021 Seinen et al. https://creativecommons.org/licenses/by/4.0/ This content is distributed under the terms of the Creative Commons Attribution 4.0 International license.

10.1128/mSystems.00702-20.8 TABLE S3 Proteomics data. The table shows the shotgun MS data analysis with Genedata Analyst (v13.0.1). Comparisons were made between the sputum sample groups inh+ (red) versus inh− (blue), and the PLS-defined sputum sample groups inh+/cefo− (yellow), inh+/cefo+ (green), and inh− (blue). The relative abundance of proteins was considered significant with a *P* value ≤ 0.05 and an effect size (ES) of ≥1.5, based on a *t* test. Only the proteins that show a significantly different abundance are marked with the indicated color codes. Cells are empty when the 40% valid value filter for statistical analysis was not reached. Proteins present at significantly higher abundance in the inh+/cefo− and inh− that were used for the STRING analysis presented in Fig. 9 are marked with the respective yellow and blue color codes. Download Table S3, XLSX file, 0.6 MB. Copyright © 2021 Seinen et al. 2021 Seinen et al. https://creativecommons.org/licenses/by/4.0/ This content is distributed under the terms of the Creative Commons Attribution 4.0 International license.

### Distinctive proteome abundance signatures of sputa.

**FIG 4:**
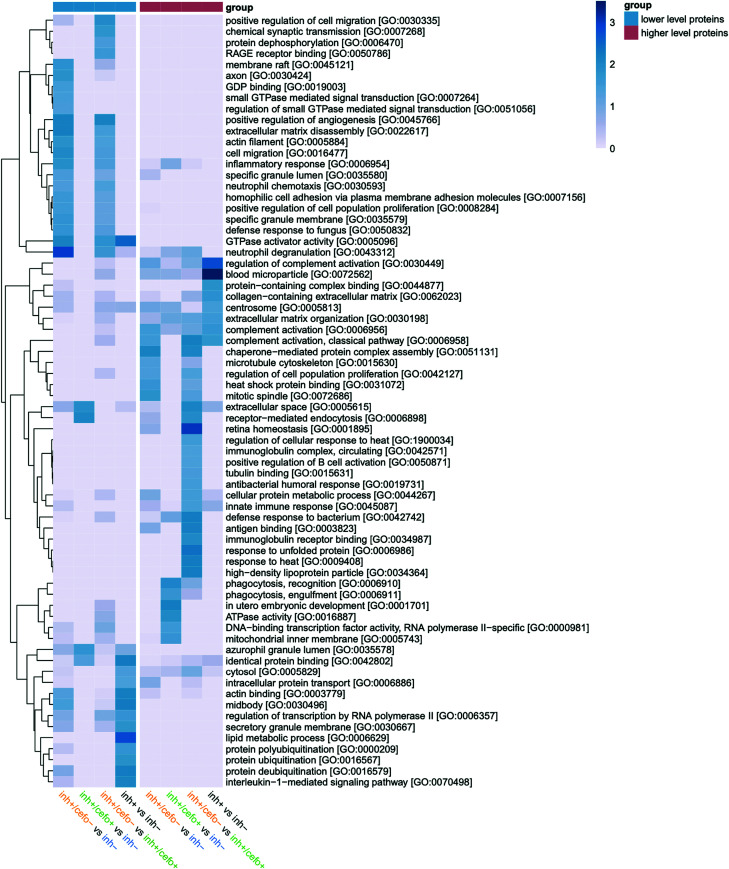
Enrichment analysis of the distinctive proteome signatures. An enrichment analysis was performed based on Gene Ontology (GO). The heatmap shows the enrichment of particular protein groups in the inh+/cefo+, inh+/cefo−, or inh− sputum sample groups distinguished by PLS versus the total set of presently identified sputum proteins. In addition, the heatmap shows the enrichment of particular protein groups in inhibiting sputum samples or noninhibiting sputum sample groups versus the total set of presently identified sputum proteins. The heatmap was generated based on the proteins identified at statistically significant relative abundance levels (by *t* tests), as presented in Fig. 1B to D and Fig. S2B. Of note, only the statistically significant enrichment of protein groups in a particular sputum sample group is represented according to the blue-scale color key depicted on the right side of the heatmap (−log_10_ *P* value of ≥1.3, equal to *P* value ≤ 0.05). The sample group color key (blue-red) indicates lower or higher levels of proteins in the first mentioned group within the comparison. The heatmap displays protein groups from the GO terms Cellular Compartment, Biological Process, and Molecular Function.

To identify protein functions that characterize the different PLS-identified sputum sample groups (Fig. 1), an overview enrichment analysis was performed, and the results are presented in Fig. 4. This analysis assigns individual proteins identified in differential abundance to different cellular compartments, biological processes, and molecular functions based on Gene Ontology (GO) terms. Of note, due to the classification by GO, some proteins were attributed to more than one GO term. The resulting heatmap in Fig. 4 compares the enrichment of particular protein groups of each distinguished sputum sample group with the total set of presently identified sputum proteins. Only the statistically significant enrichment of protein groups in a particular sputum sample group is shown in the heatmap. This highlights functional differences between sputum sample groups which, as ranked by possible implication in human host defenses against pathogens and declining *P* value, can be summarized as follows.

### (i) inh+ versus inh−.

Pneumococcal growth-inhibiting sputum samples were enriched in proteins related to complement activation, innate immune responses, and extracellular matrix organization (GO biological processes). In addition, the inhibiting sputum samples were enriched in proteins with GO molecular functions related to “protein-containing complex binding” (Fig. 4). The noninhibiting samples were found to be enriched in proteins related to various GO-defined pathways, including protein ubiquitination processes, the interleukin-1-mediated signaling pathway, and lipid metabolic processes. In GO terms of molecular function, noninhibiting sputum samples were enriched in proteins related to GTPase activator activity, identical protein binding, and actin binding (Fig. 4).

### (ii) inh+/cefo− versus inh−.

The inh+/cefo− sputum samples were enriched in proteins related to various biological processes, such as complement activation, chaperone-mediated protein complex assembly, and regulation of cell population proliferation. In terms of GO molecular functions, the inh+/cefo− sputum samples were enriched in proteins related to antigen binding and heat shock protein binding. Conversely, inh− sputum samples were enriched in proteins related to neutrophil degranulation and chemotaxis, inflammatory responses, extracellular matrix (ECM) disassembly, and positive regulation of angiogenesis. With regard to proteins involved in GO-defined molecular functions, the inh− samples were enriched in proteins related to GTPase activator activity and GDP binding (Fig. 4).

### (iii) inh+/cefo+ versus inh−.

In this comparison, the inh+/cefo+ sputum samples displayed enrichment in proteins related to phagocytosis, the defense response to bacteria, and also slightly in ECM organization. In GO terms of molecular function, the inh+/cefo+ sputum samples were enriched in proteins related to ATPase activity and DNA-binding transcription factor activity. On the other hand, the inh− sputum samples were enriched in proteins related to receptor-mediated endocytosis (biological processes) and identical protein binding (molecular function; Fig. 4).

### (iv) inh+/cefo− versus inh+/cefo+.

Compared to inh+/cefo+ sputum samples, the inh+/cefo− sputum samples were enriched in proteins involved in biological processes, such as the defense response to bacteria, complement activation, and responses to heat- and receptor-mediated endocytosis. Regarding molecular function, the inh+/cefo− sputum samples were enriched in proteins related to antigen binding, immunoglobulin receptor binding, tubulin binding, and heat shock protein binding. Conversely, the inh+/cefo+ sputum samples were enriched in proteins related to positive regulation of angiogenesis, ECM disassembly, cell migration, inflammatory responses, neutrophil chemotaxis, and degranulation (biological processes). Further, the inh+/cefo+ samples were enriched in proteins related to RAGE receptor binding and GTPase activator activity (molecular function; Fig. 4).

### Differential abundance of antimicrobial proteins.

**FIG 5:**
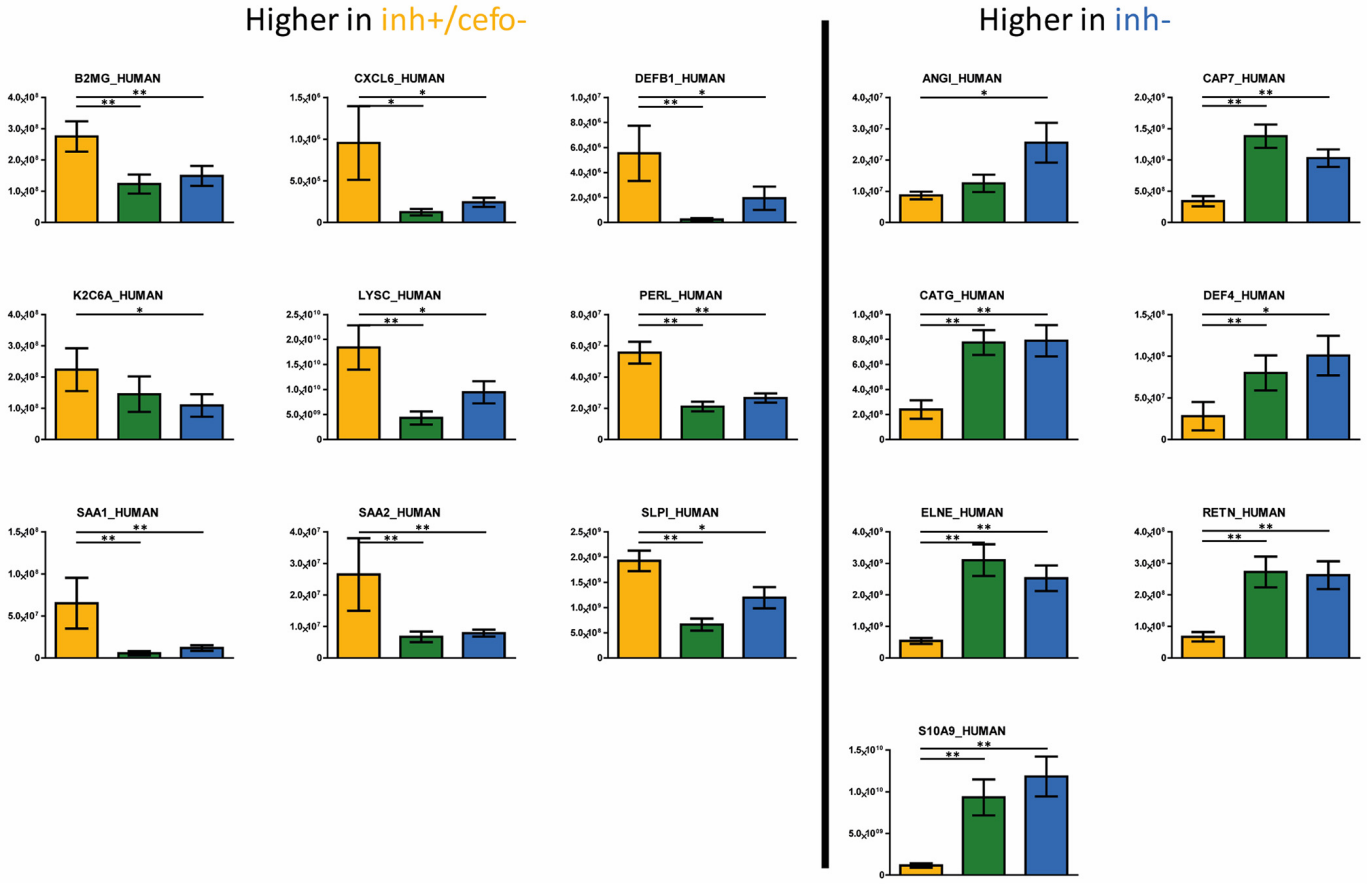
Differential abundance of antimicrobial sputum proteins and derivative peptides. The bar plot graphs show the intensities (expressed as means ± standard error of the means [SEM] [error bars]) of proteins or derivative peptides that are known to inhibit bacterial growth and viability. The yellow (inh+/cefo−), green (inh+/cefo+), and blue (inh−) bars refer to the sample groups identified with PLS, as shown in Fig. 1A. Only those proteins that showed statistically significant differential abundance in the inh+/cefo− and inh− sputum sample groups are included (*t* test, *P* value ≤ 0.05, effect size [ES] ≥ 1.5; Table S3). Asterisks mark *P* values of ≤0.05 (*) and ≤0.01 (**).

A key question that was immediately answered by our proteome analyses was whether the inh+ and inh− sample groups show significantly different abundances for proteins or derivate peptides with direct inhibitory potential against bacteria. As shown in Fig. 5, this was indeed the case. In particular, the significantly more abundant antimicrobial proteins in the inh+/cefo− group include β-defensin 1 (DEFB1), β-2-microglobulin (B2MG), C-X-C motif chemokine 6 (CXCL6), type II cytoskeletal 6A keratin (K2C6A), lysozyme (LYSC), lactoperoxidase (PERL), serum amyloid A-1 protein (SAA1), serum amyloid A-2 protein (SAA2), and antileukoproteinase (SLPI) (Fig. 5, left panel). Antimicrobial proteins with significantly higher abundance in the inh− sputum sample group include angiogenin (ANGI), azurocidin (CAP7), cathepsin G (CATG), neutrophil defensin 4 (DEF4), neutrophil elastase (ELNE), resistin (RETN), and protein S100-A9 (S10A9). Of note, most of the proteins present at significantly different levels in the inh+/cefo− and inh+/cefo+ sputum sample groups showed similar differences upon comparison of the inh+/cefo− and inh− sample groups (Fig. S4). In fact, no significant differences were detectable for proteins with known antimicrobial activity when comparing the inh+/cefo+ and inh− sample groups.

10.1128/mSystems.00702-20.4 FIG S4 Differential abundance of antimicrobial sputum proteins and derivative peptides. The bar plot graphs highlight the relative abundances of proteins or derivative peptides that are known to inhibit bacterial growth and viability. The yellow (inh+/cefo−), green (inh+/cefo+), and blue (inh−) bars refer to the sample groups identified with PLS, as shown in Fig. 1A. Only those proteins that showed statistically significant differential abundance in the inh+/cefo− and inh+/cefo+ sputum sample groups are included (t test, *P* value ≤0.05, effect size [ES] ≥1.5; Table S3). Asterisks mark *P* values of ≤0.05 (*) and ≤0.01 (**). Download FIG S4, DOCX file, 0.5 MB. Copyright © 2021 Seinen et al. 2021 Seinen et al. https://creativecommons.org/licenses/by/4.0/ This content is distributed under the terms of the Creative Commons Attribution 4.0 International license.

### Differential abundance of complement-associated proteins.

**FIG 6:**
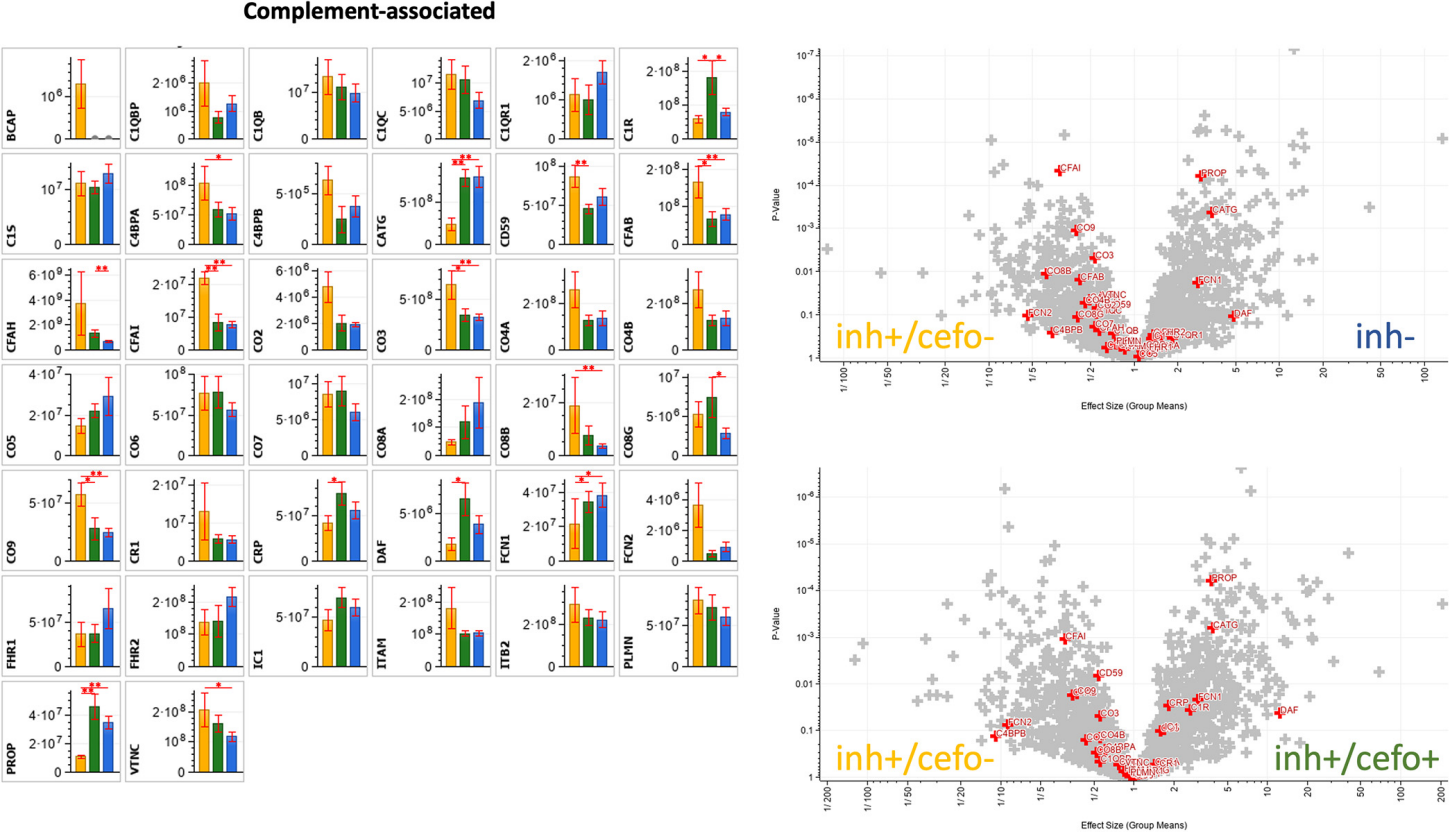
Differential abundance of complement-associated proteins. The bar plot graphs and volcano plots highlight the relative abundances of all complement-associated proteins identified in the current sputum proteome data set. The bar plot graphs show the protein intensities (expressed as means ± SEM [error bars]) of the respective proteins. The yellow (inh+/cefo−), green (inh+/cefo+), and blue (inh−) bars refer to the sample groups identified with PLS, as shown in Fig. 1A. The volcano plots show all proteins with differential abundance in the different sample groups. The red plus signs refer to the complement-associated proteins, as detailed in the bar plots. *P* values and effect sizes for differential abundance of individual proteins are presented in Table S3. Asterisks mark *P* values of ≤0.05 (*) and ≤0.01 (**).

Following the functional enrichment as presented in Fig. 4, a more detailed inspection of proteins implicated in infection-related processes was performed. A finding that attracted special attention concerned the differential abundance of complement-related proteins. Figure 6 presents the relative abundance of complement-associated proteins per PLS-defined sputum sample group, as depicted by yellow (inh+/cefo−), green (inh+/cefo+), and blue (inh−) bars. Proteins shown are related to the classical complement pathway (e.g., C1QB, C1QC, C1R, and C1S), the lectin pathway (e.g., FCN1 and FCN2), or the alternative pathway (e.g., CFAB and CO3). In addition, proteins downstream of the classical, lectin and/or alternative pathways (e.g., CO2, CO4A, CO4B, CO5, and PROP), the terminal complement complex (CO6, CO7, CO8A, CO8B, CO8G, and CO9) and proteins involved in the regulation of complement activity (e.g., C1QBP, C4BPA, C4BPB, CD59, CFAH, CFAI, CR1, DAF, IC1, and PLMN) are shown. Some of the identified proteins are related in other ways to complement activity (e.g., BCAP, C1QR1, CATG, CRP, FHR1, FHR2, ITAM, ITB2, and VTNC) (10–13).

The volcano plots in Fig. 6 show all proteins with differential abundance in the different sample groups. Especially, the C4BPA, CFAB, CFAI, CO3, CO8B, CO9, and VTNC proteins were significantly more abundant in the inh+/cefo− sputum sample group than in the inh− sputum sample group. Conversely, the proteins CATG, FCN1, and PROP were significantly more abundant in the inh− sputum samples than in the inh+/cefo− sputum samples (Fig. 6). The CD59, CFAB, CFAI, CO3, and CO9 proteins were significantly more abundant in the inh+/cefo− sputum samples than in the inh+/cefo+ sputum samples. Further, the C1R, CATG, CRP, DAF, FCN1, and PROP proteins were more abundant in the inh+/cefo+ sputum samples than in the inh+/cefo− sputum samples (Fig. 6). Most of the complement-associated proteins showed no significant differential abundance in the inh+/cefo+ and inh− sputum samples, except for the C1R, CFAH, and CO8G proteins, which were more abundant in inh+/cefo+ sputum samples than in inh− sputum samples (Fig. 6). Interestingly, some of the relative abundancy differences would not have been noticed, if the inh+/cefo− and inh+/cefo+ samples had only been grouped as inh+ samples, as exemplified by the CFAB and VTNC proteins. Altogether, most of the complement-associated proteins were detected in the inh+/cefo− sputum sample group. This implies an activation of the complement system in the respective patients at the moment of sampling.

### Differential abundance of neutrophil elastase-associated proteins.

**FIG 7:**
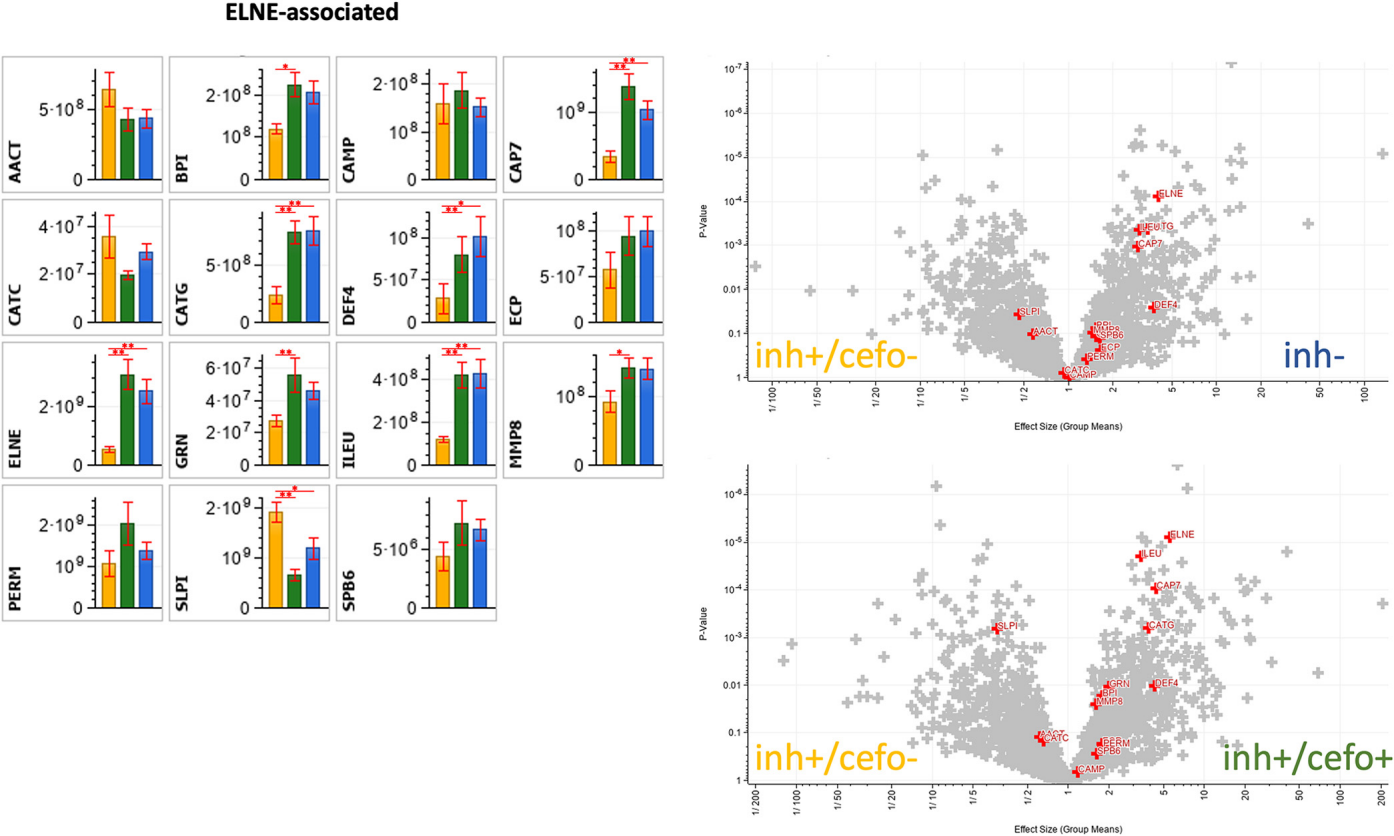
Differential abundance of ELNE-associated proteins. The bar plot graphs and volcano plots highlight the relative abundances of all ELNE-associated proteins identified in the current sputum proteome data set. The bar plot graphs show the protein intensities (expressed as means ± SEM) of the respective proteins. The yellow (inh+/cefo−), green (inh+/cefo+), and blue (inh−) bars refer to the sample groups identified with PLS, as shown in Fig. 1A. The volcano plots show all proteins with differential abundance in the different sample groups. The red plus signs refer to the ELNE-associated proteins, as detailed in the bar plots. *P* values and effect sizes for differential abundance of individual proteins are presented in Table S3. Asterisks mark *P* values of ≤0.05 (*) and ≤0.01 (**).

Another set of proteins that was explored in more detail are neutrophil elastase-associated proteins, referred to as ELNE (Fig. 7). Elastase is a serine protease, stored in azurophilic granules of neutrophils. Intracellularly, it is known to degrade outer membrane proteins of Gram-negative bacteria and bacterial virulence factors. Extracellularly, it degrades extracellular matrix components, such as elastin, vitronectin, and type IV collagen, and it is involved in various aspects of inflammation (14, 15). The ELNE-associated proteins in Fig. 7 can be categorized as antimicrobial (including proteases) (e.g., BPI, CAMP, CAP7, CATG, DEF4, ECP, ELNE, MMP8, and SLPI), protease-inhibiting (e.g., AACT, ILEU, SLPI, and SPB6) and proteins with other related activities (e.g., CATC, GRN, and PERM) (12).

The volcano plots in Fig. 7 highlight the differential distribution of ELNE-associated proteins over the PLS-defined sample groups. In particular, the SLPI protein was significantly more abundant in the inh+/cefo− sputum sample group than in the inh− sputum sample group. In contrast, the CAP7, CATG, DEF4, ELNE, and ILEU proteins were significantly more abundant in the inh− sputum samples than in the inh+/cefo− sputum samples (Fig. 7). The SLPI protein was also more abundant in the inh+/cefo− sputum samples than in the inh+/cefo+ sputum samples, whereas the BPI, CAP7, CATG, DEF4, ELNE, GRN, ILEU, and MMP8 proteins were more abundant in the inh+/cefo+ sputum samples than in the inh+/cefo− sputum samples (Fig. 7). Similar to the antimicrobial proteins, none of the ELNE-associated proteins showed significant differences between inh+/cefo+ and inh− sputum samples. Thus, the combined data reveal a higher relative abundance of ELNE-associated proteins in the inh− and inh+/cefo+ sputum samples than in the inh+/cefo− samples (Fig. 7).

### Differential abundance of apolipoprotein-associated proteins.

**FIG 8:**
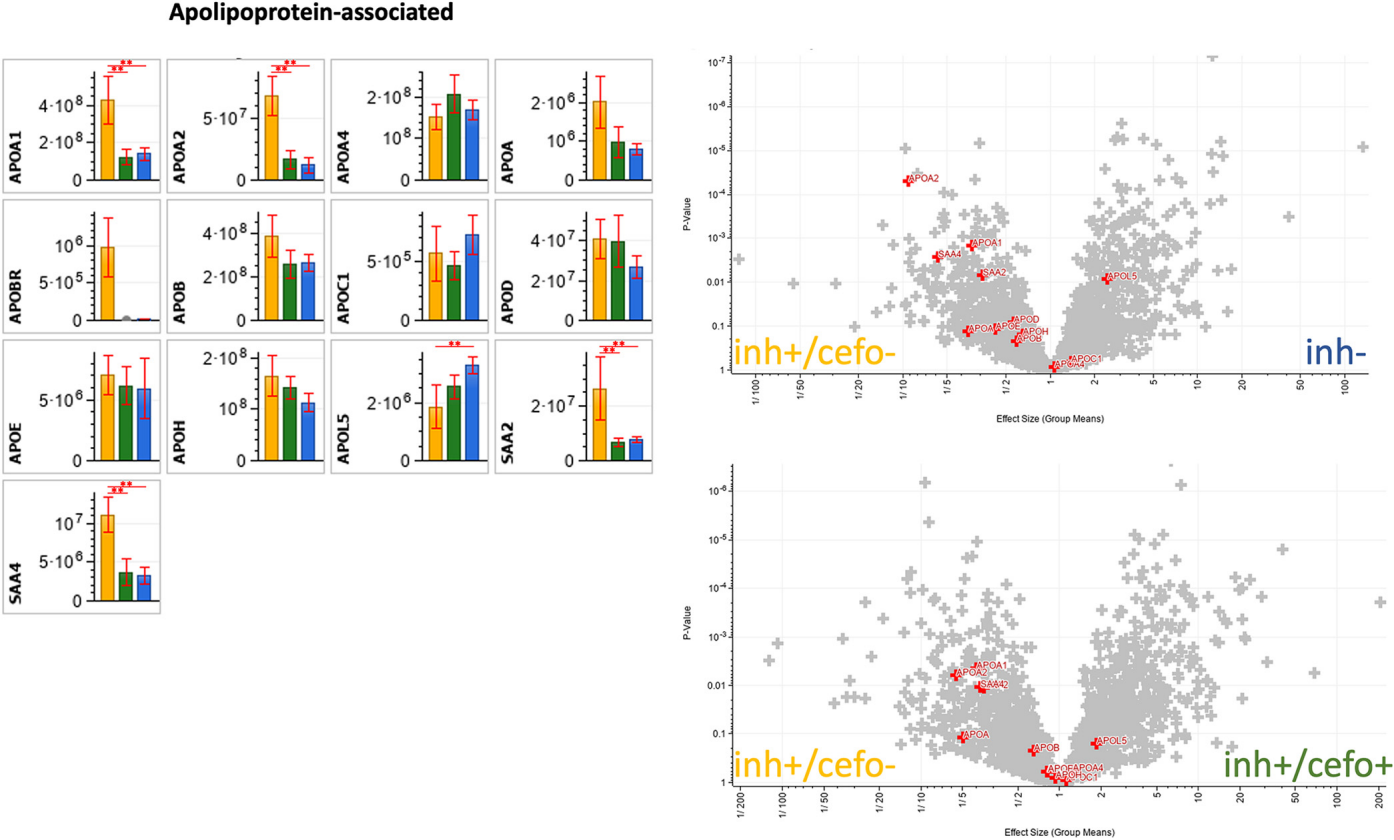
Differential abundance of apolipoprotein-associated proteins. The bar plot graphs and volcano plots highlight the relative abundances of all apolipoprotein-associated proteins identified in the current sputum proteome data set. The bar plot graphs show the protein intensities (expressed as means ± SEM) of the respective proteins. The yellow (inh+/cefo−), green (inh+/cefo+), and blue (inh−) bars refer to the sample groups identified with PLS, as shown in Fig. 1A. The volcano plots show all proteins with differential abundance in the different sample groups. The red plus signs refer to the apolipoprotein-associated proteins, as detailed in the bar plots. *P* values and effect sizes for differential abundance of individual proteins are presented in Table S3. Asterisks mark *P* values of ≤0.05 (*) and ≤0.01 (**).

Figure 4 also showed the enrichment of lipoprotein particles in the inh+/cefo− sample group compared to the inh+/cefo+ sample group. In contrast, inh− sputum samples were enriched in proteins involved in lipid metabolic processes compared to the inh+ sputum samples. In general, lipoproteins serve major functions in the human lipid metabolism. The lipoproteins in plasma can be classified based on lipid and apolipoprotein composition, as well as size (16, 17). Apolipoproteins can be grouped by their functions, namely, (i) structural roles (e.g., APOA1, APOA2, and APOB in Fig. 8); (ii) lipoprotein receptor ligands (e.g., APOB and APOE); (iii) directing lipoprotein formation; and (iv) activation or inhibition of enzymes implicated in lipoprotein metabolism (e.g., APOA1, APOA4, and APOC1) (16, 17). Importantly, certain apolipoproteins are known to play a role in lung disease (18, 19), and apolipoproteins have also been implicated in innate immune defenses against pathogens and the modulation of bacterial virulence by sequestering quorum-sensing peptides (20–22). Figure 8 shows the differential abundance of apolipoprotein-associated proteins in the three PLS-defined sputum sample groups.

As shown by the bar diagrams and volcano plots in Fig. 8, the APOA1, APOA2, SAA2, and SAA4 proteins were significantly more abundant in the inh+/cefo− sputum sample group than in the inh− sputum sample groups. Of note, SAA2 is known to possess antimicrobial activity (23). The APOL5 protein was significantly more abundant in the inh− sputum samples than in the inh+/cefo− sputum samples (Fig. 8). The APOA1, APOA2, SAA2, and SAA4 proteins were more abundant in the inh+/cefo− sputum samples than in the inh+/cefo+ sputum samples. In contrast, none of the apolipoprotein-associated proteins were more abundant in the inh+/cefo+ sputum samples than in the inh+/cefo− sputum samples (Fig. 8), and between the inh+/cefo+ and inh− sputum samples groups, no significant differences were detected for apolipoprotein-associated proteins (Fig. 8). Altogether, apolipoprotein-associated proteins were present at higher levels in the inh+/cefo− sputum samples than in the inh+/cefo+ or inh− sputum samples (Fig. 8).

### Sputum protein network complexity.

**FIG 9:**
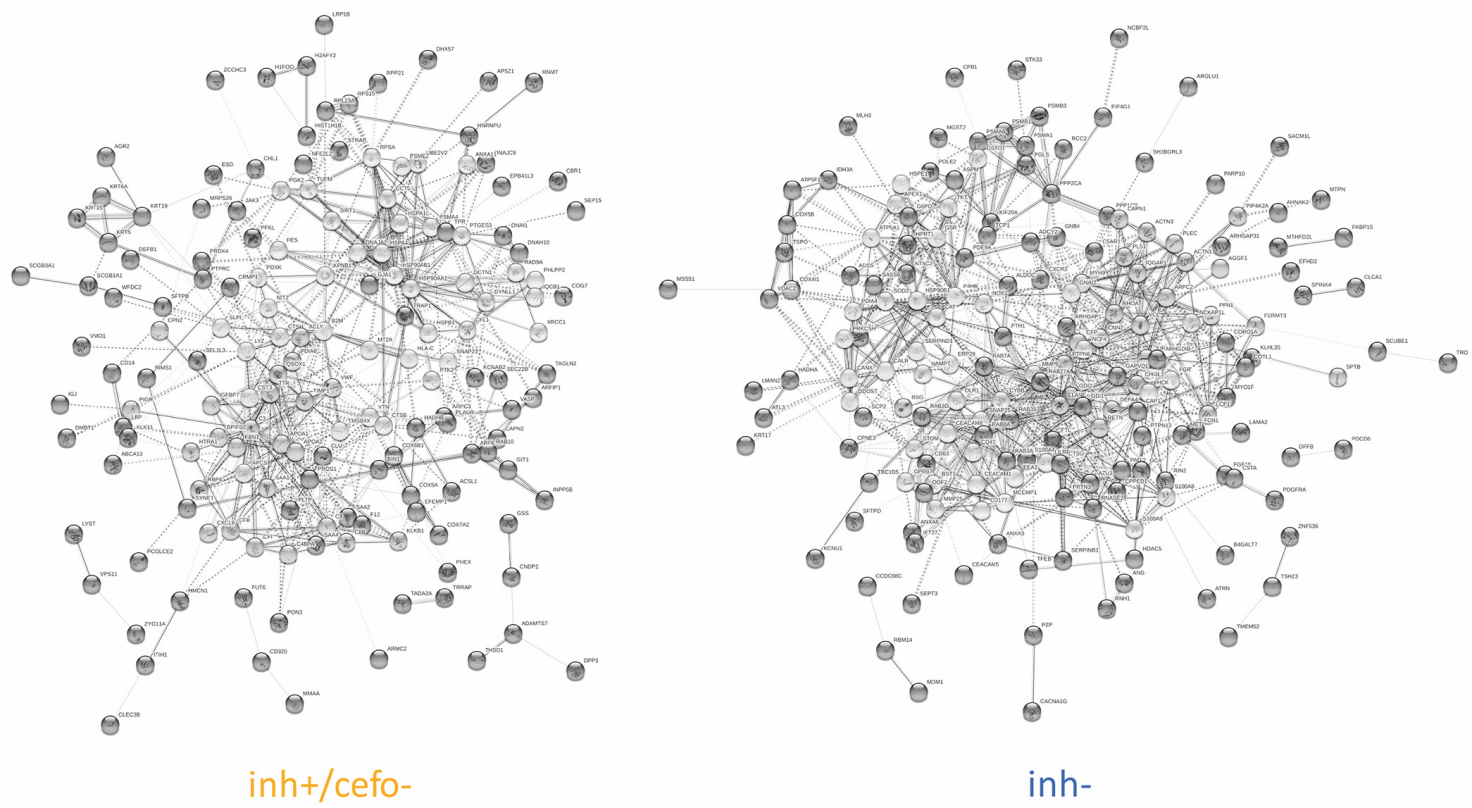
STRING networks from the inh+/cefo− versus inh− sputum sample groups. The two STRING networks depict interrelationships between proteins shown to be present at significantly higher abundance in the inhibiting sputum samples with a cefotaxime concentration below the MIC (inh+/cefo−) (left) and the noninhibiting sputum samples (inh−) (right). Individual proteins are represented as spheres, labeled with the respective gene names as listed in Table S3. Of note, human proteins identified by our proteome analysis, but not included in the STRING database, are not represented in the two networks. Identified proteins that are not connected with the network are excluded.

To verify associations between proteins identified with differential abundance in the PLS-defined sample groups, a STRING protein-protein network analysis was performed based on the data presented in Table S3. As shown in Fig. 9, the more abundant proteins in the inh+/cefo− sputum samples (A) or the inh− samples (B) share many interrelationships, as judged by the respective STRING scores that reflect the confidence in particular protein-protein interactions in the network.

### Quantification of IgG antibodies against pneumococcal antigens.

**FIG 10:**
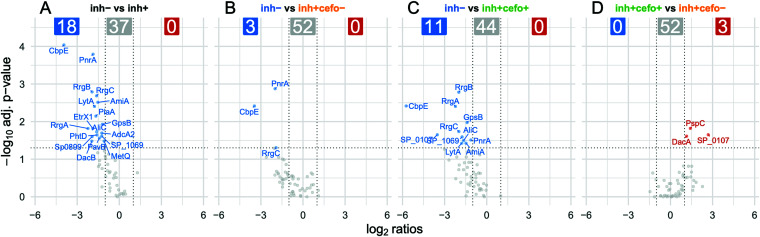
Sputum IgG responses against 55 pneumococcal antigens. The Luminex xMAP technology and xMAPr app were used to quantify the levels of sputum IgGs specific for 55 S. pneumoniae antigens. Each volcano plot refers to a comparison between different sputum sample groups as indicated. Results are based on the log_2_ ratios, with an absolute fold change cutoff of 2.0, and an adjusted *P* value of 0.05. Red-labeled antigen names refer to IgGs present at higher levels in the sample group mentioned first within each comparison, and blue-labeled antigen names refer to IgGs present at higher levels in the sample group mentioned last within a comparison. Gray dots refer to IgGs that are present at similar levels in the two sample groups compared. The response data per antigen are detailed in Fig. S5.

As shown in Fig. 4, the inh+/cefo− sputum samples were also enriched in protein groups related to the humoral immune response. This suggested that differences in antibody titers also contribute to the differences in pneumococcal growth inhibition as observed for the different sputum sample groups. Therefore, IgG titers against 55 different pneumococcal antigens were quantified in the sputum samples using the Luminex xMAP technology as presented in Fig. 10. The first volcano plot shows that the IgG responses against 18 pneumococcal antigens were significantly higher in the inhibiting sputum samples than in the noninhibiting samples, which do not contain anti-pneumococcal IgGs at higher levels (Fig. 10A). Subsequently, the differences in anti-pneumococcal IgG responses between the PLS-defined sample groups were also analyzed. The IgG responses against three antigens were significantly higher in the inh+/cefo− sample group than in the inh− sample group, with no elevated anti-pneumococcal IgG levels in the inh− sample group (Fig. 10B). In addition, the IgG responses against 11 pneumococcal antigens were higher in the inh+/cefo+ sample group than in the inh− sample group. Again, no IgG response was higher in the inh− samples (Fig. 10C). Last, when the IgG titers from the inh+/cefo+ samples were compared to those in the inh+/cefo− sputum samples, only the responses against three antigens were significantly higher in the inh+/cefo+ group (Fig. 10D). Figure S5 shows the individual boxplot graphs with the IgG responses against all 55 pneumococcal antigens tested in more detail for the three PLS-defined sputum sample groups. Altogether, these observations indicate that the anti-pneumococcal IgG responses could contribute to the observed pneumococcal growth inhibition, especially in the inh+/cefo+ sputum sample group.

10.1128/mSystems.00702-20.5 FIG S5 Individual boxplot graphs of quantified IgG levels against the respective pneumococcal antigens. The Luminex xMAP technology and xMAPr app were used to quantify the levels of sputum IgGs specific for 55 S. pneumoniae antigens. The sizes of the symbols in the boxplots are proportional with the previously measured diameters of pneumococcal growth inhibition zones in millimeters. The colors of the boxes refer to the PLS-identified sputum sample groups as in Fig. 1A. The symbol shape indicates whether the quantified cefotaxime concentration was below (circle, 0), or equal/above (triangle, 1) the MIC for S. pneumoniae TIGR4. (A) Boxplot showing the response for a particular antigen on a nonlog scale and the statistical outcome of the respective Wilcoxon rank sum test. (B) Boxplot with the response for a particular antigen on a log_10_ scale. Download FIG S5, PDF file, 1.8 MB. Copyright © 2021 Seinen et al. 2021 Seinen et al. https://creativecommons.org/licenses/by/4.0/ This content is distributed under the terms of the Creative Commons Attribution 4.0 International license.

## DISCUSSION

Essentially there are three ways in which S. pneumoniae can die within the human body. In the first place, autolysis is an important element in the life cycle of this pathogen (24), with the major virulence factor pneumolysin being massively released into the environment (25). Alternatively, pneumococcal killing can be facilitated by the innate and adaptive human immune defenses. For instance, the human body can produce a variety of compounds with antimicrobial activity against S. pneumoniae. These include antimicrobial peptides, such as defensins (26–28) and the cathelicidin-derived LL-37 peptide (29, 30). In addition, opsonization of S. pneumoniae by complement (31) and IgGs (32–35) will lead to killing by professional phagocytes (36, 37). Last, pneumococci may be eliminated through antibiotic therapy, such as cefotaxime. In the present study, we performed a proteomic analysis to identify proteins that distinguish sputa that kill S. pneumoniae from nonkilling sputa. For this purpose, we analyzed previously collected sputa from 27 mechanically ventilated patients who were systemically treated with cefotaxime (5).

Importantly, the previously quantified levels of the antibiotic cefotaxime or the presence of certain bacterial species in the collected sputa that might produce bacteriocins could not explain the observed antimicrobial activity in a large group of these samples (5). This raised the question whether the observed antimicrobial activity could be attributed to host factors, such as antimicrobial peptides and proteins. A selection of the previously investigated sputum samples was therefore subjected to proteome analysis. To this end, the investigated sputum samples were initially divided into inhibiting and noninhibiting samples. Remarkably, our present PLS analysis separated the inhibiting samples into two sputum sample groups. In short, it was shown that the inh+/cefo− sputum sample group had a proteome signature that was clearly distinct from the proteome signatures of the inh+/cefo+ and inh− sputum sample groups. The inh+/cefo− sputa were characterized by relatively high levels of proteins involved in innate immune responses, whereas inh+/cefo+ and inh− sputa were characterized by relatively high levels of proteins related to inflammatory processes. Moreover, inh+/cefo+ sputa contained relatively high levels of anti-pneumococcal IgGs.

In particular, the inh+/cefo− sputum samples were enriched in proteins related to the innate immune system, including complement-associated proteins and apolipoproteins. The complement system is of critical importance for the human defense against invading pathogens, and it also plays a role in homeostasis and inflammation (10, 11, 31). Complement could thus explain the observed pneumococcal growth inhibition by the inh+/cefo− sputum samples. However, based on the present proteome data alone, it is not possible to pinpoint particular complement-related proteins that might be responsible for the observed killing of S. pneumoniae. In addition, bacteria like S. pneumoniae have evolved strategies to evade the complement system and that allow them to cause invasive infections (31, 38, 39). This suggests that also other components of the sputum contributed to pneumococcal killing. In this case, the identified apolipoproteins might impact on pneumococcal quorum sensing, as previously described for S. aureus (20–22), and regulated autolysis. Importantly, we also identified other bactericidal proteins at elevated levels in the inh+/cefo− sputa, especially β-defensin 1, β-2-microglobulin, C-X-C motif chemokine 6, type II cytoskeletal 6A keratin, lysozyme, lactoperoxidase, the serum amyloid A-1 and A-2 proteins, and antileukoproteinase. Although S. pneumoniae may display resistance against some of these proteins, as exemplified by lysozyme (40), it is likely that several of these proteins combined with complement can severely affect the pneumococcal growth and viability.

The growth-inhibiting effect of the inh+/cefo+ sputum samples was initially attributed to the presence of cefotaxime at levels above the MIC (5). Consistent with this notion, the proteome of inh+/cefo+ sputum samples was most similar to that of the inh− sputum samples. Nonetheless, the inh+/cefo+ and inh− sputum samples were found to be enriched in proteins related to inflammation, including the ELNE-associated proteins. Neutrophil elastase is responsible for degrading microbial peptides and the ECM, and the identified ELNE-associated proteins are mostly antimicrobial proteins, proteases, or protease inhibitors. Generally, we observed that when the protease levels were relatively high (e.g., ELNE, CATG), the protease inhibitor levels were lower (e.g., AACT) in the inh+/cefo+ and inh− sputum samples. Notably, the identified (ELNE-associated) antimicrobial proteins do not seem to affect pneumococcal growth, as we found them at elevated levels in the non-growth-inhibiting sputa. This may relate to a reduced activity of antimicrobial peptides in sputum (41–44).

Another intriguing observation is that the highest levels of anti-pneumococcal IgGs were identified in the inh+/cefo+ sputum samples. This suggests that the pneumococcal growth-inhibiting activity in these samples is due to the elevated IgG levels in combination with the above-MIC cefotaxime levels. Inhibition of pneumococcal proteins by antibodies might thus be of particular importance. Interestingly, it was previously proposed that the cell cycle protein GpsB is conditionally essential for S. pneumoniae (45, 46). In addition, the genes for the LysM domain protein SP_0107, the lipoproteins l,d-carboxypeptidase (DacB) and pneumococcal nucleoside receptor A (PnrA), the choline-binding protein E (CbpE, also referred to as Pce), the conserved hypothetical protein SP_1069, the oligopeptide-binding protein AmiA, and the autolysin LytA were shown to be important, meaning that their deletion caused a particular phenotype in at least one infection-relevant condition (45). Proteins such as PnrA, RrgA, RrgB, and histidine triad protein (PhtD) were also shown to be immunogenic (47–49), suggesting a protective effect of IgGs targeting these proteins. Notably, in contrast to the inh+/cefo+ sputum samples, the anti-pneumococcal activity in the inh+/cefo− samples would be determined by the human host’s innate immune defenses.

Most pulmonary proteome analyses were thus far focused on patients with chronic obstructive pulmonary disease (COPD), asthma, cystic fibrosis, and/or the effects of smoking (50–54). Studies reviewed by Twigg et al. indicate that the balance of neutrophil-derived proteases and protease inhibitors is disturbed by the high load of neutrophil-derived proteases in sputa from cystic fibrosis patients. This can lead to inflammation, mucus hypersecretion, and impaired immune system regulation (55). Our present data reveal that also the sputa of mechanically ventilated ICU patients display distinct proteome signatures that can be related to anti-pneumococcal activity of the respective sputum samples. These findings could, at least in part, explain why pneumococcal pneumonia in mechanically ventilated patients is rather rare (6, 7). At present, we can only speculate why the sputa of mechanically ventilated patients show the different observed proteomic and immunoproteomic signatures. Conceivably, this relates to episodes of pathogen exposure before or during the patients’ hospitalization, their individual immune history, the conditions of their lungs, genetic predispositions, lifestyle factors, or combinations thereof. Nevertheless, we were so far not able to correlate the level of sputum antimicrobial activity to specific clinical metadata of the patients from whom the sputa were obtained (5). Possibly, patients with low cefotaxime levels in the lungs and low levels of anti-S. pneumoniae IgGs have a higher propensity to mount a complement-mediated immune response as observed for the inh+/cefo− sputum samples. On the other hand, the increased levels of ELNE-associated proteins in sputa with above-MIC cefotaxime levels (inh+/cefo+) may reflect a disturbed balance of neutrophil-derived proteases and protease inhibitors. In this respect, it is noteworthy that the sputum composition differs not only from patient to patient, but also over time during treatment in the intensive care unit (ICU), as exemplified by the sputa from patients 020 and 049. This implies that as yet unidentified events during hospitalization may trigger changes in the observed sputum proteome signatures, for instance by activation of the complement system and other innate immune defenses leading to elevated levels of antimicrobial host proteins. The latter observation is important as it provides a tool to measure changes in the lung environment that may be indicative of conditions of the respective patient’s lung. To date, our sample group is too small for definite statements on which of the identified proteins could represent relevant and reliable biomarkers for lung health. However, we consider the fact that time-resolved differences in the sputum proteome of ventilated patients, relating to immunity and antimicrobial activity, can be detected as a breakthrough toward the use of nowadays still discarded sputa as potential indicators for the condition of ICU patients.

## MATERIALS AND METHODS

### Sputum samples.

Sputum samples were derived from a previous study on 53 mechanically ventilated patients admitted to the Neuro Intensive Care Unit of the Department of Critical Care at the University Medical Center Groningen (UMCG). The baseline and clinical characteristics of the 27 patients whose sputa were used in the present study are summarized in Table 1. All investigated sputum samples were previously characterized for antimicrobial activity, microbiota, and cefotaxime concentration (5).

### Sample preparation for shotgun MS.

Flash-frozen sputum aliquots were cryo-fractured, methanol extracted, pelleted, and stored at −20°C until further analysis as previously described (5). Pellets of the cryo-fractured sputum samples were processed for proteome analysis essentially as described previously (56). Briefly, sputum pellets were resuspended in a solution of 8 M urea and 2 M thiourea and subjected to five cycles of freezing in liquid nitrogen and thawing at 37°C for 10 min with vigorous shaking. The resulting samples were further homogenized by three ultrasonication pulses, each of 30 s at 50% power (SonoPuls; Bandelin Electronic, Berlin, Germany). After centrifugation (∼20,000 × *g*, 1 h, 20°C), carried out to remove any remaining insoluble materials, the protein concentration of the resulting supernatant fraction was quantified using a Bradford assay (Bio-Rad, Munich, Germany). For each sample, 4 μg of protein was reduced with dithiothreitol (DTT) (final concentration, 2.5 mM) for 30 min at 37°C and alkylated with iodoacetamide (IAA) (final concentration, 10 mM) for 15 min at 37°C. Digestion with trypsin was done overnight at 37°C in a ratio of 1:25 and stopped by adding acetic acid (final concentration, 1%). The peptide solution was purified using ZipTipμ_C18_ columns (Merck Millipore, Billerica, MA, USA) using decreasing concentrations of acetonitrile (ACN) (80, 50, and 30%) in 1% acetic acid. The resulting samples were frozen at −20°C and subsequently lyophilized.

### Sample measurement by shotgun MS.

Peptides were separated by LC on a nanoAcquity UPLC system (Waters Corporation, USA) coupled to a LTQ-Orbitrap Velos mass spectrometer (Thermo Electron Corporation, Germany) equipped with a nano-electrospray ionization (ESI) source and installed with a Picotip Emitter (New Objective, USA). For LC separation, the digested peptides were first enriched on a nanoAcquity UPLC 2G-V/Mtrap Symmetry C_18_ precolumn (2-cm length, 180-μm inner diameter, and 5-μm particle size; Waters Corporation) and subsequently separated using a NanoAcquity BEH130 C_18_ column (10-cm length, 100-μm inner diameter, 1.7-μm particle; Waters Corporation). The separation was achieved with a gradient of 91 min containing buffer A (0.5% dimethyl sulfoxide [DMSO] in water with 0.1% acetic acid) and buffer B (5.0% DMSO in acetonitrile with 0.1% acetic acid) (gradient, 1 to 5% buffer B in 2 min, 5 to 25% buffer B in 63 min, 25 to 60% buffer B in 25 min, 60 to 99% buffer B in 1 min). The peptides were eluted at a flow rate of 400 nl/min. The eluted peptides were analyzed first by Fourier transform MS (FTMS), operated in positive and profile mode. Next, a MS/MS scan was performed in the data-dependent mode to fragment peptides, and data were acquired in the positive, centroid mode. The MS switched automatically between the Orbitrap-MS and linear trap quadrupole (LTQ) MS/MS acquisition to carry out MS and MS/MS. Survey full scan MS spectra (from *m/z* 325 to 1525) were acquired in the Orbitrap with resolution *R *= 30,000 with a target value of 1 × 10^6^. The method allowed sequential isolation of a maximum of the 20 most intense ions, depending on signal intensity, and were subjected to collision-induced dissociation (CID) fragmentation with an isolation width of 2 Da and a target value of 1 × 10^4^, or with a maximum ion time of 100 ms. Target ions already selected for MS/MS were dynamically excluded for 60 s. General MS conditions were electrospray voltage of 1.6 to 1.7 kV, no sheath and auxiliary gas flow, and a capillary temperature of 300°C. The ion selection threshold was 2,000 counts for MS/MS, activation time of 10 ms, and normalized activation energy of 35%. Only doubly and triply charged ions were triggered for tandem MS analysis.

### Shotgun MS data analysis.

Data were analyzed with Genedata Expressionist software (v.13.0.1) and Mascot (v2.6.2). The raw MS data were processed using two Genedata modules: Refiner MS for data preprocessing, and Analyst for data postprocessing and statistical analyses. Briefly, after noise reduction, LC-MS1 peaks were detected and their properties were calculated (*m/z* and retention time [RT] boundaries, *m/z* and RT center values, intensity). Chromatograms were further aligned based on the RT spectra. Individual peaks were grouped into clusters, and MS/MS data associated with these clusters were annotated with a Mascot MS/MS ions search using a peptide tolerance of 10.0 ppm, a MS/MS tolerance of 0.50 Da, a maximum number of missed cleavages of 2, and the UniProt database for Homo sapiens (20,659 entries). Results were validated by applying a threshold of 1% corrected normalized false discovery rate (FDR). Protein interference was done based on peptide and protein annotations. Redundant proteins were ignored according to Occam’s razor principle, and at least one unique peptide was required for a positive protein identification. Protein intensities were computed using the Hi3 method. Human proteins or derivative peptides with potential to inhibit bacterial growth were identified based on entries in the antimicrobial peptide database (February 2020, https://www.re3data.org/repository/r3d100012901) and/or their functional description in UniProt (12). Protein networks were created with STRING (December 2019, https://string-db.org/).

### Heterologous expression and purification of recombinant pneumococcal antigens.

Recombinant antigens used for xMAP technology, bead-based analyses are listed in Table S1 in the supplemental material. Target genes were amplified by PCR using chromosomal template DNA from S. pneumoniae and the primer pairs listed in Table S1. The amplified genes were cloned in appropriate expression vectors, and the resulting constructs were used to transform Escherichia coli M15 (for pQE30-based constructs) or BL21 (for all other constructs). For protein production, E. coli was cultured at 30°C in lysogeny broth supplemented with appropriate antibiotics. When an optical density at 600 nm (OD_600_) of 0.6 to 0.8 was reached, protein expression was induced with 1 mM anhydrotetracycline (for pneumolysin) or 1 mM isopropyl-β-d-1-thiogalactopyranoside (for all other proteins) for 3 h at 30°C. Recombinant proteins were purified by affinity chromatography using the methods indicated in Table S1, followed by dialysis (12- to 14-kDa molecular weight cutoff) against phosphate-buffered saline (PBS) (pH 7.4). Purity of the recombinant proteins was verified by sodium dodecyl sulfate-polyacrylamide gel electrophoresis (SDS-PAGE) and mass spectrometry.

10.1128/mSystems.00702-20.6 TABLE S1 Overview of recombinantly expressed and purified pneumococcal antigens. The purification methods are indicated by the following superscript letters as follows: ^a^, HisTrap Ni-NTA HP 1-ml column with ÄKTApurifier liquid chromatography system (GE Healthcare GmbH); ^b^, DEAE-cellulose column; ^c^, DEAE-cellulose column plus HisTrap Ni-NTA HP 1-ml column with ÄKTApurifier liquid chromatography system (GE Healthcare GmbH); ^d^, HisTrap Ni-NTA HP 1-ml column with ÄKTApurifier liquid chromatography system (GE Healthcare GmbH) plus gel filtration Superdex 200; ^e^, StrepTactin Sepharose High Performance (GE Healthcare GmbH). The asterisk in the Tag column indicates CHiC, a choline-binding histidine combination tag. Download Table S1, DOCX file, 0.2 MB. Copyright © 2021 Seinen et al. 2021 Seinen et al. https://creativecommons.org/licenses/by/4.0/ This content is distributed under the terms of the Creative Commons Attribution 4.0 International license.

### Immunoproteome analysis using the Luminex xMAP technology.

Recombinant proteins were immobilized on xMAP MagPlex beads using stocks of 100 μg/ml. The sputum titers of total IgG directed against 55 S. pneumoniae antigens (Table S1) were quantified using the Luminex xMAP technology, and the recorded data were analyzed with the xMAPr app as previously described (57). To adapt the protocol for analysis of IgG titers in sputa, the samples were centrifuged (∼3,000 × *g*, 5 min, room temperature), and supernatant aliquots of 15 μl were used for further processing. Of note, most sputum samples separated into pellet and supernatant fractions after centrifugation, but for some sputa, even an additional centrifugation at a higher speed (5,000 × *g*) could not result in a cleared supernatant fraction. Nevertheless, these samples were also included in the analysis, and they have been marked in Table S2. Due to a lower expected IgG titer in sputum compared to the levels measured in human serum, the sputum samples were fourfold serially diluted (1:20, 1:80, 1:320, 1:1,280, 1:5,120, 1:20,480, and 1:81,920). Sputum samples 005-1 (inh−) and 036-1 (inh+/cefo+) had to be excluded from further analyses, due to missing values. Plots were generated using R (v.3.6.1) with the Tidyverse package (v.1.3.0.) (58).

### Statistics.

For the proteomics analyses, samples were first grouped into inhibiting and noninhibiting sputa (or proteins thereof), and the respective MS data were postprocessed with Genedata Analyst (v.13.0.1) or GraphPad Prism (v.5). The statistical analyses included partial least squares (PLS) analyses, *t* tests, and correlation tests. The relative abundance of proteins was considered significant with a *P* value of ≤0.05 and an effect size (ES) of ≥1.5 (59). It should be noted here that, in the case of sputum collection from mechanically ventilated patients, it is not possible to collect biological replicate samples at one time point, both for ethical and practical reasons. Consequently, we performed one technical replicate measurement for each sputum sample. With respect to our tandem MS measurements, using the data-dependent acquisition (DDA) for data collection, the coefficients of variation (CVs) cover both the technical and biological variances, which are usually in the range of 5 to 20%. To verify this, we have calculated the CVs in percent per PLS group, which yielded a normal distribution. Importantly, the vast majority of calculated protein CVs were below 20% variation per group, indicating that one technical replicate measurement was sufficient. For the final data analysis, we combined the *P* values (*P* ≤ 0.05) and the alterations in effect size (ES ≥1.5), in order to ensure that the significance threshold would not overlap with the mass spectrometry variation. Importantly, this approach is fully in line with the recommendation by Pascovici et al. (59), who compared multiple testing corrections with *P* value-ES combinations in quantitative proteomics analyses. Furthermore, we have performed a linear discriminant analysis (LDA) based on the three groups and compared the obtained results with our PLS analysis. However, the PLS separated the different groups much better than the LDA (data not shown), which probably relates to our high-dimensional MS feature data for which an LDA is not optimal (59–61).

Gene Ontology analysis was performed within an R statistical programming environment utilizing UniProt web services (uniprot.ws package, database release 10/2019) for annotation. Statistical overrepresentation of GO terms within protein groups of interest was verified using the Fisher’s exact test against the data set defined in this study.

For statistical analysis of the data recorded by Luminex xMAP technology, the Wilcoxon rank sum test was used. An adjusted *P* value of ≤0.05 was considered significant (Benjamini and Hochberg’s multiple testing correction), and a fold change of 2.0 was used as the cutoff value for the respective volcano plot.

### Study approval.

The collection and analysis of sputa from mechanically ventilated ICU patients were approved by the Medical Ethical Committee of the UMCG (research project number 2014.309), which decided that informed consent was not necessary since all patients admitted to the UMCG are informed that their data and (diagnostic) waste materials can be used for scientific research. All patient data and samples were collected and processed anonymously with adherence to the Helsinki Guidelines.

### Accession number(s).

The raw MS data were uploaded in MassIVE (https://massive.ucsd.edu) under the accession number MSV000085288 (https://doi.org/10.25345/C5871S).
