# Supplementary figures and images for: Sputum Proteome Signatures of Mechanically Ventilated Intensive Care Unit Patients Distinguish Samples with or without Anti-pneumococcal Activity
Source: mSystems. 2021 Mar 2;6(2):e00702-20. doi: 10.1128/mSystems.00702-20 (PMC8546979; doi:10.1128/mSystems.00702-20)

**
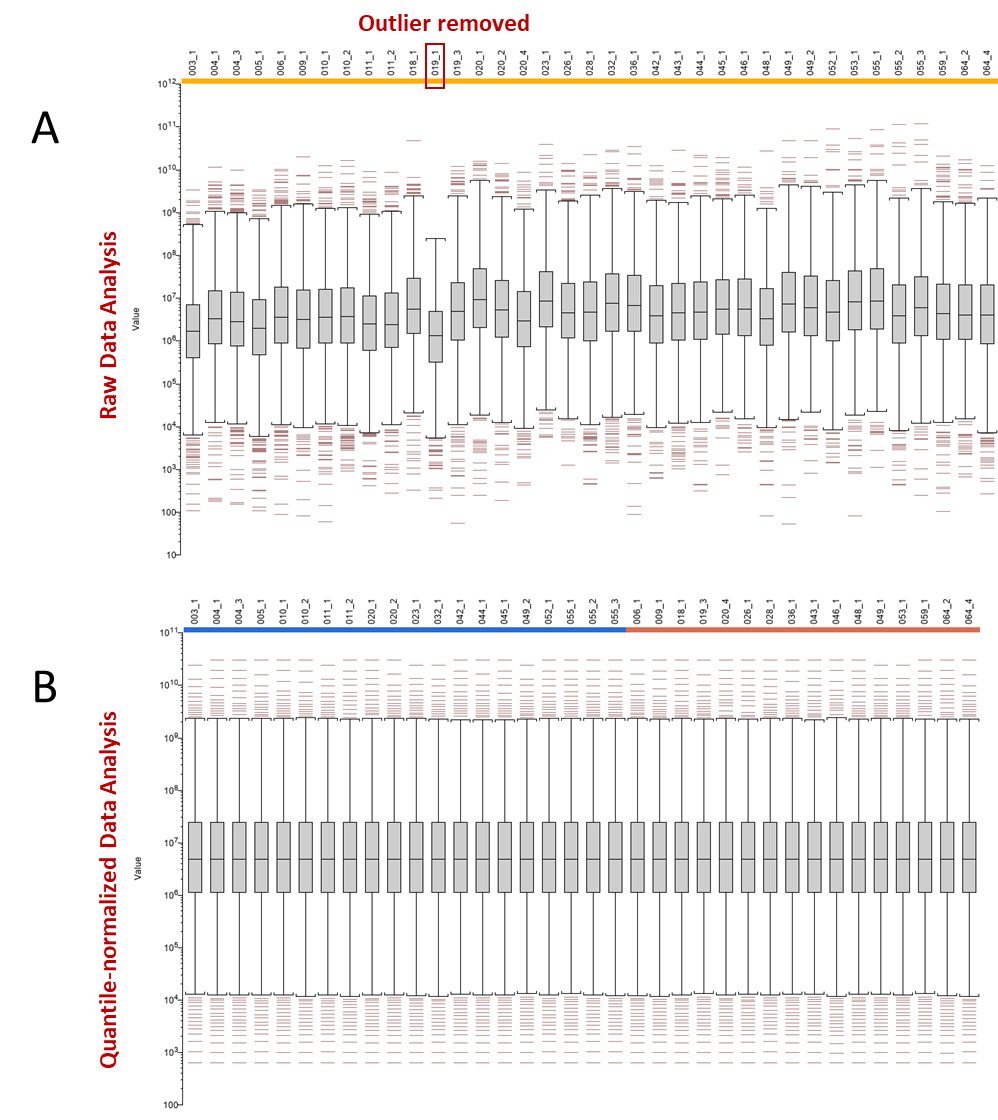
**

Supplement: FIG S1 [file msystems.00702-20-sf001.docx]

**
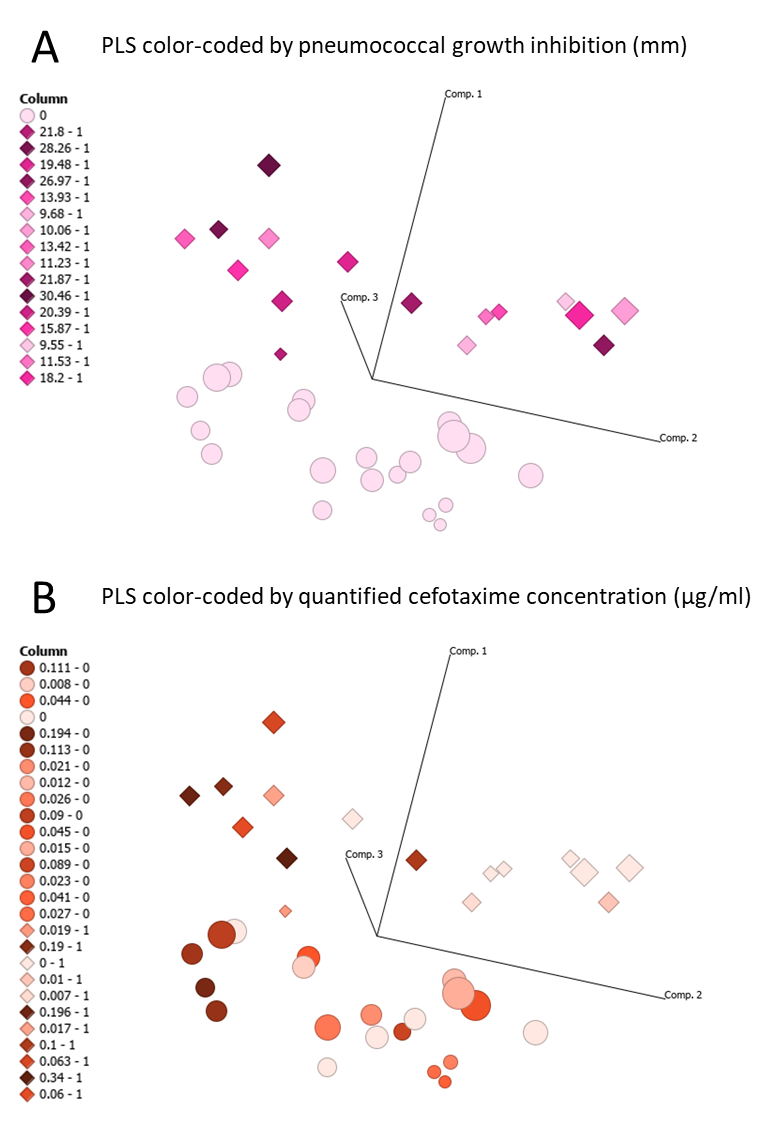
**

Supplement: FIG S3 [file msystems.00702-20-sf003.docx]
