# Supplementary material for: Sputum Proteome Signatures of Mechanically Ventilated Intensive Care Unit Patients Distinguish Samples with or without Anti-pneumococcal Activity
Source: mSystems. 2021 Mar 2;6(2):e00702-20. doi: 10.1128/mSystems.00702-20 (PMC8546979; doi:10.1128/mSystems.00702-20)
Supplement: TABLE S2 [file msystems.00702-20-st002.docx]

**Table S2.** Overview of included patients and sputum samples.

| Patient | Sample |  | Pneumococcal inhibition in mm | [cefotaxime] in µg/ml |
| --- | --- | --- | --- | --- |
| 003 | 003-1^#^ |  | 0.00 | 0.111 |
| 004 | 004-1 |  | 0.00 | 0.008 |
|  | 004-3^#^ |  | 0.00 | 0.044 |
| 005 | 005-1^#^ |  | 0.00 | ND* |
| 006 | 006-1 |  | 21.80 | 0.019 |
| 009 | 009-1 |  | 28.26 | 0.190 |
| 010 | 010-1 |  | 0.00 | 0.194 |
|  | 010-2 |  | 0.00 | 0.113 |
| 011 | 011-1 |  | 0.00 | ND** |
|  | 011-2 |  | 0.00 | 0.021 |
| 018 | 018-1^#^ |  | 19.48 | ND* |
| 019 | 019-1 |  | 0.00 | ND** |
|  | 019-3^#^ |  | 26.97 | 0.010 |
| 020 | 020-1^#^ |  | 0.00 | 0.012 |
|  | 020-2^#^ |  | 0.00 | 0.026 |
|  | 020-4 |  | 13.93 | ND** |
| 023 | 023-1 |  | 0.00 | 0.090 |
| 026 | 026-1 |  | 9.68 | 0.007 |
| 028 | 028-1 |  | 10.06 | ND** |
| 032 | 032-1 |  | 0.00 | 0.045 |
| 036 | 036-1 |  | 13.42 | 0.196 |
| 042 | 042-1 |  | 0.00 | ND** |
| 043 | 043-1 |  | 11.23 | 0.017 |
| 044 | 044-1 |  | 0.00 | ND** |
| 045 | 045-1^#^ |  | 0.00 | ND** |
| 046 | 046-1 |  | 21.87 | 0.100 |
| 048 | 048-1 |  | 30.46 | 0.063 |
| 049 | 049-1 |  | 20.39 | 0.340 |
|  | 049-2 |  | 0.00 | 0.015 |
| 052 | 052-1 |  | 0.00 | 0.089 |
| 053 | 053-1 |  | 15.87 | 0.060 |
| 055 | 055-1 |  | 0.00 | 0.023 |
|  | 055-2^#^ |  | 0.00 | 0.041 |
|  | 055-3^#^ |  | 0.00 | 0.027 |
| 059 | 059-1 |  | 9.55 | ND* |
| 064 | 064-2 |  | 11.53 | ND** |
|  | 064-4 |  | 18.20 | ND** |
