# Supplementary material for: Sputum Proteome Signatures of Mechanically Ventilated Intensive Care Unit Patients Distinguish Samples with or without Anti-pneumococcal Activity
Source: mSystems. 2021 Mar 2;6(2):e00702-20. doi: 10.1128/mSystems.00702-20 (PMC8546979; doi:10.1128/mSystems.00702-20)
Supplement: FIG S5 [file msystems.00702-20-sf005.pdf]

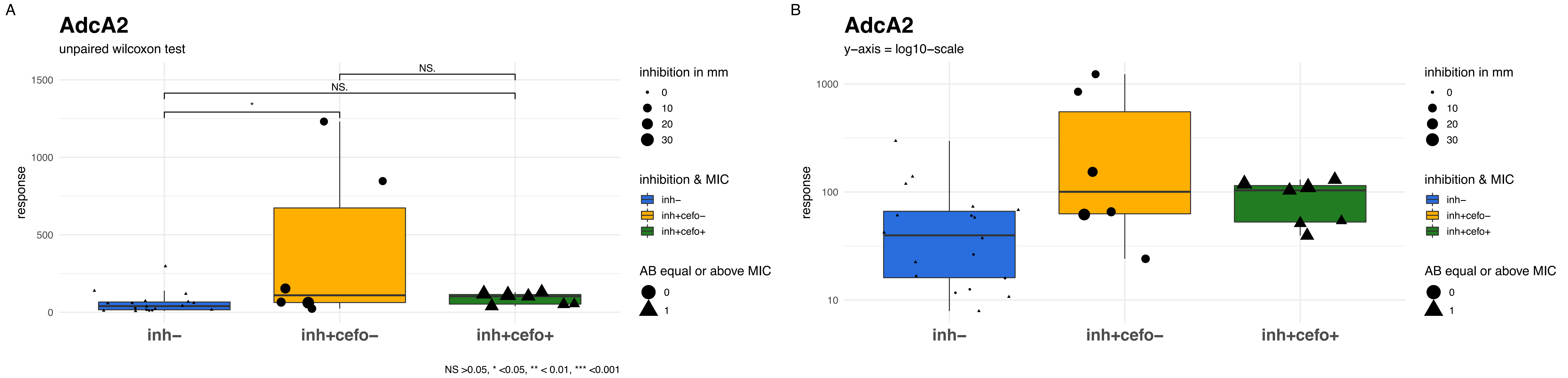

A
AdcA2
unpaired wilcoxon test
NS.
NS.
*
1500
1000
500
0
response
inh−
inh+cefo−
inh+cefo+
NS >0.05, * <0.05, ** < 0.01, *** <0.001
inhibition in mm
0
10
20
30
inhibition & MIC
inh−
inh+cefo−
inh+cefo+
AB equal or above MIC
0
1
B
AdcA2
y−axis = log10−scale
1000
100
10
response
inh−
inh+cefo−
inh+cefo+
inhibition in mm
0
10
20
30
inhibition & MIC
inh−
inh+cefo−
inh+cefo+
AB equal or above MIC
0
1

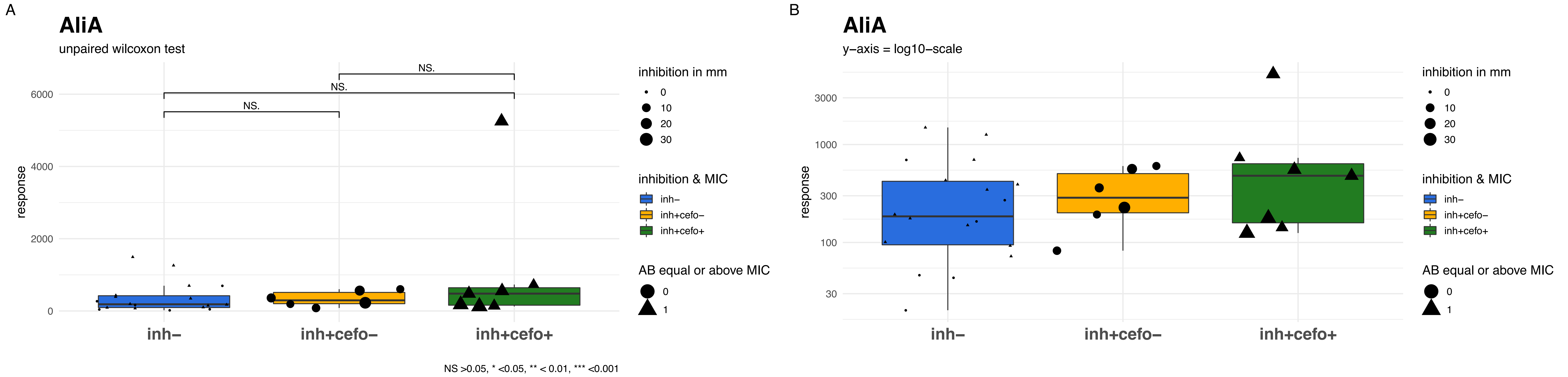

A
AliA
unpaired wilcoxon test
NS.
NS.
NS.
6000
4000
2000
0
response
inh−
inh+cefo−
inh+cefo+
inhibition in mm
0
10
20
30
inhibition & MIC
inh−
inh+cefo−
inh+cefo+
AB equal or above MIC
0
1
NS >0.05, * <0.05, ** < 0.01, *** <0.001
B
AliA
y−axis = log10−scale
3000
1000
300
100
30
response
inh−
inh+cefo−
inh+cefo+
inhibition in mm
0
10
20
30
inhibition & MIC
inh−
inh+cefo−
inh+cefo+
AB equal or above MIC
0
1

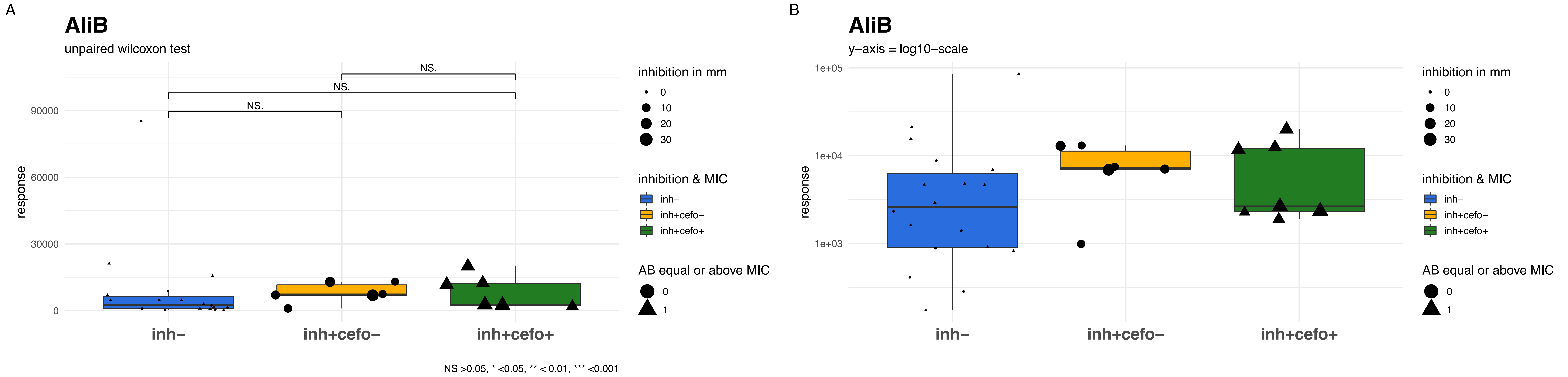
A
AliB
unpaired wilcoxon test
NS.
NS.
NS.
90000
60000
30000
0
response
inh−
inh+cefo−
inh+cefo+
inhibition in mm
0
10
20
30
inhibition & MIC
inh−
inh+cefo−
inh+cefo+
AB equal or above MIC
0
1
NS >0.05, * <0.05, ** < 0.01, *** <0.001
B
AliB
y−axis = log10−scale
1e+05
1e+04
1e+03
response
inh−
inh+cefo−
inh+cefo+
inhibition in mm
0
10
20
30
inhibition & MIC
inh−
inh+cefo−
inh+cefo+
AB equal or above MIC
0
1

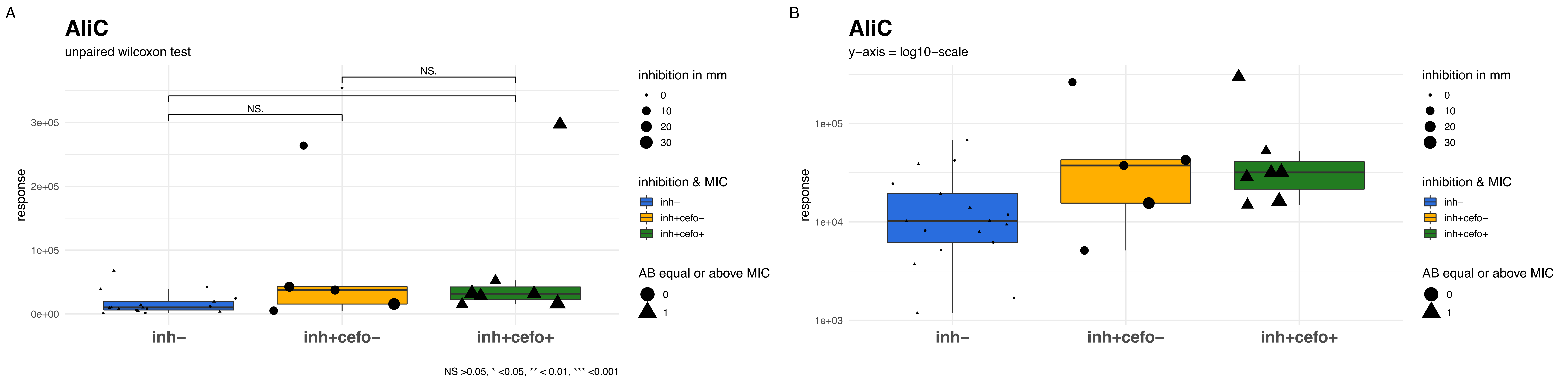

A
AliC
unpaired wilcoxon test
NS.
*
NS.
3e+05
2e+05
1e+05
0e+00
response
inh−
inh+cefo−
inh+cefo+
inhibition in mm
0
10
20
30
inhibition & MIC
inh−
inh+cefo−
inh+cefo+
AB equal or above MIC
0
1
NS >0.05, * <0.05, ** < 0.01, *** <0.001
B
AliC
y−axis = log10−scale
1e+05
1e+04
1e+03
response
inh−
inh+cefo−
inh+cefo+
inhibition in mm
0
10
20
30
inhibition & MIC
inh−
inh+cefo−
inh+cefo+
AB equal or above MIC
0
1

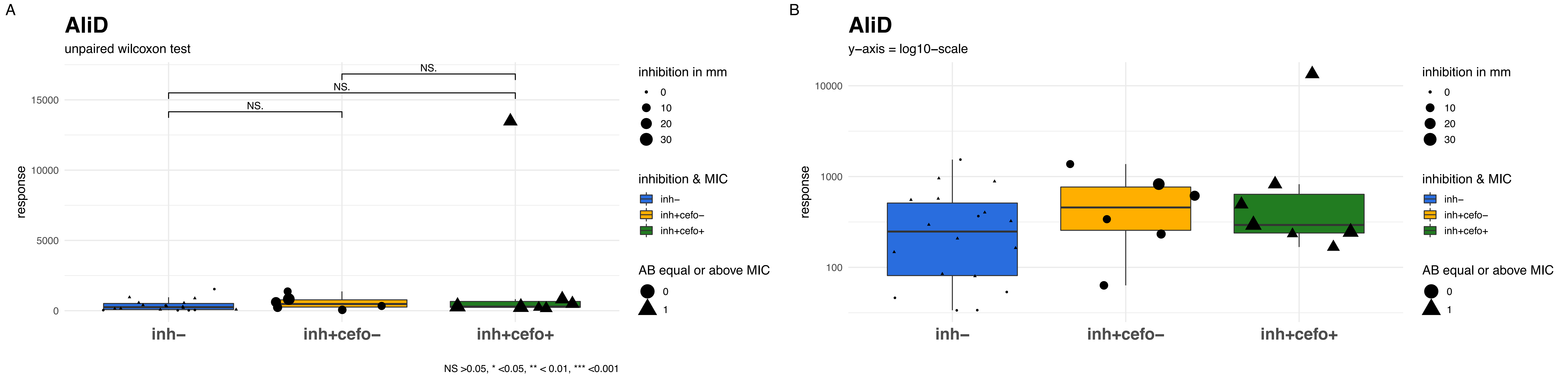

A
AliD
unpaired wilcoxon test
NS.
NS.
NS.
response
15000
10000
5000
0
inh−
inh+cefo−
inh+cefo+
inhibition in mm
0
10
20
30
inhibition & MIC
inh−
inh+cefo−
inh+cefo+
AB equal or above MIC
0
1
NS >0.05, * <0.05, ** < 0.01, *** <0.001
B
AliD
y−axis = log10−scale
response
10000
1000
100
inh−
inh+cefo−
inh+cefo+
inhibition in mm
0
10
20
30
inhibition & MIC
inh−
inh+cefo−
inh+cefo+
AB equal or above MIC
0
1

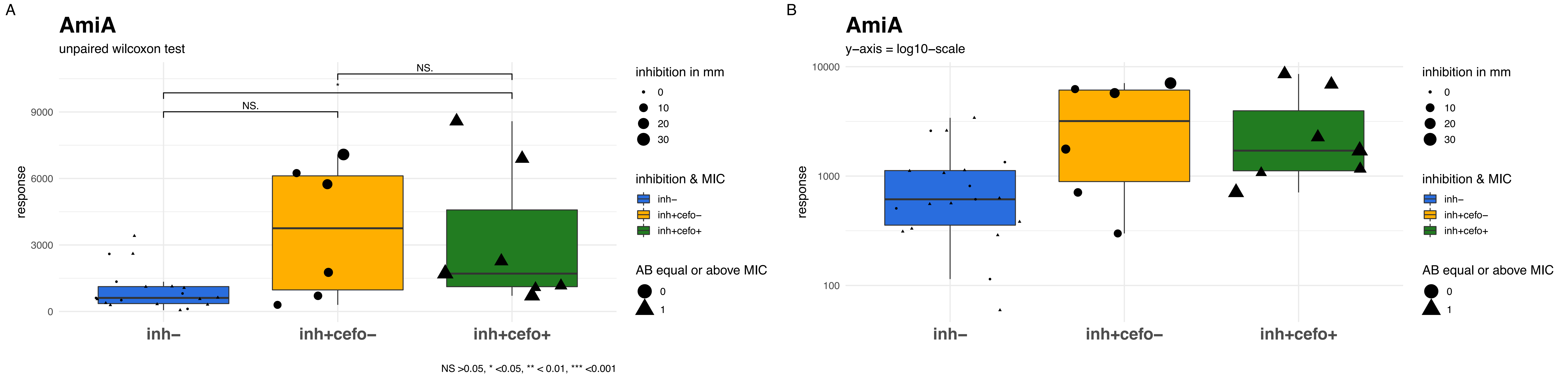
A
AmiA
unpaired wilcoxon test
NS.
*
NS.
9000
6000
3000
0
response
inh−
inh+cefo−
inh+cefo+
inhibition in mm
0
10
20
30
inhibition & MIC
inh−
inh+cefo−
inh+cefo+
AB equal or above MIC
0
1
NS >0.05, * <0.05, ** < 0.01, *** <0.001
B
AmiA
y−axis = log10−scale
10000
1000
100
response
inh−
inh+cefo−
inh+cefo+
inhibition in mm
0
10
20
30
inhibition & MIC
inh−
inh+cefo−
inh+cefo+
AB equal or above MIC
0
1

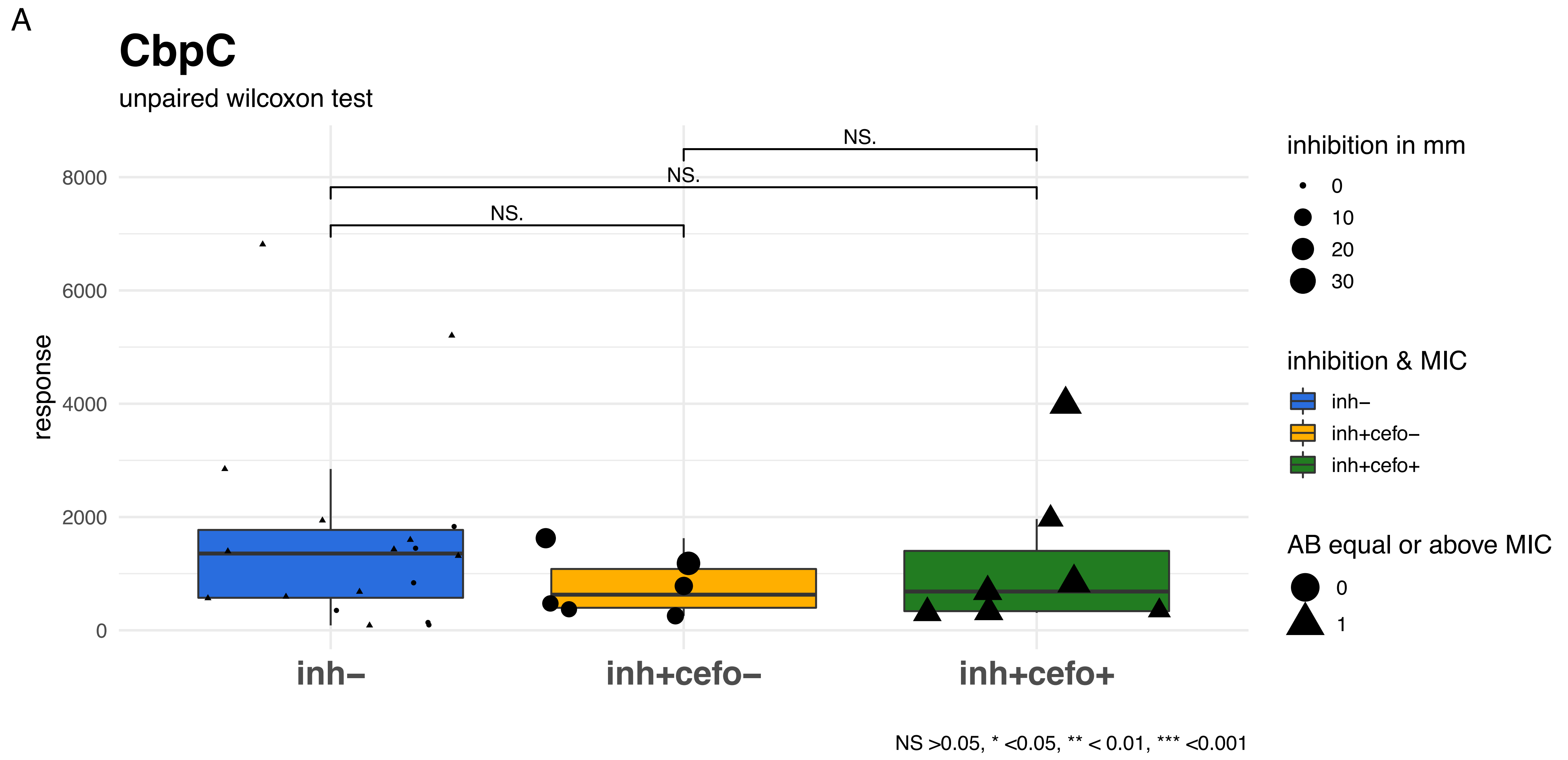

A
CbpC
unpaired wilcoxon test
NS.
NS.
NS.
8000
6000
4000
2000
0
response
inh−
inh+cefo−
inh+cefo+
inhibition in mm
0
10
20
30
inhibition & MIC
inh−
inh+cefo−
inh+cefo+
AB equal or above MIC
0
1
NS >0.05, * <0.05, ** < 0.01, *** <0.001

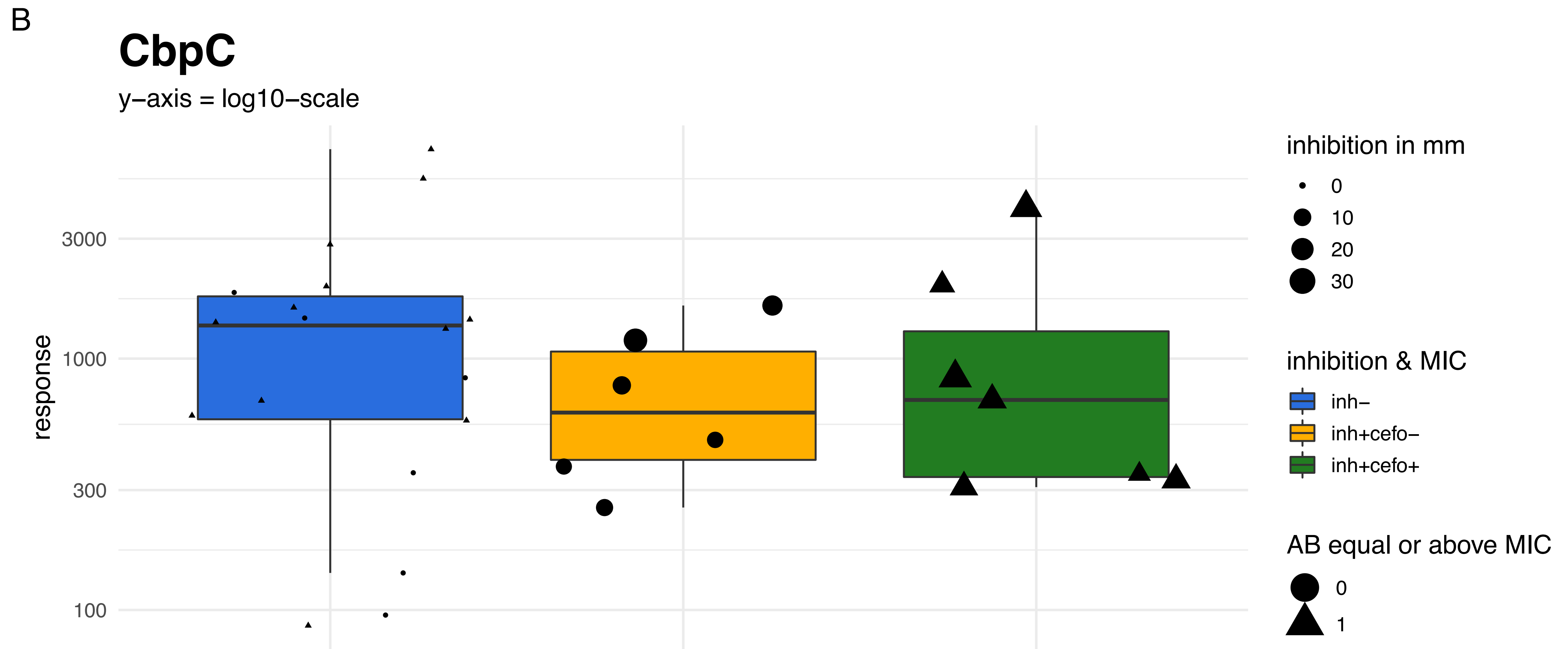

B
CbpC
y−axis = log10−scale
3000
1000
300
100
response
inh−
inh+cefo−
inh+cefo+
inhibition in mm
0
10
20
30
inhibition & MIC
inh−
inh+cefo−
inh+cefo+
AB equal or above MIC
0
1

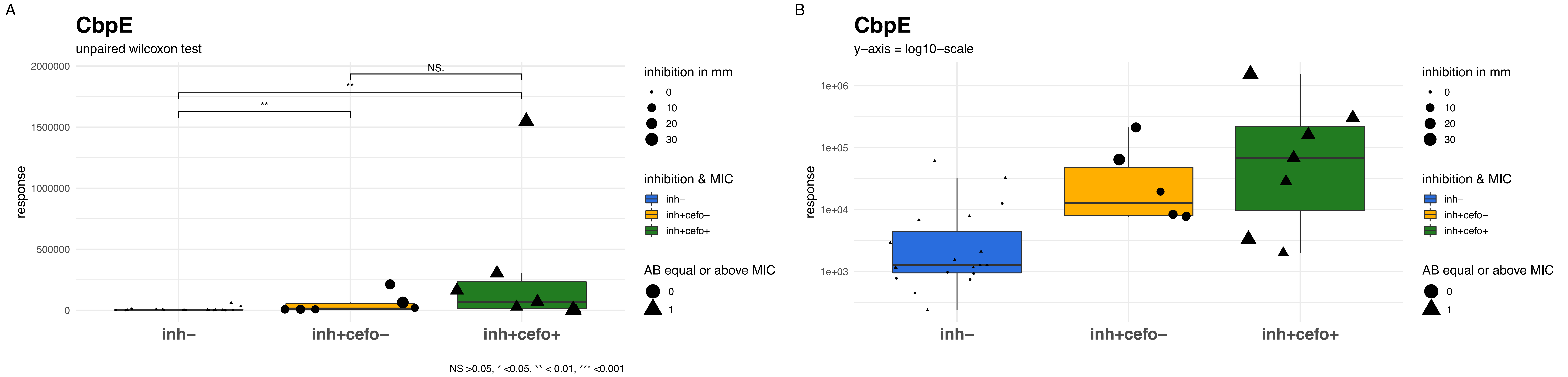

A
CbpE
unpaired wilcoxon test
2000000
1500000
1000000
500000
0
response
NS.
**
**
inh−
inh+cefo−
inh+cefo+
inhibition in mm
0
10
20
30
inhibition & MIC
inh−
inh+cefo−
inh+cefo+
AB equal or above MIC
0
1
NS >0.05, * <0.05, ** < 0.01, *** <0.001
B
CbpE
y−axis = log10−scale
1e+06
1e+05
1e+04
1e+03
response
inh−
inh+cefo−
inh+cefo+
inhibition in mm
0
10
20
30
inhibition & MIC
inh−
inh+cefo−
inh+cefo+
AB equal or above MIC
0
1

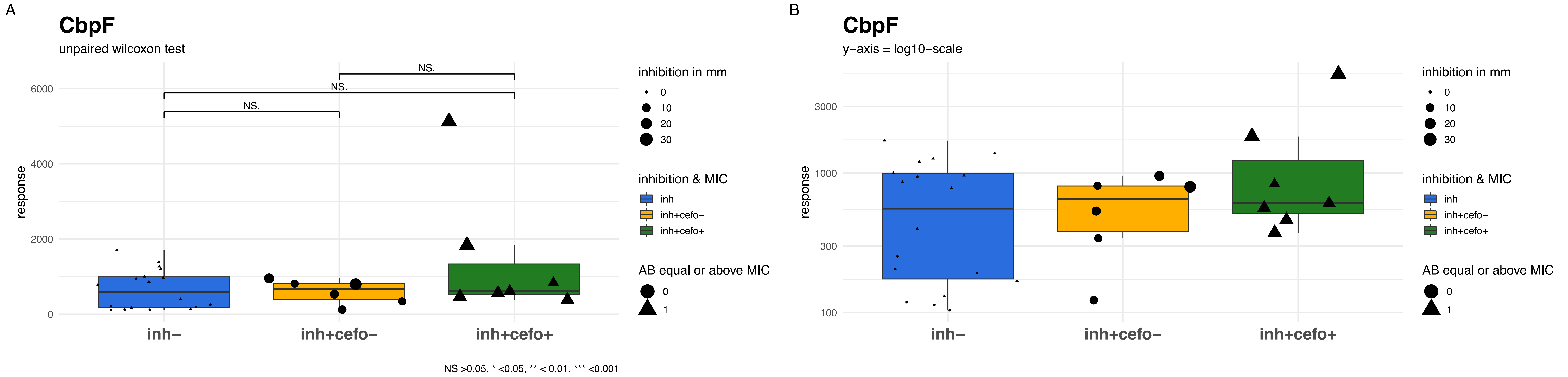
A
CbpF
unpaired wilcoxon test
NS.
NS.
NS.
6000
4000
2000
0
response
inh−
inh+cefo−
inh+cefo+
NS >0.05, * <0.05, ** < 0.01, *** <0.001
inhibition in mm
0
10
20
30
inhibition & MIC
inh−
inh+cefo−
inh+cefo+
AB equal or above MIC
0
1
B
CbpF
y−axis = log10−scale
3000
1000
300
100
response
inh−
inh+cefo−
inh+cefo+
inhibition in mm
0
10
20
30
inhibition & MIC
inh−
inh+cefo−
inh+cefo+
AB equal or above MIC
0
1

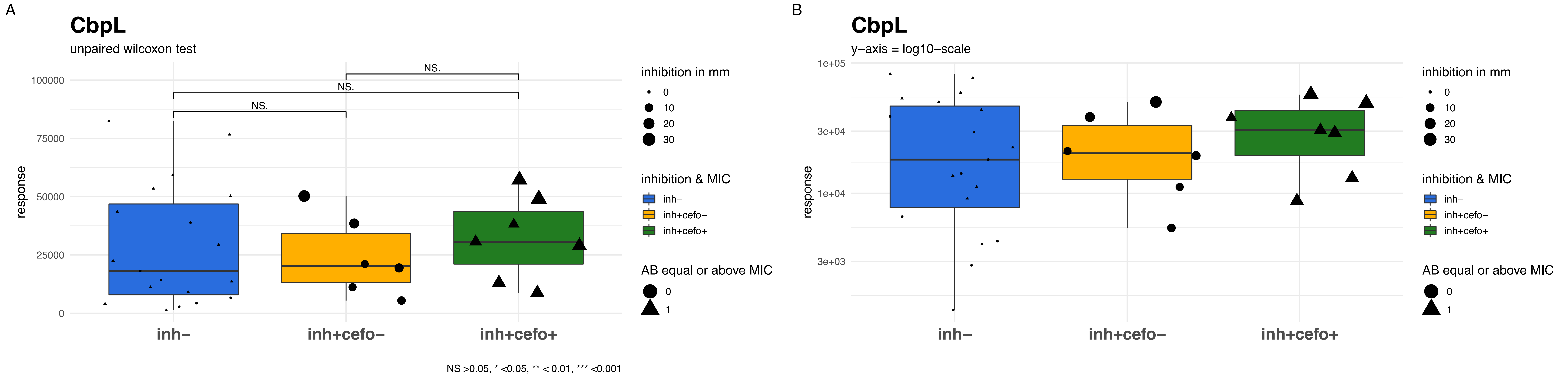
A
CbpL
unpaired wilcoxon test
NS.
NS.
NS.
100000
75000
50000
25000
0
response
inh−
inh+cefo−
inh+cefo+
inhibition in mm
0
10
20
30
inhibition & MIC
inh−
inh+cefo−
inh+cefo+
AB equal or above MIC
0
1
NS >0.05, * <0.05, ** < 0.01, *** <0.001
B
CbpL
y−axis = log10−scale
1e+05
3e+04
1e+04
3e+03
response
inh−
inh+cefo−
inh+cefo+
inhibition in mm
0
10
20
30
inhibition & MIC
inh−
inh+cefo−
inh+cefo+
AB equal or above MIC
0
1

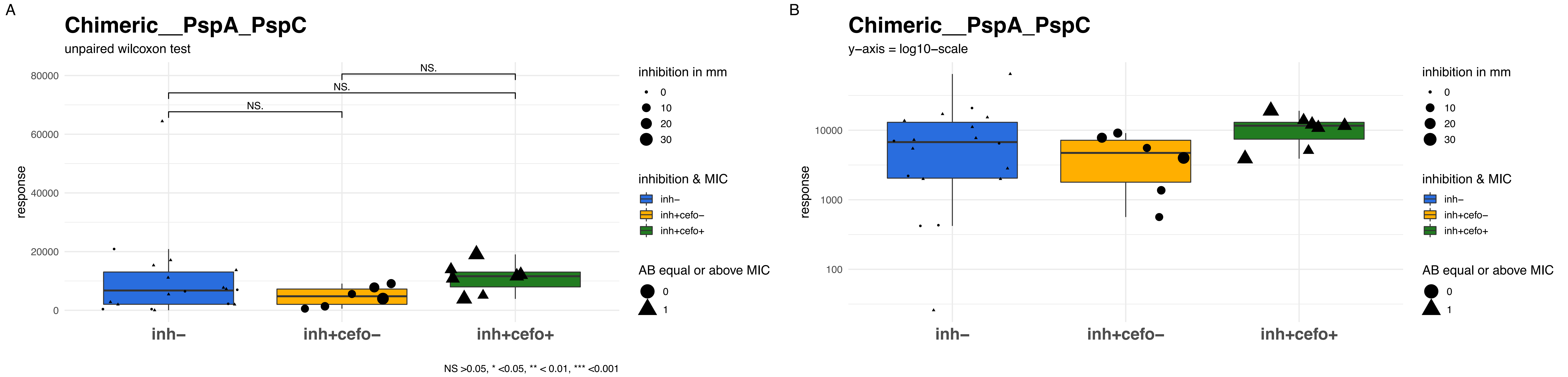

A
Chimeric__PspA_PspC
unpaired wilcoxon test
NS.
NS.
NS.
80000
60000
40000
20000
0
response
inh−
inh+cefo−
inh+cefo+
inhibition in mm
0
10
20
30
inhibition & MIC
inh−
inh+cefo−
inh+cefo+
AB equal or above MIC
0
1
NS >0.05, * <0.05, ** < 0.01, *** <0.001
B
Chimeric__PspA_PspC
y−axis = log10−scale
10000
1000
100
response
inh−
inh+cefo−
inh+cefo+
inhibition in mm
0
10
20
30
inhibition & MIC
inh−
inh+cefo−
inh+cefo+
AB equal or above MIC
0
1

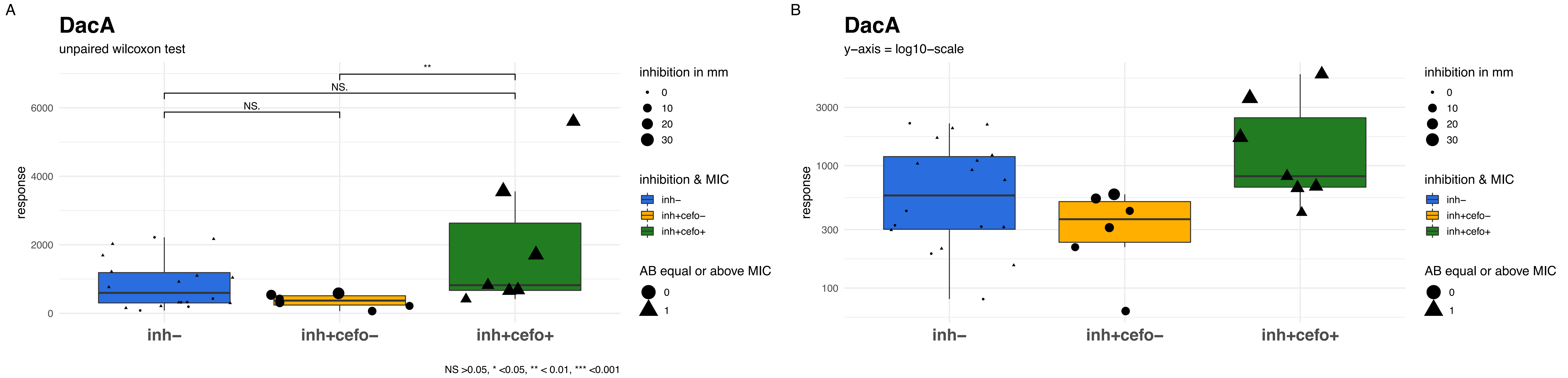
A
DacA
unpaired wilcoxon test
**
NS.
NS.
6000
4000
2000
0
response
inh−
inh+cefo−
inh+cefo+
NS >0.05, * <0.05, ** < 0.01, *** <0.001
inhibition in mm
0
10
20
30
inhibition & MIC
inh−
inh+cefo−
inh+cefo+
AB equal or above MIC
0
1
B
DacA
y−axis = log10−scale
3000
1000
300
100
response
inh−
inh+cefo−
inh+cefo+
inhibition in mm
0
10
20
30
inhibition & MIC
inh−
inh+cefo−
inh+cefo+
AB equal or above MIC
0
1

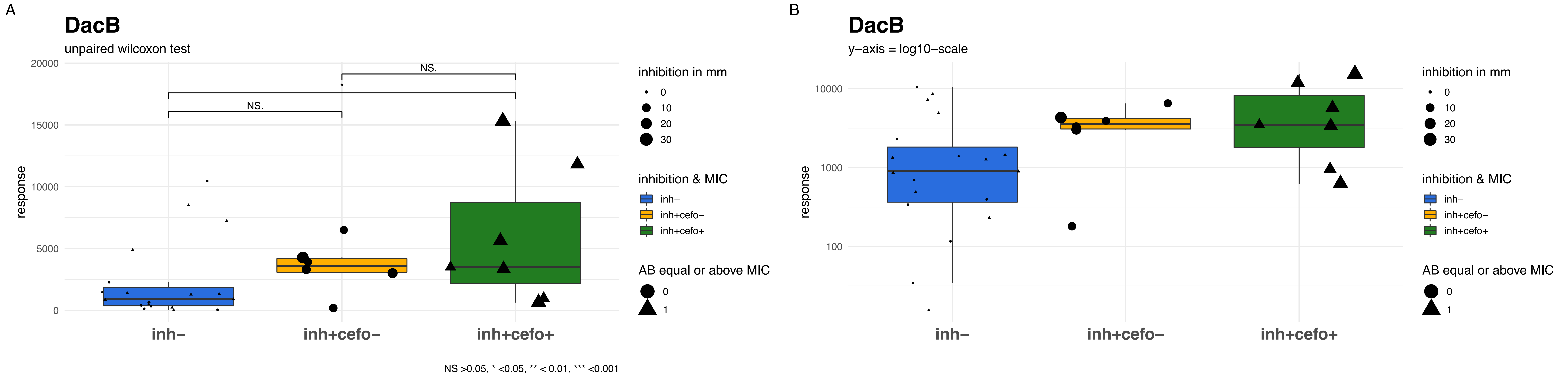
A
DacB
unpaired wilcoxon test
NS.
*
NS.
20000
15000
10000
5000
0
response
inh−
inh+cefo−
inh+cefo+
inhibition in mm
0
10
20
30
inhibition & MIC
inh−
inh+cefo−
inh+cefo+
AB equal or above MIC
0
1
NS >0.05, * <0.05, ** < 0.01, *** <0.001
B
DacB
y−axis = log10−scale
10000
1000
100
response
inh−
inh+cefo−
inh+cefo+
inhibition in mm
0
10
20
30
inhibition & MIC
inh−
inh+cefo−
inh+cefo+
AB equal or above MIC
0
1

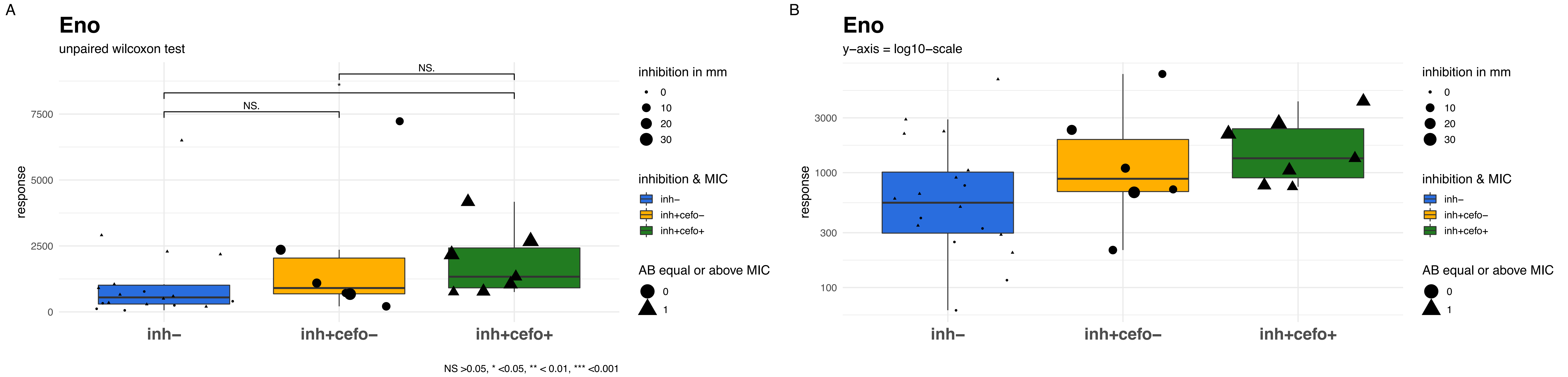
A
Eno
unpaired wilcoxon test
NS.
*
NS.
7500
5000
2500
0
response
inh−
inh+cefo−
inh+cefo+
NS >0.05, * <0.05, ** < 0.01, *** <0.001
inhibition in mm
0
10
20
30
inhibition & MIC
inh−
inh+cefo−
inh+cefo+
AB equal or above MIC
0
1
B
Eno
y−axis = log10−scale
3000
1000
300
100
response
inh−
inh+cefo−
inh+cefo+
inhibition in mm
0
10
20
30
inhibition & MIC
inh−
inh+cefo−
inh+cefo+
AB equal or above MIC
0
1

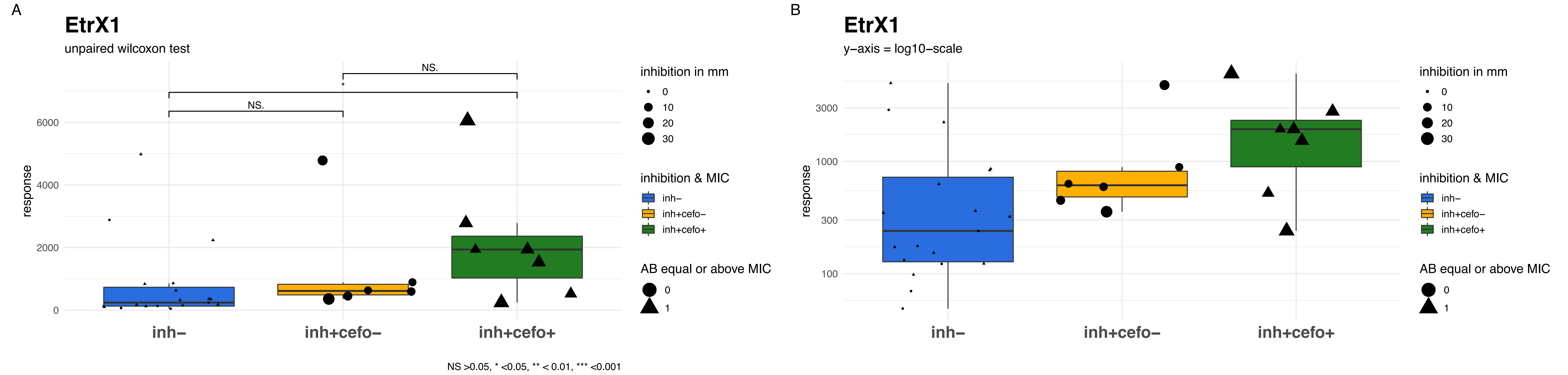
A
EtrX1
unpaired wilcoxon test
NS.
NS.
*
response
6000
4000
2000
0
inh−
inh+cefo−
inh+cefo+
inhibition in mm
0
10
20
30
inhibition & MIC
inh−
inh+cefo−
inh+cefo+
AB equal or above MIC
0
1
NS >0.05, * <0.05, ** < 0.01, *** <0.001
B
EtrX1
y−axis = log10−scale
response
3000
1000
300
100
inh−
inh+cefo−
inh+cefo+
inhibition in mm
0
10
20
30
inhibition & MIC
inh−
inh+cefo−
inh+cefo+
AB equal or above MIC
0
1

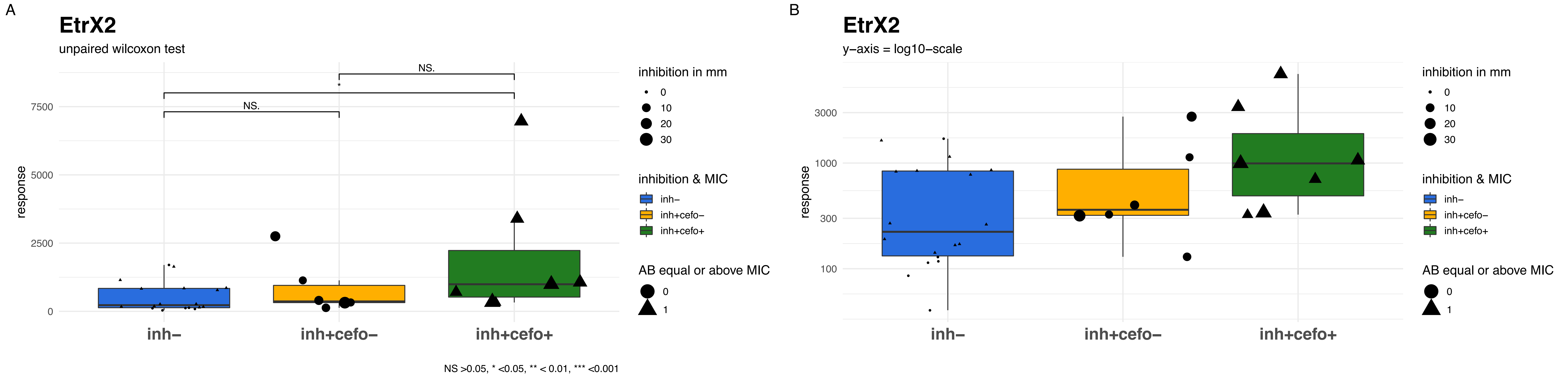
A
EtrX2
unpaired wilcoxon test
NS.
*
NS.
7500
5000
2500
0
response
inh−
inh+cefo−
inh+cefo+
inhibition in mm
0
10
20
30
inhibition & MIC
inh−
inh+cefo−
inh+cefo+
AB equal or above MIC
0
1
NS >0.05, * <0.05, ** < 0.01, *** <0.001
B
EtrX2
y−axis = log10−scale
3000
1000
300
100
response
inh−
inh+cefo−
inh+cefo+
inhibition in mm
0
10
20
30
inhibition & MIC
inh−
inh+cefo−
inh+cefo+
AB equal or above MIC
0
1

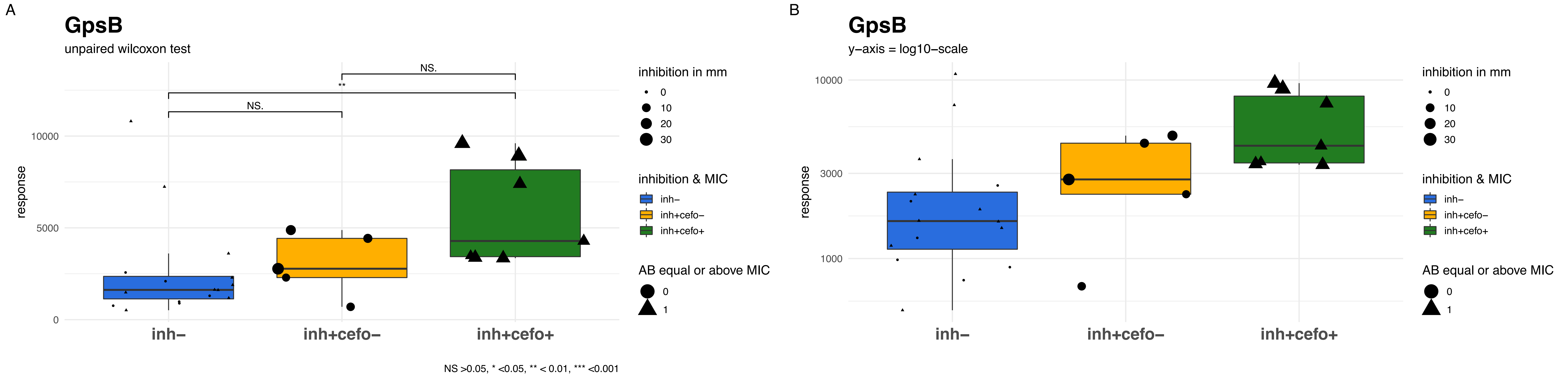
A
GpsB
unpaired wilcoxon test
NS.
**
NS.
10000
5000
0
response
inh−
inh+cefo−
inh+cefo+
NS >0.05, * <0.05, ** < 0.01, *** <0.001
inhibition in mm
0
10
20
30
inhibition & MIC
inh−
inh+cefo−
inh+cefo+
AB equal or above MIC
0
1
B
GpsB
y−axis = log10−scale
10000
3000
1000
response
inh−
inh+cefo−
inh+cefo+
inhibition in mm
0
10
20
30
inhibition & MIC
inh−
inh+cefo−
inh+cefo+
AB equal or above MIC
0
1

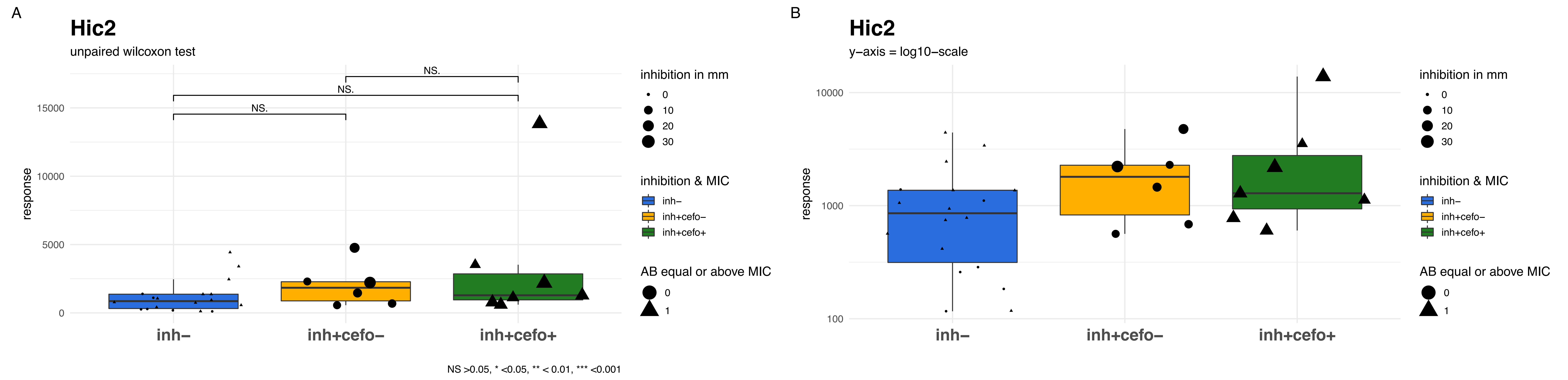
A
Hic2
unpaired wilcoxon test
NS.
NS.
NS.
response
15000
10000
5000
0
inh−
inh+cefo−
inh+cefo+
NS >0.05, * <0.05, ** < 0.01, *** <0.001
inhibition in mm
0
10
20
30
inhibition & MIC
inh−
inh+cefo−
inh+cefo+
AB equal or above MIC
0
1
B
Hic2
y−axis = log10−scale
response
10000
1000
100
inh−
inh+cefo−
inh+cefo+
inhibition in mm
0
10
20
30
inhibition & MIC
inh−
inh+cefo−
inh+cefo+
AB equal or above MIC
0
1

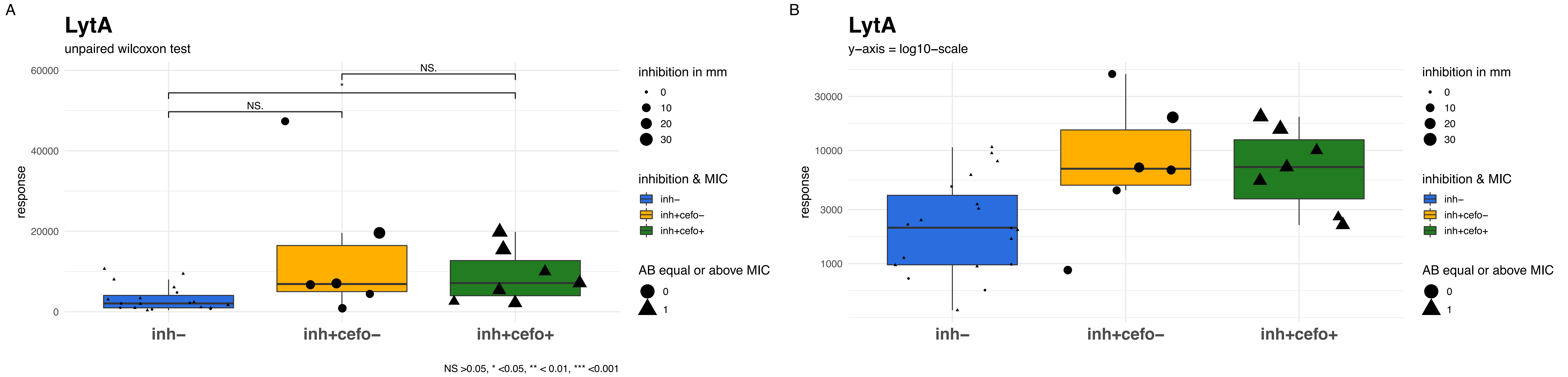

A
LytA
unpaired wilcoxon test
60000
40000
20000
0
response
NS.
*
NS.
inh−
inh+cefo−
inh+cefo+
inhibition in mm
0
10
20
30
inhibition & MIC
inh−
inh+cefo−
inh+cefo+
AB equal or above MIC
0
1
NS >0.05, * <0.05, ** < 0.01, *** <0.001
B
LytA
y−axis = log10−scale
30000
10000
3000
1000
response
inh−
inh+cefo−
inh+cefo+
inhibition in mm
0
10
20
30
inhibition & MIC
inh−
inh+cefo−
inh+cefo+
AB equal or above MIC
0
1

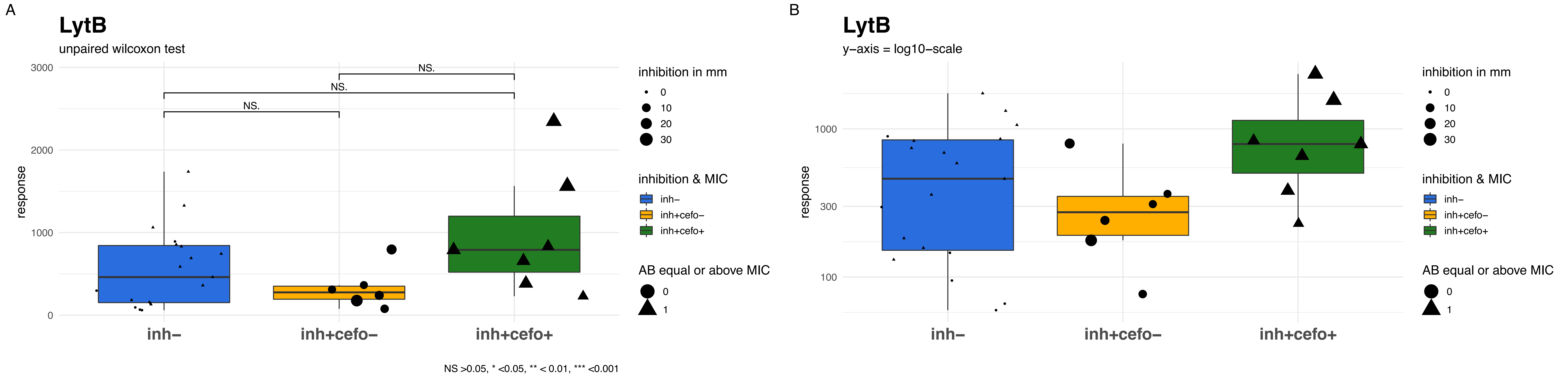

A
LytB
unpaired wilcoxon test
3000
2000
1000
0
response
NS.
NS.
NS.
inh−
inh+cefo−
inh+cefo+
NS >0.05, * <0.05, ** < 0.01, *** <0.001
inhibition in mm
0
10
20
30
inhibition & MIC
inh−
inh+cefo−
inh+cefo+
AB equal or above MIC
0
1
B
LytB
y−axis = log10−scale
1000
300
100
response
inh−
inh+cefo−
inh+cefo+
inhibition in mm
0
10
20
30
inhibition & MIC
inh−
inh+cefo−
inh+cefo+
AB equal or above MIC
0
1

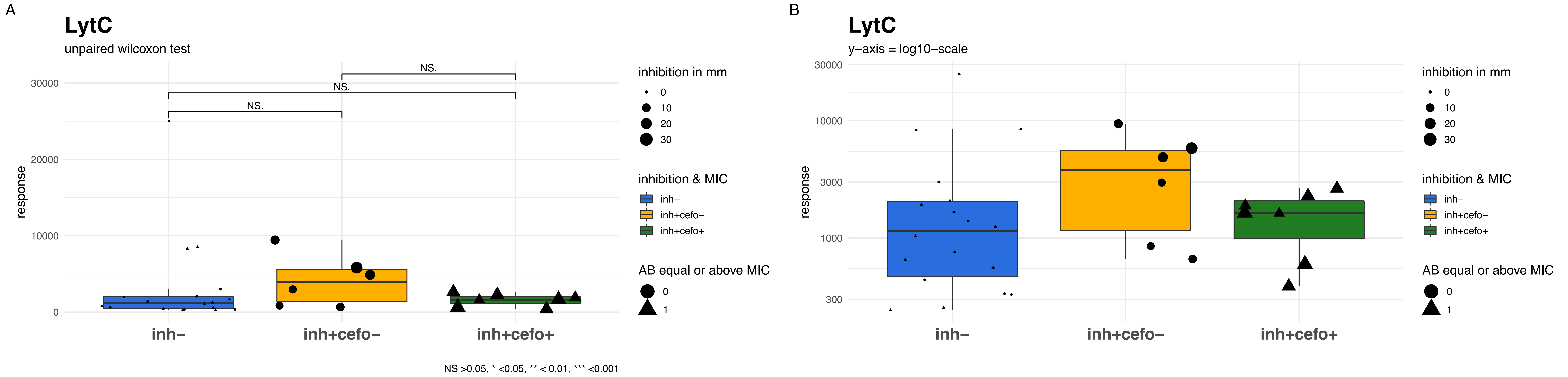

A
LytC
unpaired wilcoxon test
NS.
NS.
NS.
30000
20000
10000
0
response
inh−
inh+cefo−
inh+cefo+
NS >0.05, * <0.05, ** < 0.01, *** <0.001
inhibition in mm
0
10
20
30
inhibition & MIC
inh−
inh+cefo−
inh+cefo+
AB equal or above MIC
0
1
B
LytC
y−axis = log10−scale
30000
10000
3000
1000
300
response
inh−
inh+cefo−
inh+cefo+
inhibition in mm
0
10
20
30
inhibition & MIC
inh−
inh+cefo−
inh+cefo+
AB equal or above MIC
0
1

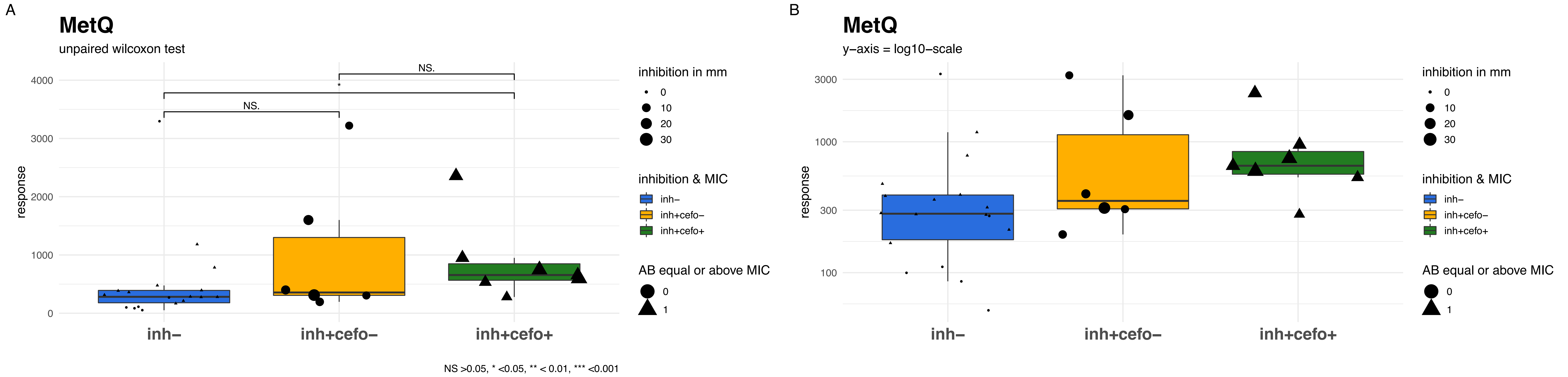
A
MetQ
unpaired wilcoxon test
NS.
*
NS.
4000
3000
2000
1000
0
response
inh−
inh+cefo−
inh+cefo+
inhibition in mm
0
10
20
30
inhibition & MIC
inh−
inh+cefo−
inh+cefo+
AB equal or above MIC
0
1
NS >0.05, * <0.05, ** < 0.01, *** <0.001
B
MetQ
y−axis = log10−scale
3000
1000
300
100
response
inh−
inh+cefo−
inh+cefo+
inhibition in mm
0
10
20
30
inhibition & MIC
inh−
inh+cefo−
inh+cefo+
AB equal or above MIC
0
1

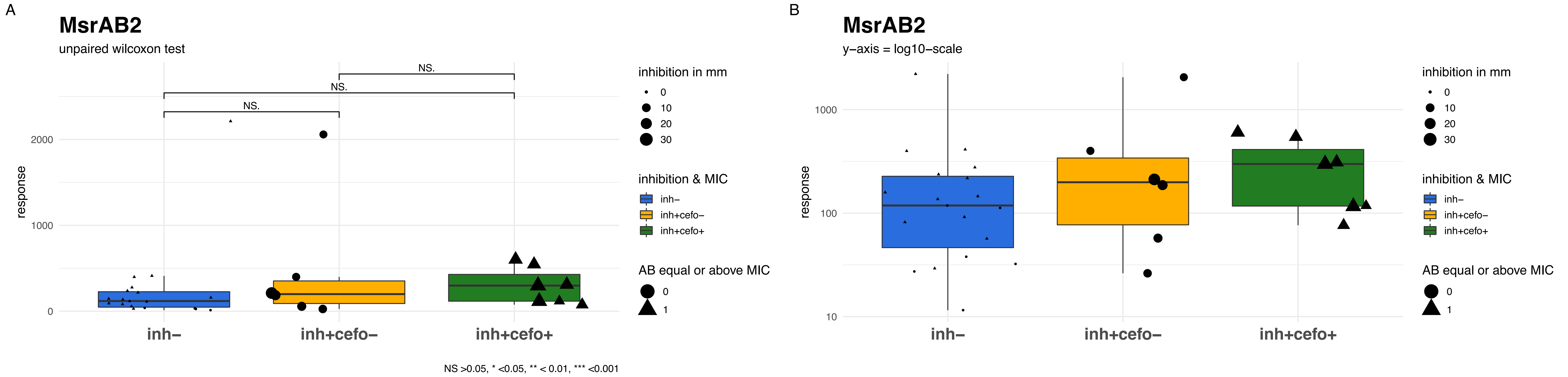

A
MsrAB2
unpaired wilcoxon test
NS.
NS.
NS.
response
2000
1000
0
inh−
inh+cefo−
inh+cefo+
NS >0.05, * <0.05, ** < 0.01, *** <0.001
inhibition in mm
0
10
20
30
inhibition & MIC
inh−
inh+cefo−
inh+cefo+
AB equal or above MIC
0
1
B
MsrAB2
y−axis = log10−scale
response
1000
100
10
inh−
inh+cefo−
inh+cefo+
inhibition in mm
0
10
20
30
inhibition & MIC
inh−
inh+cefo−
inh+cefo+
AB equal or above MIC
0
1

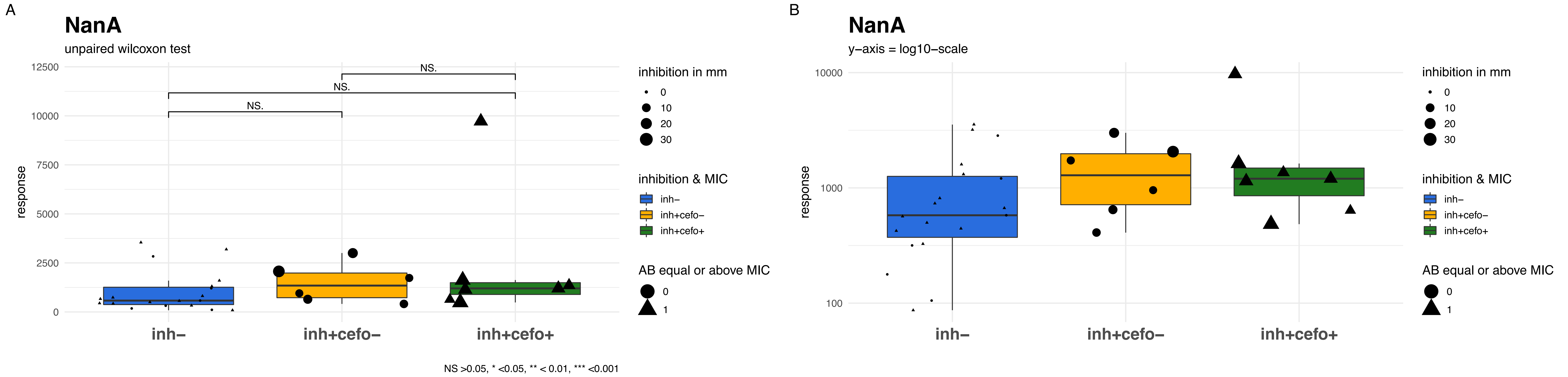
A
NanA
unpaired wilcoxon test
response
inh–
inh+cefo–
inh+cefo+
NS.
inhibition in mm
inhibition & MIC
AB equal or above MIC
NS >0.05, * <0.05, ** < 0.01, *** <0.001
B
NanA
y–axis = log10–scale

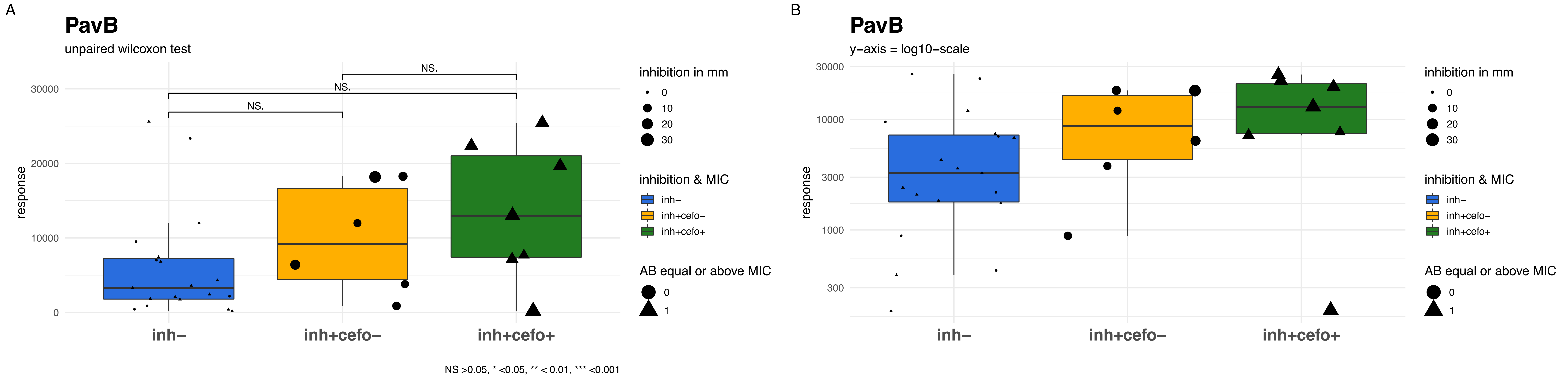

A
PavB
unpaired wilcoxon test
NS.
NS.
NS.
30000
20000
10000
0
response
inh−
inh+cefo−
inh+cefo+
inhibition in mm
0
10
20
30
inhibition & MIC
inh−
inh+cefo−
inh+cefo+
AB equal or above MIC
0
1
NS >0.05, * <0.05, ** < 0.01, *** <0.001
B
PavB
y−axis = log10−scale
30000
10000
3000
1000
300
response
inh−
inh+cefo−
inh+cefo+
inhibition in mm
0
10
20
30
inhibition & MIC
inh−
inh+cefo−
inh+cefo+
AB equal or above MIC
0
1

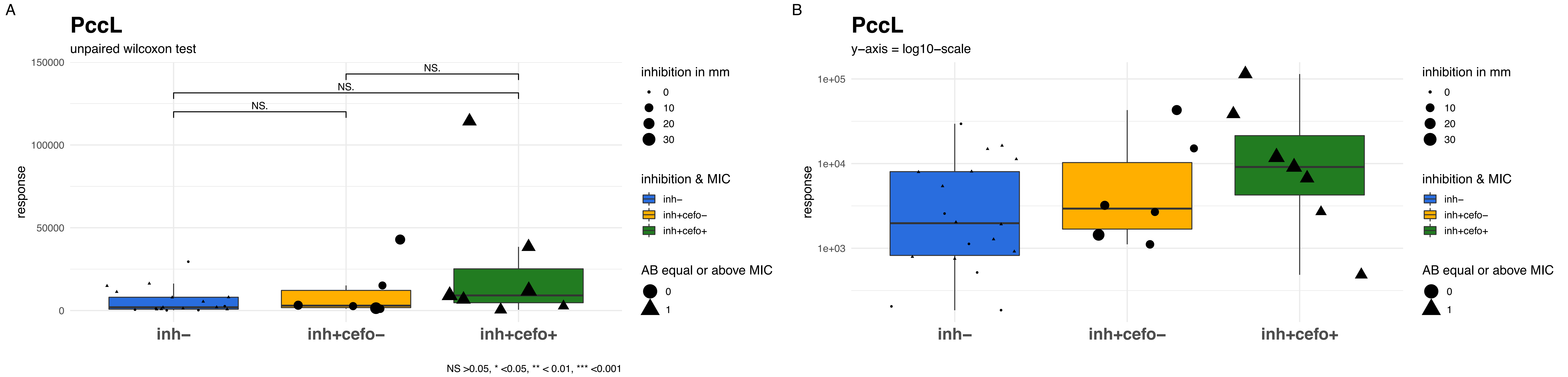
A
PccL
unpaired wilcoxon test
NS.
NS.
NS.
150000
100000
50000
0
response
inh−
inh+cefo−
inh+cefo+
NS >0.05, * <0.05, ** < 0.01, *** <0.001
inhibition in mm
0
10
20
30
inhibition & MIC
inh−
inh+cefo−
inh+cefo+
AB equal or above MIC
0
1
B
PccL
y−axis = log10−scale
1e+05
1e+04
1e+03
response
inh−
inh+cefo−
inh+cefo+
inhibition in mm
0
10
20
30
inhibition & MIC
inh−
inh+cefo−
inh+cefo+
AB equal or above MIC
0
1

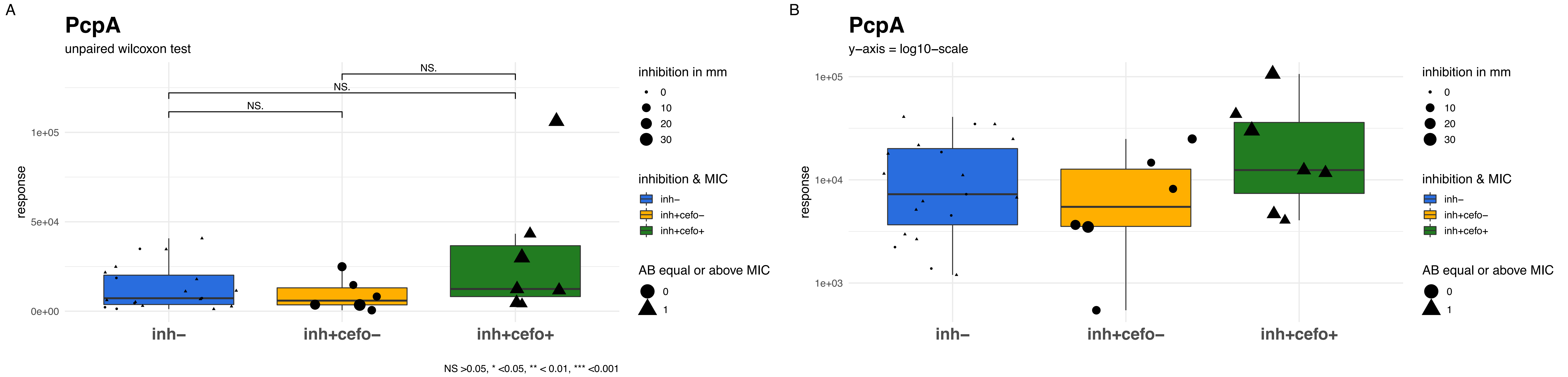
A
PcpA
unpaired wilcoxon test
NS.
NS.
NS.
response
1e+05
5e+04
0e+00
inh−
inh+cefo−
inh+cefo+
inhibition in mm
0
10
20
30
inhibition & MIC
inh−
inh+cefo−
inh+cefo+
AB equal or above MIC
0
1
NS >0.05, * <0.05, ** < 0.01, *** <0.001
B
PcpA
y−axis = log10−scale
response
1e+05
1e+04
1e+03
inh−
inh+cefo−
inh+cefo+
inhibition in mm
0
10
20
30
inhibition & MIC
inh−
inh+cefo−
inh+cefo+
AB equal or above MIC
0
1

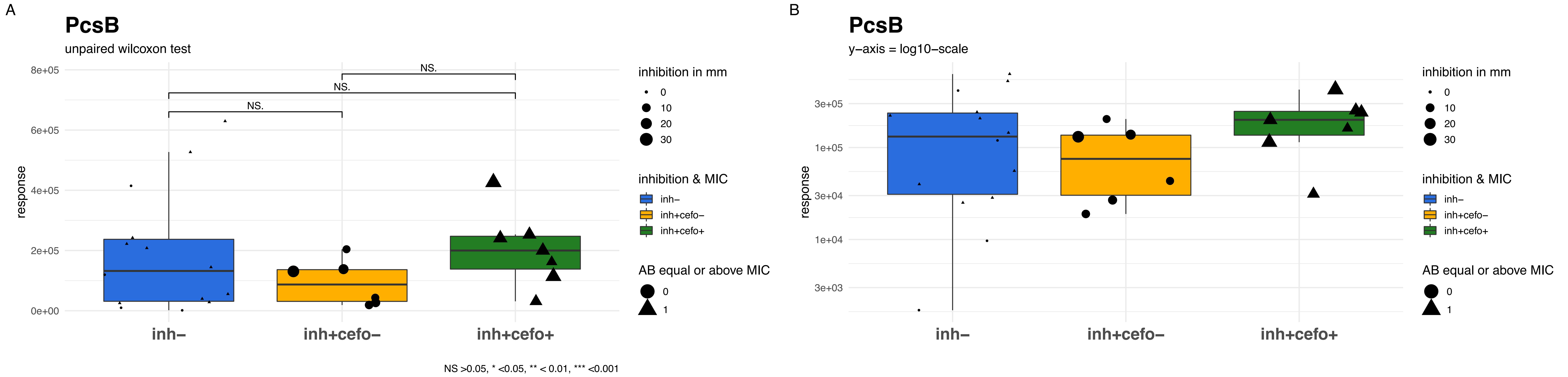
A
PcsB
unpaired wilcoxon test
8e+05
6e+05
4e+05
2e+05
0e+00
response
NS.
NS.
NS.
inh−
inh+cefo−
inh+cefo+
NS >0.05, * <0.05, ** < 0.01, *** <0.001
inhibition in mm
0
10
20
30
inhibition & MIC
inh−
inh+cefo−
inh+cefo+
AB equal or above MIC
0
1
B
PcsB
y−axis = log10−scale
3e+05
1e+05
3e+04
1e+04
3e+03
response
inh−
inh+cefo−
inh+cefo+
inhibition in mm
0
10
20
30
inhibition & MIC
inh−
inh+cefo−
inh+cefo+
AB equal or above MIC
0
1

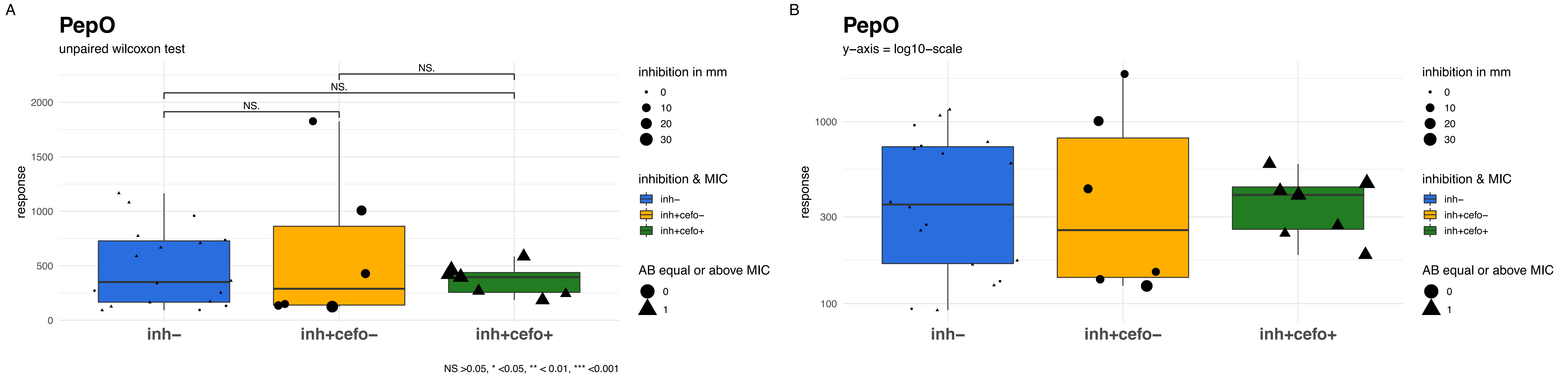
A
PepO
unpaired wilcoxon test
NS.
NS.
NS.
response
2000
1500
1000
500
0
inh−
inh+cefo−
inh+cefo+
inhibition in mm
0
10
20
30
inhibition & MIC
inh−
inh+cefo−
inh+cefo+
AB equal or above MIC
0
1
NS >0.05, * <0.05, ** < 0.01, *** <0.001
B
PepO
y−axis = log10−scale
response
1000
300
100
inh−
inh+cefo−
inh+cefo+
inhibition in mm
0
10
20
30
inhibition & MIC
inh−
inh+cefo−
inh+cefo+
AB equal or above MIC
0
1

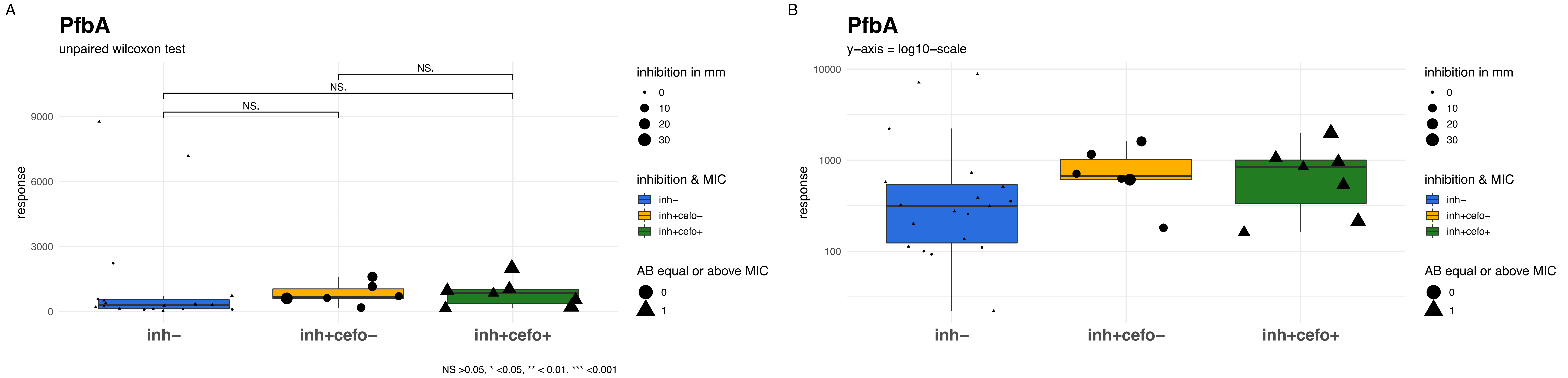

A
PfbA
unpaired wilcoxon test
NS.
NS.
NS.
response
9000
6000
3000
0
inh−
inh+cefo−
inh+cefo+
inhibition in mm
0
10
20
30
inhibition & MIC
inh−
inh+cefo−
inh+cefo+
AB equal or above MIC
0
1
NS >0.05, * <0.05, ** < 0.01, *** <0.001
B
PfbA
y−axis = log10−scale
10000
1000
100
response
inh−
inh+cefo−
inh+cefo+
inhibition in mm
0
10
20
30
inhibition & MIC
inh−
inh+cefo−
inh+cefo+
AB equal or above MIC
0
1

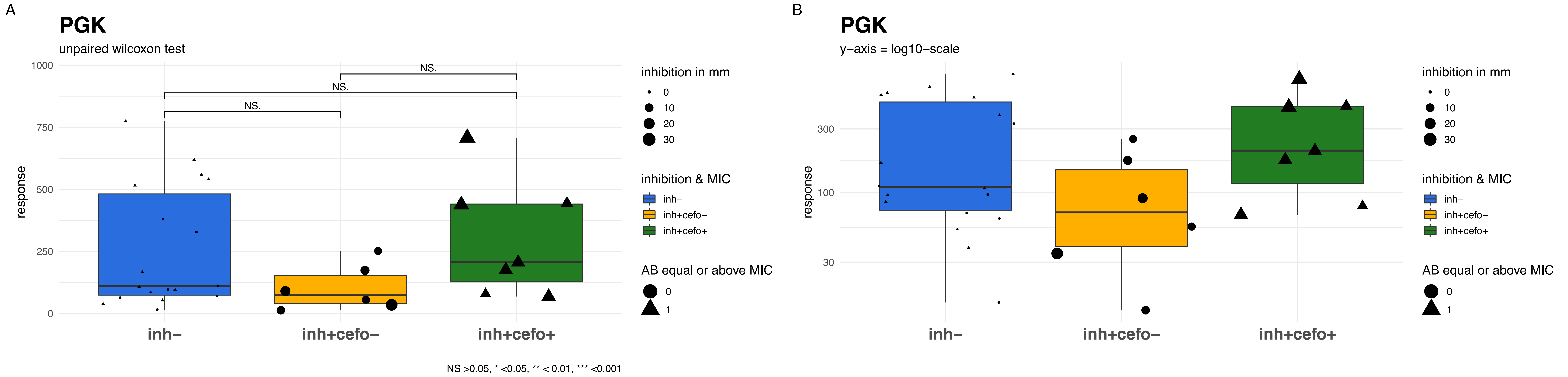
A
PGK
unpaired wilcoxon test
response
1000
750
500
250
0
NS.
NS.
NS.
inh−
inh+cefo−
inh+cefo+
inhibition in mm
0
10
20
30
inhibition & MIC
inh−
inh+cefo−
inh+cefo+
AB equal or above MIC
0
1
NS >0.05, * <0.05, ** < 0.01, *** <0.001
B
PGK
y−axis = log10−scale
response
300
100
30
inh−
inh+cefo−
inh+cefo+
inhibition in mm
0
10
20
30
inhibition & MIC
inh−
inh+cefo−
inh+cefo+
AB equal or above MIC
0
1

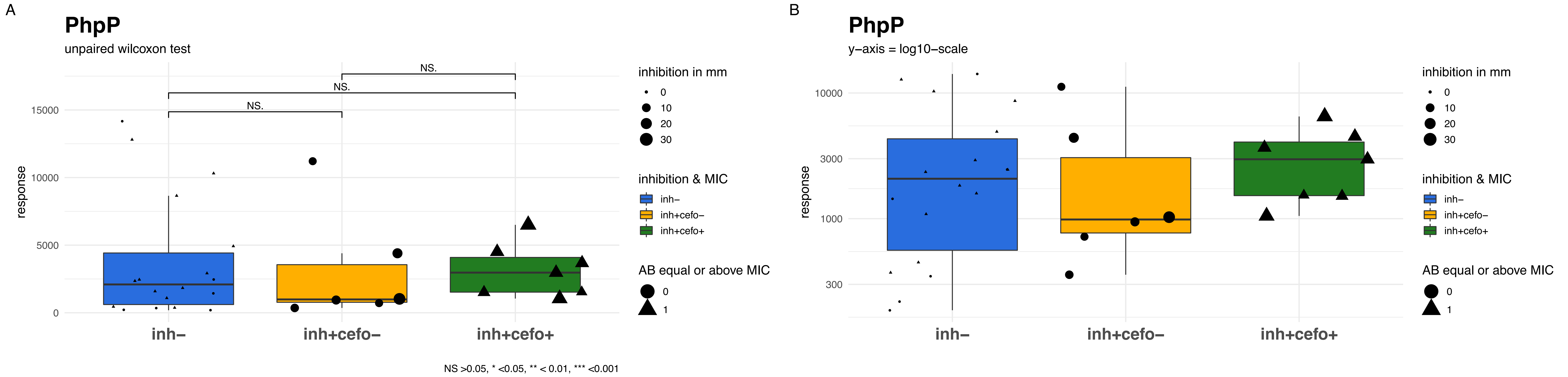
A
PhpP
unpaired wilcoxon test
NS.
NS.
NS.
15000
10000
5000
0
response
inh−
inh+cefo−
inh+cefo+
inhibition in mm
0
10
20
30
inhibition & MIC
inh−
inh+cefo−
inh+cefo+
AB equal or above MIC
0
1
NS >0.05, * <0.05, ** < 0.01, *** <0.001
B
PhpP
y−axis = log10−scale
10000
3000
1000
300
response
inh−
inh+cefo−
inh+cefo+
inhibition in mm
0
10
20
30
inhibition & MIC
inh−
inh+cefo−
inh+cefo+
AB equal or above MIC
0
1

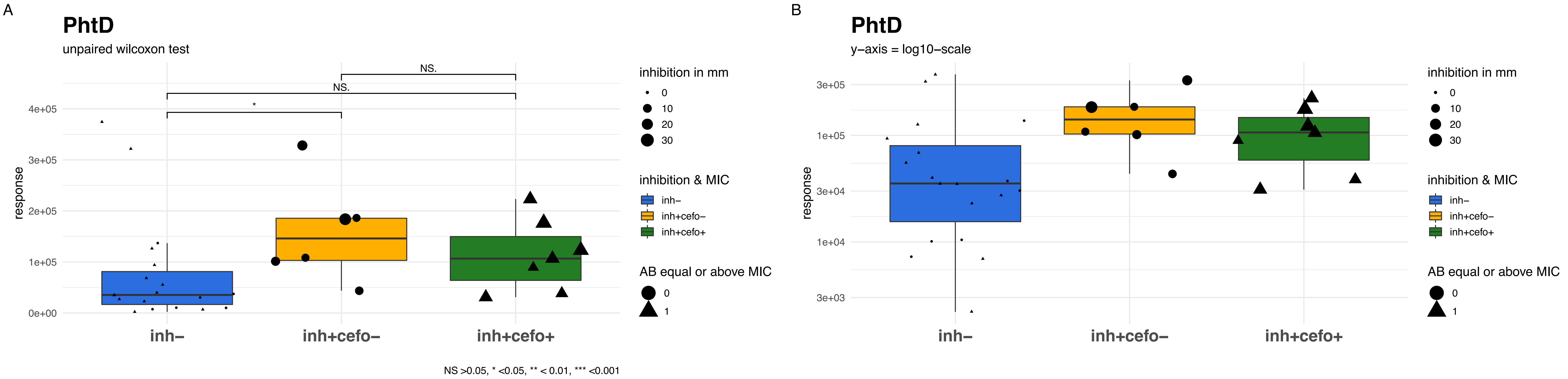

A
PhtD
unpaired wilcoxon test
NS.
NS.
*
response
4e+05
3e+05
2e+05
1e+05
0e+00
inh–
inh+cefo–
inh+cefo+
NS >0.05, * <0.05, ** < 0.01, *** <0.001
inhibition in mm
0
10
20
30
inhibition & MIC
inh–
inh+cefo–
inh+cefo+
AB equal or above MIC
0
1
B
PhtD
y–axis = log10–scale
response
3e+05
1e+05
3e+04
1e+04
3e+03
inh–
inh+cefo–
inh+cefo+
inhibition in mm
0
10
20
30
inhibition & MIC
inh–
inh+cefo–
inh+cefo+
AB equal or above MIC
0
1

A

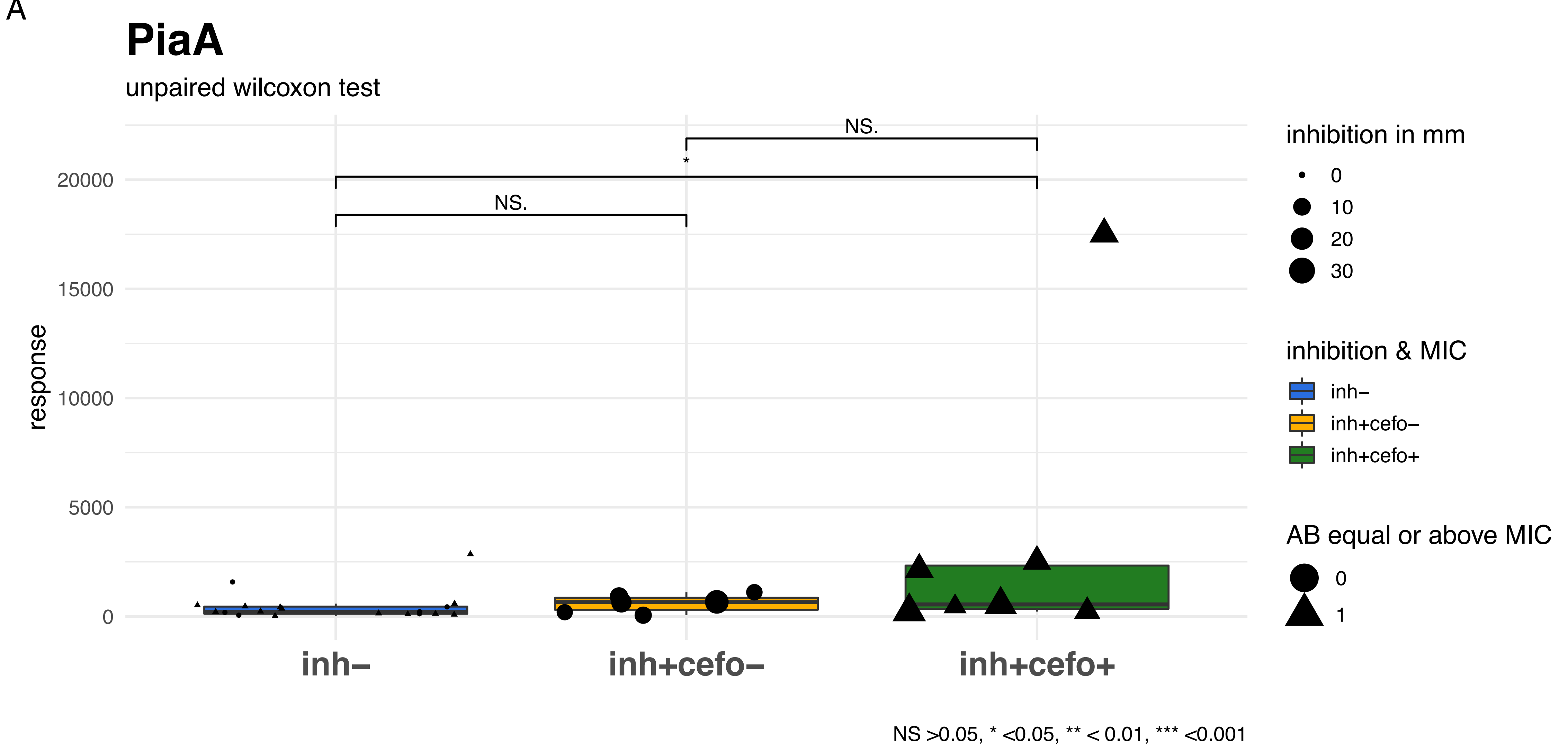
PiaA
unpaired wilcoxon test
NS.
*
NS.
20000
15000
10000
5000
0
response
inh−
inh+cefo−
inh+cefo+
inhibition in mm
0
10
20
30
inhibition & MIC
inh−
inh+cefo−
inh+cefo+
AB equal or above MIC
0
1
NS >0.05, * <0.05, ** < 0.01, *** <0.001

B

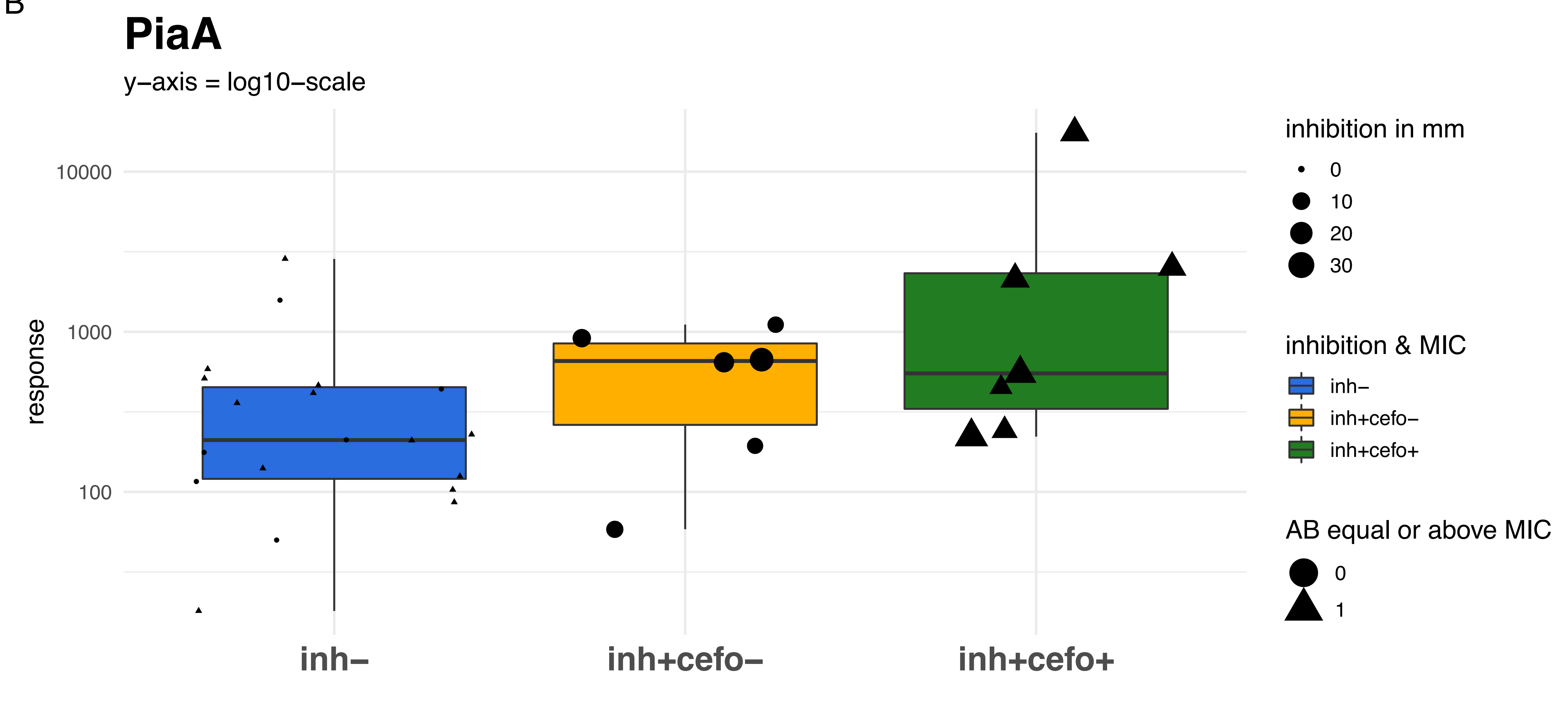
PiaA
y−axis = log10−scale
10000
1000
100
response
inh−
inh+cefo−
inh+cefo+
inhibition in mm
0
10
20
30
inhibition & MIC
inh−
inh+cefo−
inh+cefo+
AB equal or above MIC
0
1

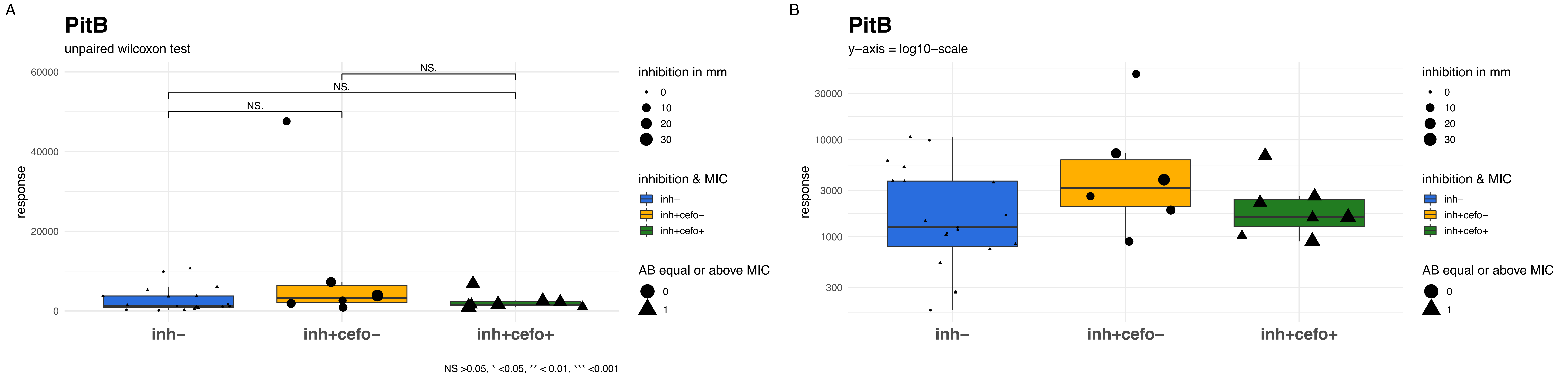

A
PitB
unpaired wilcoxon test
60000
40000
20000
0
response
NS.
NS.
NS.
inh−
inh+cefo−
inh+cefo+
NS >0.05, * <0.05, ** < 0.01, *** <0.001
inhibition in mm
0
10
20
30
inhibition & MIC
inh−
inh+cefo−
inh+cefo+
AB equal or above MIC
0
1
B
PitB
y−axis = log10−scale
30000
10000
3000
1000
300
response
inh−
inh+cefo−
inh+cefo+
inhibition in mm
0
10
20
30
inhibition & MIC
inh−
inh+cefo−
inh+cefo+
AB equal or above MIC
0
1

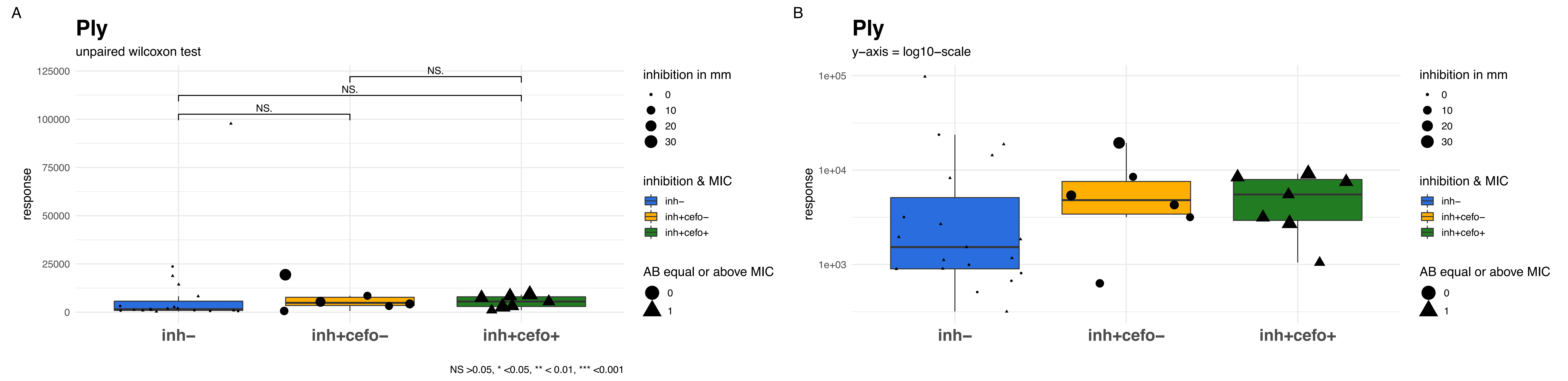
A
Ply
unpaired wilcoxon test
125000
100000
75000
50000
25000
0
response
NS.
NS.
NS.
inh−
inh+cefo−
inh+cefo+
inhibition in mm
0
10
20
30
inhibition & MIC
inh−
inh+cefo−
inh+cefo+
AB equal or above MIC
0
1
NS >0.05, * <0.05, ** < 0.01, *** <0.001
B
Ply
y−axis = log10−scale
1e+05
1e+04
1e+03
response
inh−
inh+cefo−
inh+cefo+
inhibition in mm
0
10
20
30
inhibition & MIC
inh−
inh+cefo−
inh+cefo+
AB equal or above MIC
0
1

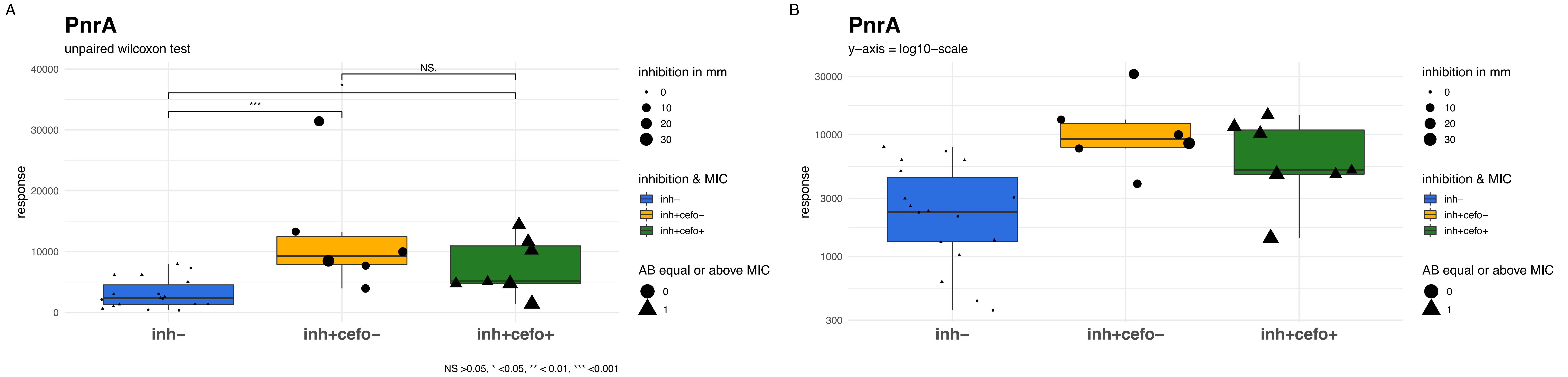

A
PnrA
unpaired wilcoxon test
40000
30000
20000
10000
0
response
NS.
*
***
inh−
inh+cefo−
inh+cefo+
inhibition in mm
0
10
20
30
inhibition & MIC
inh−
inh+cefo−
inh+cefo+
AB equal or above MIC
0
1
NS >0.05, * <0.05, ** < 0.01, *** <0.001
B
PnrA
y−axis = log10−scale
30000
10000
3000
1000
300
response
inh−
inh+cefo−
inh+cefo+
inhibition in mm
0
10
20
30
inhibition & MIC
inh−
inh+cefo−
inh+cefo+
AB equal or above MIC
0
1

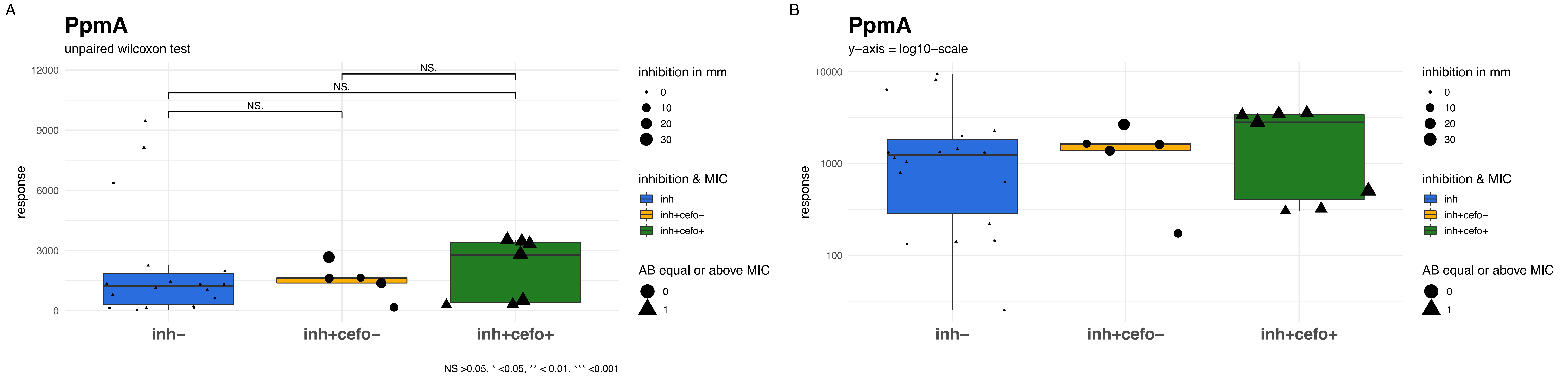

A
PpmA
unpaired wilcoxon test
12000
9000
6000
3000
0
response
NS.
NS.
NS.
inh−
inh+cefo−
inh+cefo+
NS >0.05, * <0.05, ** < 0.01, *** <0.001
inhibition in mm
0
10
20
30
inhibition & MIC
inh−
inh+cefo−
inh+cefo+
AB equal or above MIC
0
1
B
PpmA
y−axis = log10−scale
10000
1000
100
response
inh−
inh+cefo−
inh+cefo+
inhibition in mm
0
10
20
30
inhibition & MIC
inh−
inh+cefo−
inh+cefo+
AB equal or above MIC
0
1

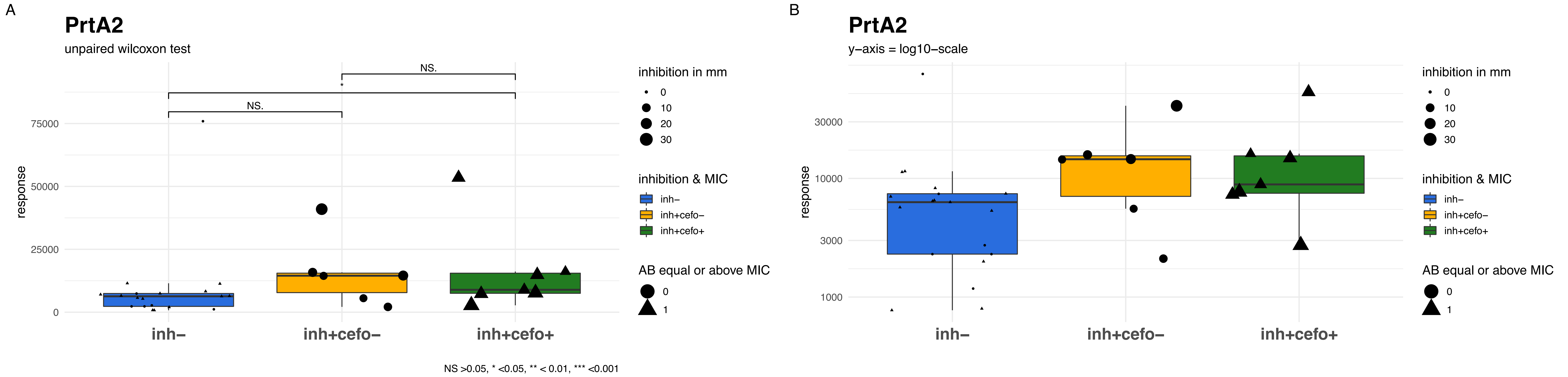

A
PrtA2
unpaired wilcoxon test
NS.
*
NS.
response
75000
50000
25000
0
inh−
inh+cefo−
inh+cefo+
inhibition in mm
0
10
20
30
inhibition & MIC
inh−
inh+cefo−
inh+cefo+
AB equal or above MIC
0
1
NS >0.05, * <0.05, ** < 0.01, *** <0.001
B
PrtA2
y−axis = log10−scale
response
30000
10000
3000
1000
inh−
inh+cefo−
inh+cefo+
inhibition in mm
0
10
20
30
inhibition & MIC
inh−
inh+cefo−
inh+cefo+
AB equal or above MIC
0
1

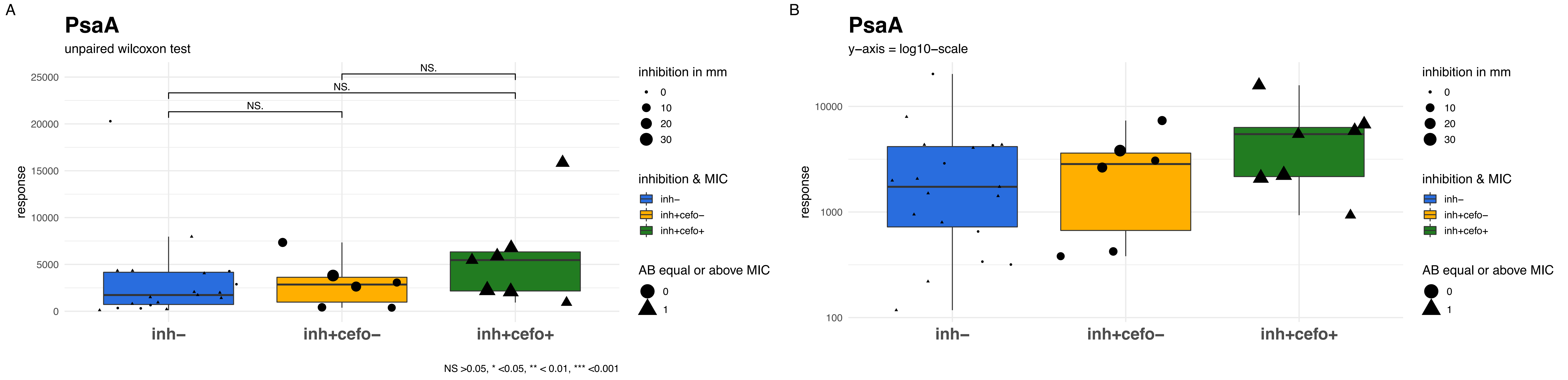

A
PsaA
unpaired wilcoxon test
NS.
NS.
NS.
response
25000
20000
15000
10000
5000
0
inh−
inh+cefo−
inh+cefo+
inhibition in mm
0
10
20
30
inhibition & MIC
inh−
inh+cefo−
inh+cefo+
AB equal or above MIC
0
1
NS >0.05, * <0.05, ** < 0.01, *** <0.001
B
PsaA
y−axis = log10−scale
response
10000
1000
100
inh−
inh+cefo−
inh+cefo+
inhibition in mm
0
10
20
30
inhibition & MIC
inh−
inh+cefo−
inh+cefo+
AB equal or above MIC
0
1

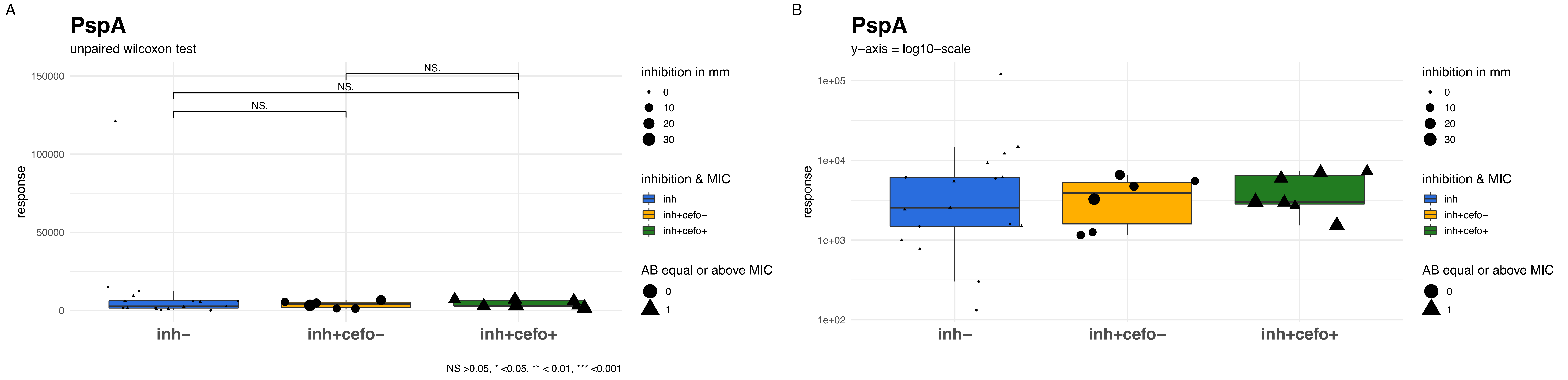
A
PspA
unpaired wilcoxon test
150000
100000
50000
0
response
NS.
NS.
NS.
inh−
inh+cefo−
inh+cefo+
inhibition in mm
0
10
20
30
inhibition & MIC
inh−
inh+cefo−
inh+cefo+
AB equal or above MIC
0
1
NS >0.05, * <0.05, ** < 0.01, *** <0.001
B
PspA
y−axis = log10−scale
1e+05
1e+04
1e+03
1e+02
response
inh−
inh+cefo−
inh+cefo+
inhibition in mm
0
10
20
30
inhibition & MIC
inh−
inh+cefo−
inh+cefo+
AB equal or above MIC
0
1

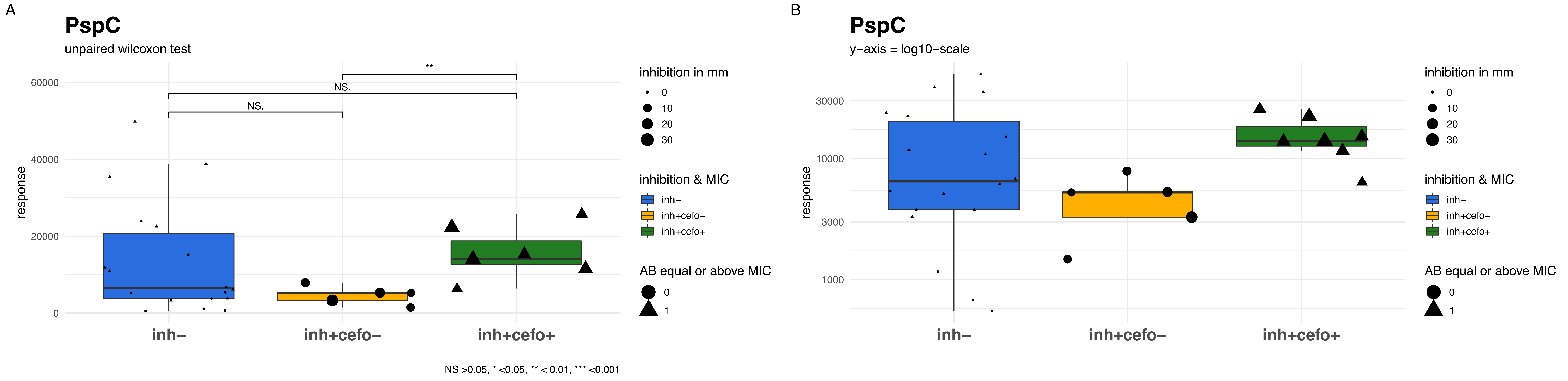
A
PspC
unpaired wilcoxon test
response
60000
40000
20000
0
**
NS.
NS.
inh−
inh+cefo−
inh+cefo+
inhibition in mm
0
10
20
30
inhibition & MIC
inh−
inh+cefo−
inh+cefo+
AB equal or above MIC
0
1
NS >0.05, * <0.05, ** < 0.01, *** <0.001
B
PspC
y−axis = log10−scale
response
30000
10000
3000
1000
inh−
inh+cefo−
inh+cefo+
inhibition in mm
0
10
20
30
inhibition & MIC
inh−
inh+cefo−
inh+cefo+
AB equal or above MIC
0
1

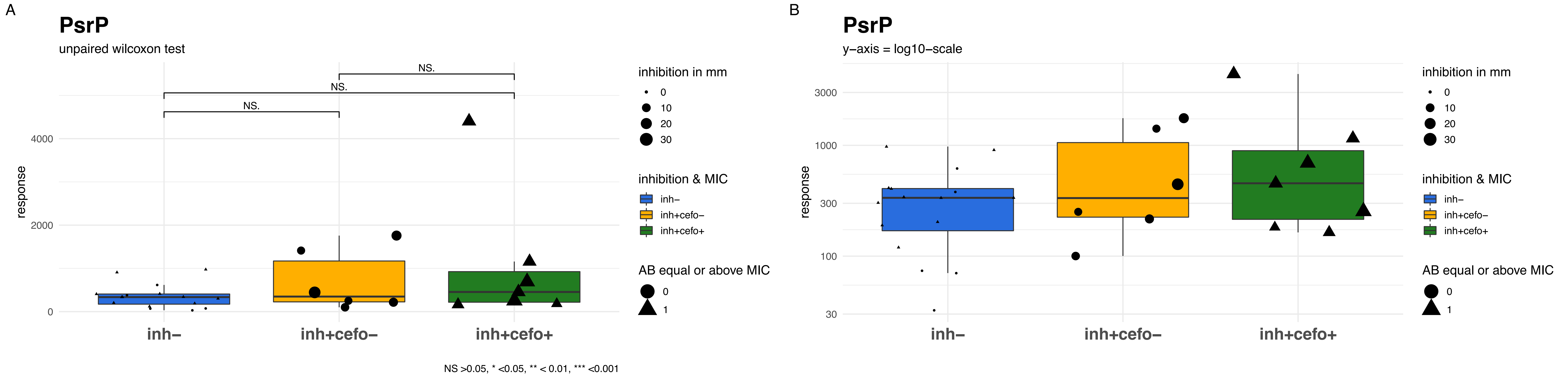
A
PsrP
unpaired wilcoxon test
NS.
NS.
NS.
response
4000
2000
0
inh−
inh+cefo−
inh+cefo+
NS >0.05, * <0.05, ** < 0.01, *** <0.001
inhibition in mm
0
10
20
30
inhibition & MIC
inh−
inh+cefo−
inh+cefo+
AB equal or above MIC
0
1
B
PsrP
y−axis = log10−scale
response
3000
1000
300
100
30
inh−
inh+cefo−
inh+cefo+
inhibition in mm
0
10
20
30
inhibition & MIC
inh−
inh+cefo−
inh+cefo+
AB equal or above MIC
0
1

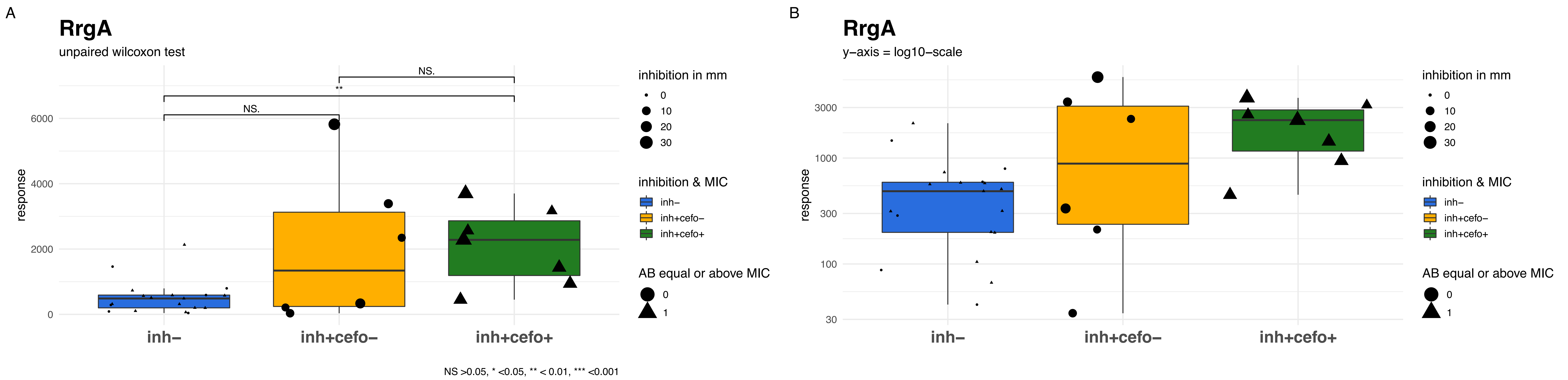
A
RrgA
unpaired wilcoxon test
NS.
**
NS.
response
6000
4000
2000
0
inh−
inh+cefo−
inh+cefo+
inhibition in mm
0
10
20
30
inhibition & MIC
inh−
inh+cefo−
inh+cefo+
AB equal or above MIC
0
1
NS >0.05, * <0.05, ** < 0.01, *** <0.001
B
RrgA
y−axis = log10−scale
response
3000
1000
300
100
30
inh−
inh+cefo−
inh+cefo+
inhibition in mm
0
10
20
30
inhibition & MIC
inh−
inh+cefo−
inh+cefo+
AB equal or above MIC
0
1

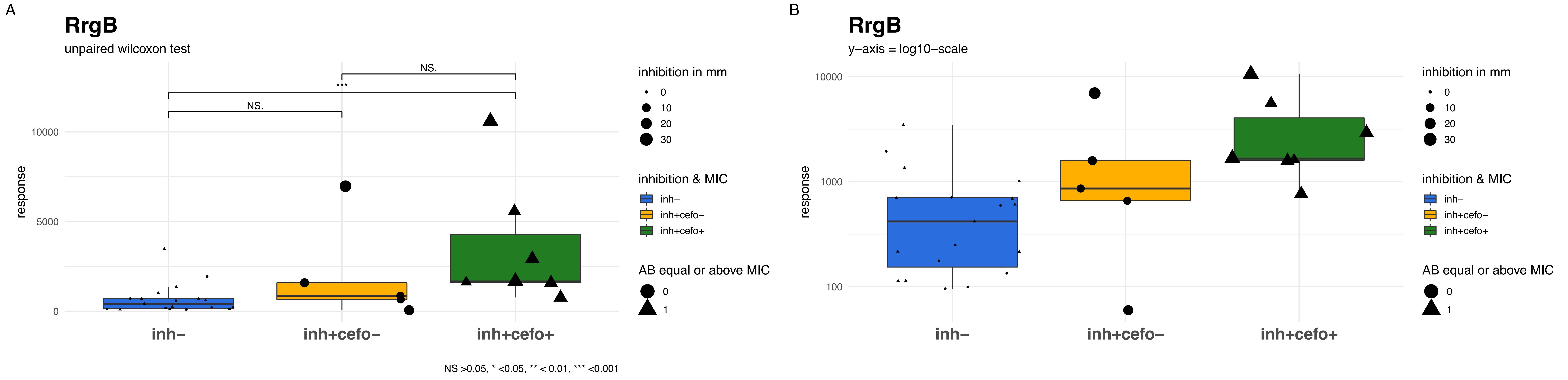

A
RrgB
unpaired wilcoxon test
NS.
***
NS.
10000
5000
0
response
inh−
inh+cefo−
inh+cefo+
inhibition in mm
0
10
20
30
inhibition & MIC
inh−
inh+cefo−
inh+cefo+
AB equal or above MIC
0
1
NS >0.05, * <0.05, ** < 0.01, *** <0.001
B
RrgB
y−axis = log10−scale
10000
1000
100
response
inh−
inh+cefo−
inh+cefo+
inhibition in mm
0
10
20
30
inhibition & MIC
inh−
inh+cefo−
inh+cefo+
AB equal or above MIC
0
1

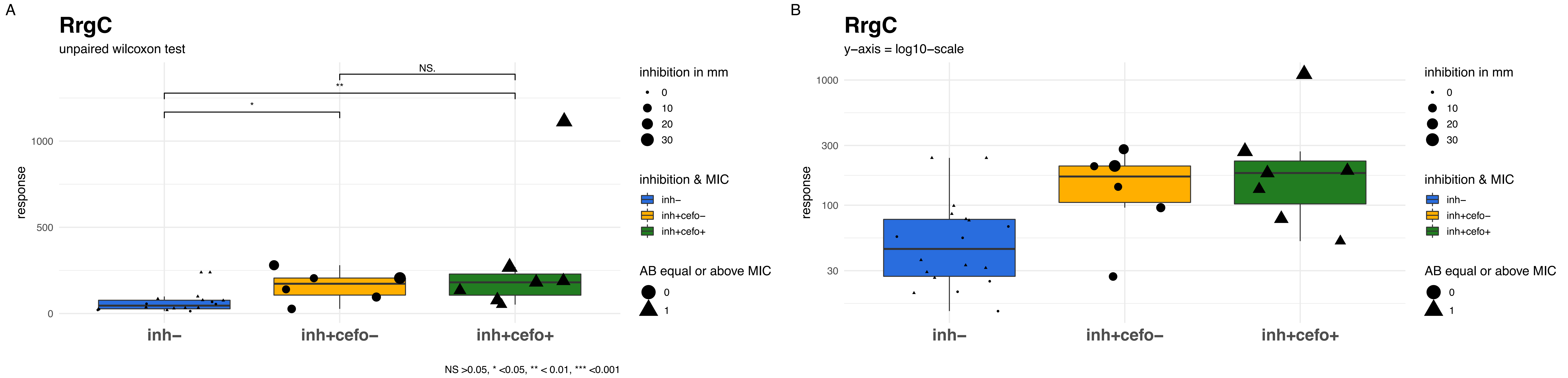
A
RrgC
unpaired wilcoxon test
NS.
**
*
1000
500
0
response
inh−
inh+cefo−
inh+cefo+
inhibition in mm
0
10
20
30
inhibition & MIC
inh−
inh+cefo−
inh+cefo+
AB equal or above MIC
0
1
NS >0.05, * <0.05, ** < 0.01, *** <0.001
B
RrgC
y−axis = log10−scale
1000
300
100
30
response
inh−
inh+cefo−
inh+cefo+
inhibition in mm
0
10
20
30
inhibition & MIC
inh−
inh+cefo−
inh+cefo+
AB equal or above MIC
0
1

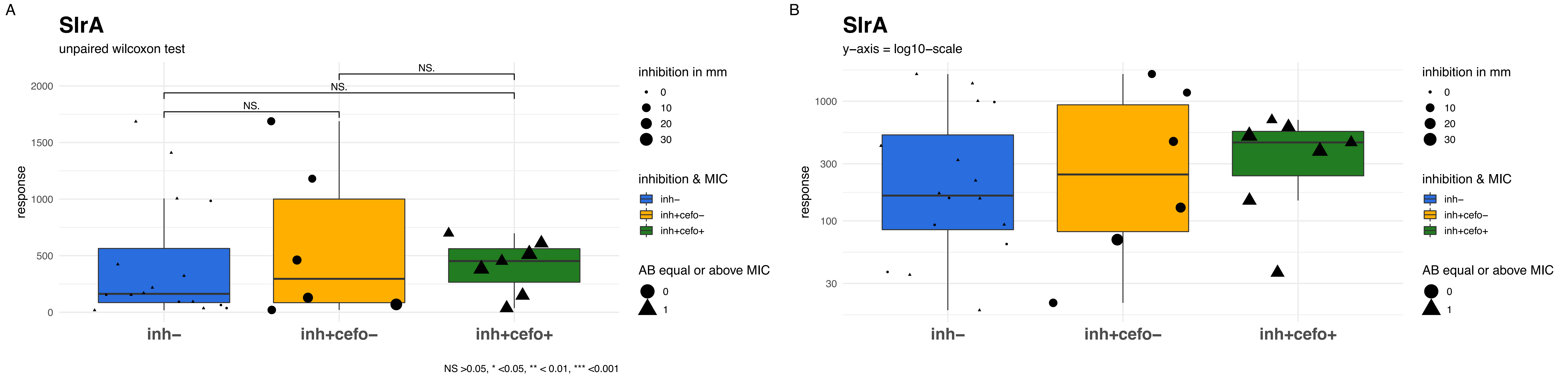
A
SlrA
unpaired wilcoxon test
NS.
NS.
NS.
2000
1500
1000
500
0
response
inh−
inh+cefo−
inh+cefo+
inhibition in mm
0
10
20
30
inhibition & MIC
inh−
inh+cefo−
inh+cefo+
AB equal or above MIC
0
1
NS >0.05, * <0.05, ** < 0.01, *** <0.001
B
SlrA
y−axis = log10−scale
1000
300
100
30
response
inh−
inh+cefo−
inh+cefo+
inhibition in mm
0
10
20
30
inhibition & MIC
inh−
inh+cefo−
inh+cefo+
AB equal or above MIC
0
1

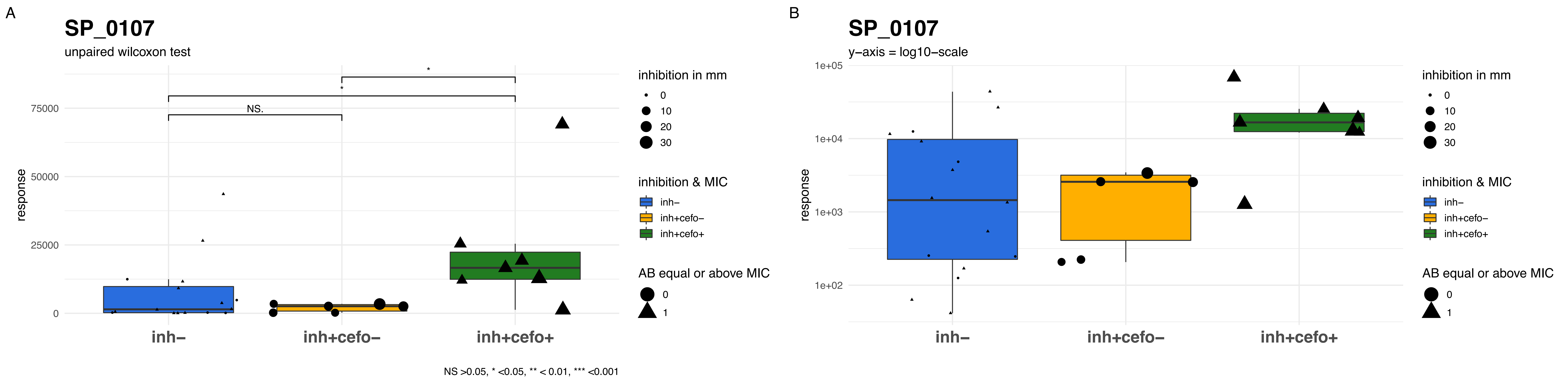
A
SP_0107
unpaired wilcoxon test
response
75000
50000
25000
0
NS.
*
*
inh−
inh+cefo−
inh+cefo+
inhibition in mm
0
10
20
30
inhibition & MIC
inh−
inh+cefo−
inh+cefo+
AB equal or above MIC
0
1
NS >0.05, * <0.05, ** < 0.01, *** <0.001
B
SP_0107
y−axis = log10−scale
response
1e+05
1e+04
1e+03
1e+02
inh−
inh+cefo−
inh+cefo+
inhibition in mm
0
10
20
30
inhibition & MIC
inh−
inh+cefo−
inh+cefo+
AB equal or above MIC
0
1

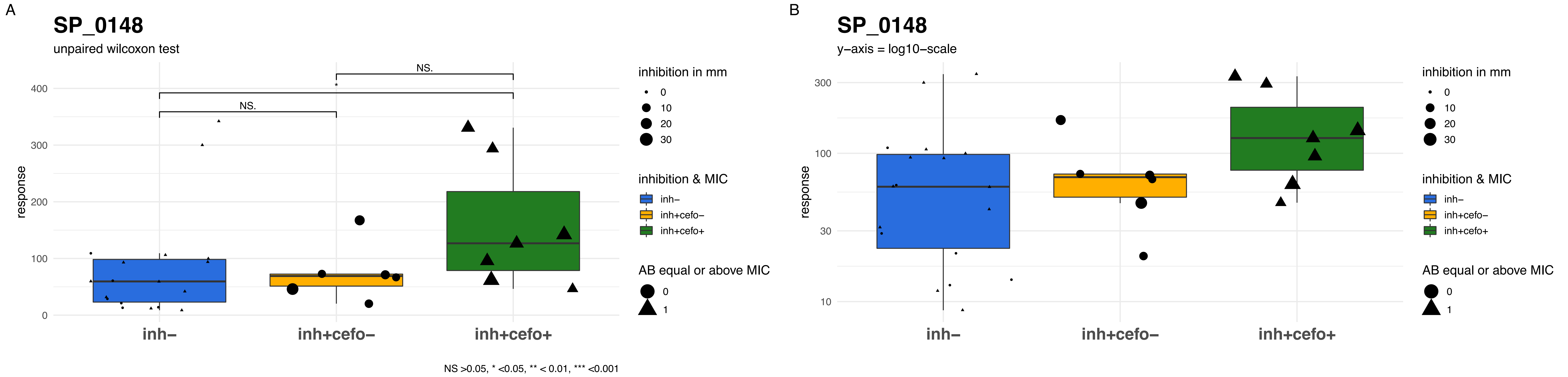

A
SP_0148
unpaired wilcoxon test
NS.
*
NS.
response
400
300
200
100
0
inh−
inh+cefo−
inh+cefo+
inhibition in mm
0
10
20
30
inhibition & MIC
inh−
inh+cefo−
inh+cefo+
AB equal or above MIC
0
1
NS >0.05, * <0.05, ** < 0.01, *** <0.001
B
SP_0148
y−axis = log10−scale
response
300
100
30
10
inh−
inh+cefo−
inh+cefo+
inhibition in mm
0
10
20
30
inhibition & MIC
inh−
inh+cefo−
inh+cefo+
AB equal or above MIC
0
1

A

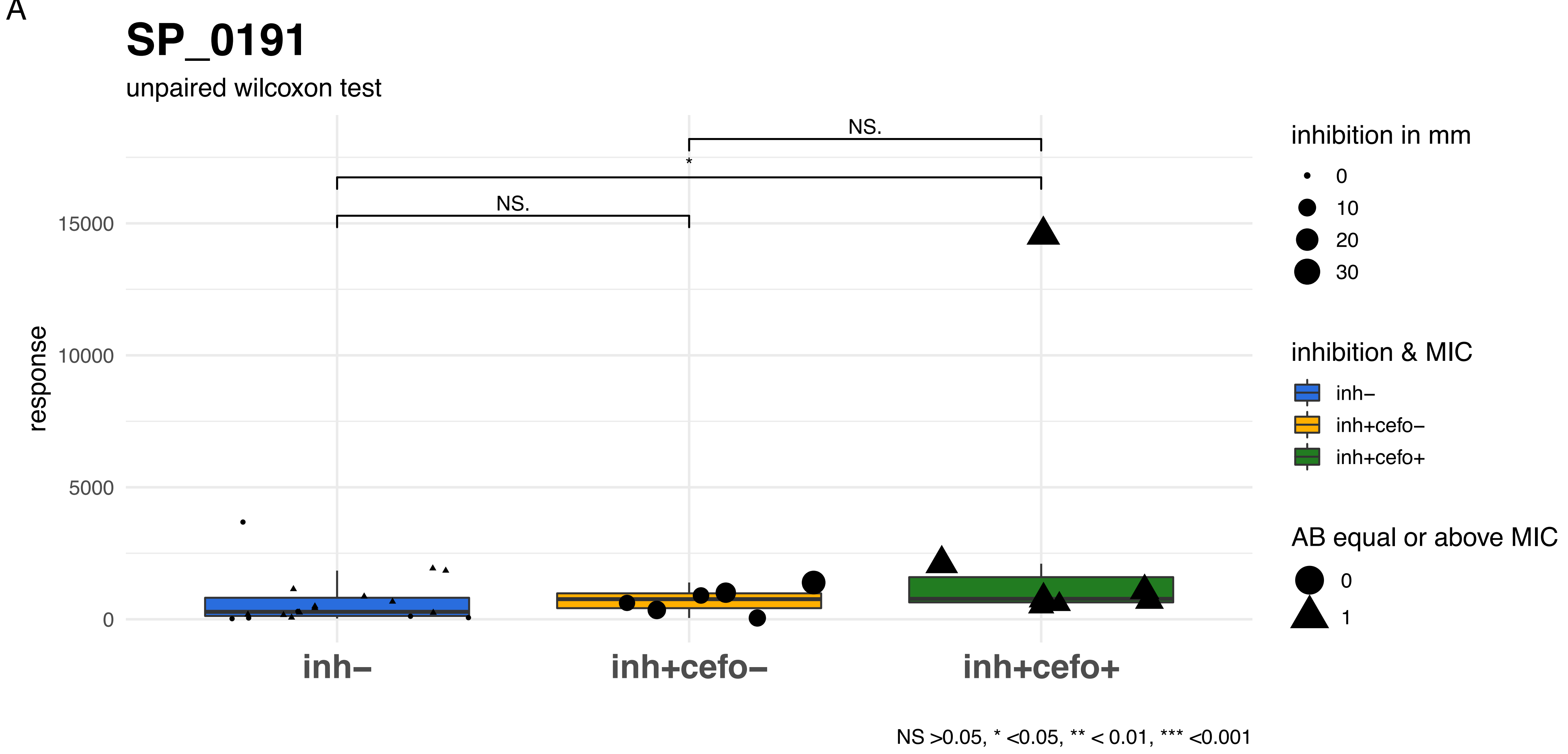

SP_0191
unpaired wilcoxon test
NS.
*
NS.
response
15000
10000
5000
0
inh−
inh+cefo−
inh+cefo+
inhibition in mm
0
10
20
30
inhibition & MIC
inh−
inh+cefo−
inh+cefo+
AB equal or above MIC
0
1
NS >0.05, * <0.05, ** < 0.01, *** <0.001

B

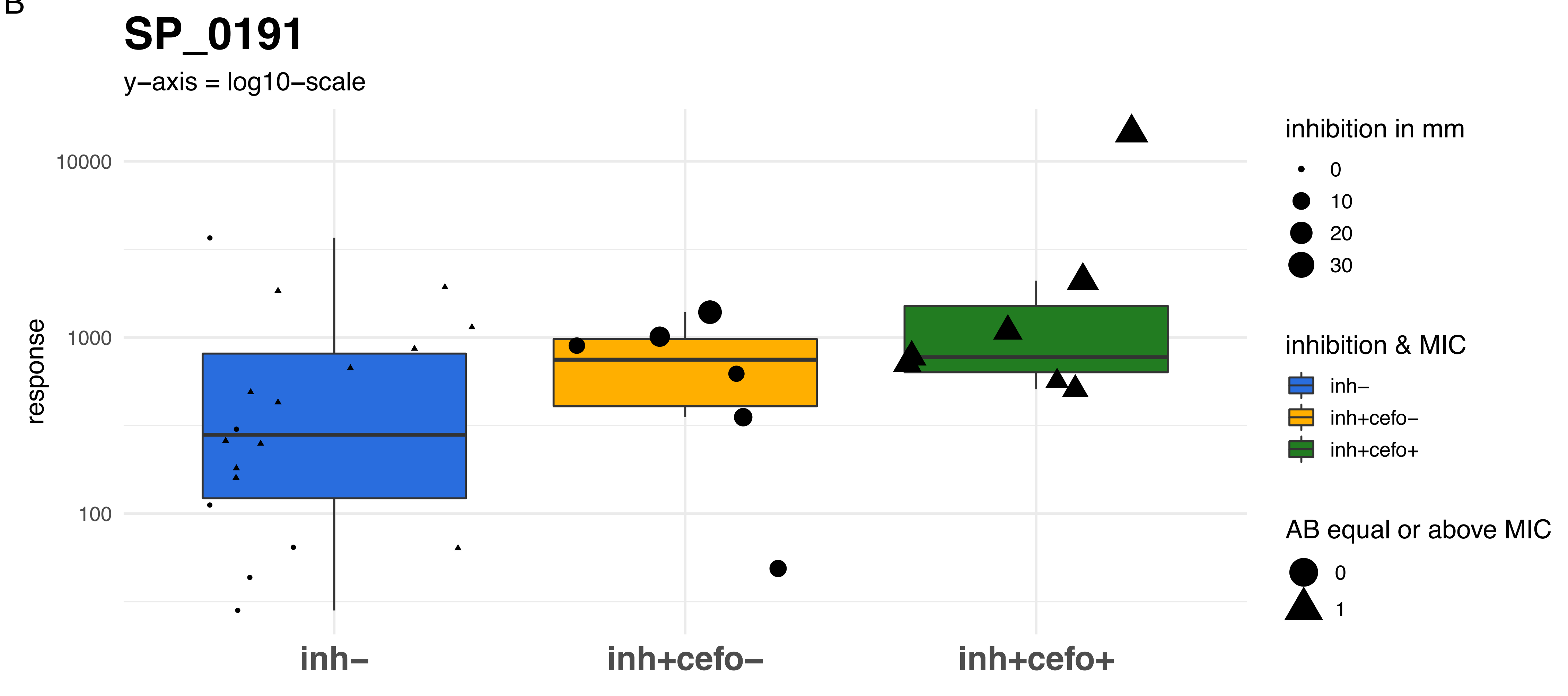

SP_0191
y−axis = log10−scale
response
10000
1000
100
inh−
inh+cefo−
inh+cefo+
inhibition in mm
0
10
20
30
inhibition & MIC
inh−
inh+cefo−
inh+cefo+
AB equal or above MIC
0
1

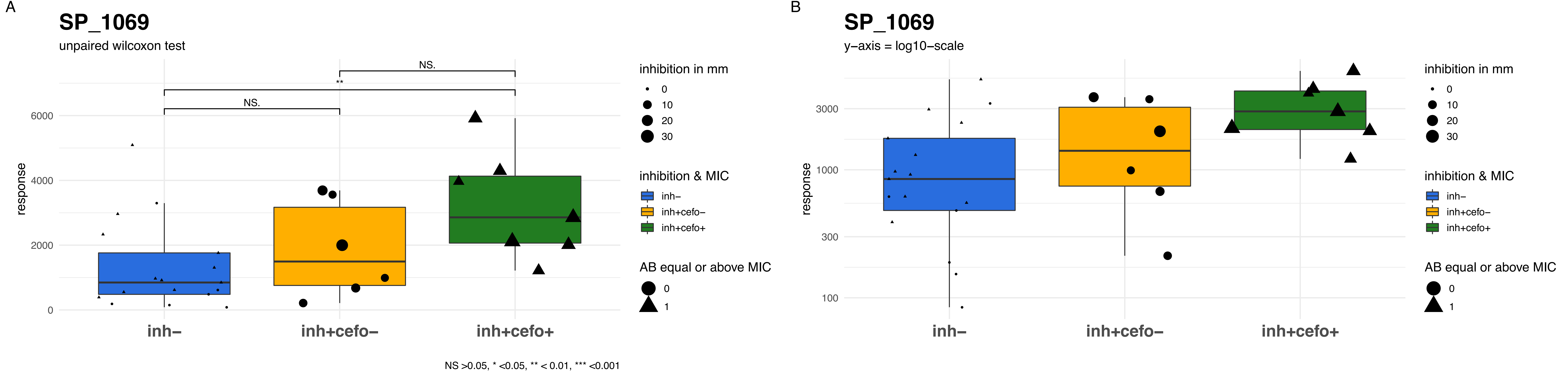

A
SP_1069
unpaired wilcoxon test
NS.
**
NS.
6000
4000
2000
0
response
inh−
inh+cefo−
inh+cefo+
inhibition in mm
0
10
20
30
inhibition & MIC
inh−
inh+cefo−
inh+cefo+
AB equal or above MIC
0
1
NS >0.05, * <0.05, ** < 0.01, *** <0.001
B
SP_1069
y−axis = log10−scale
3000
1000
300
100
response
inh−
inh+cefo−
inh+cefo+
inhibition in mm
0
10
20
30
inhibition & MIC
inh−
inh+cefo−
inh+cefo+
AB equal or above MIC
0
1

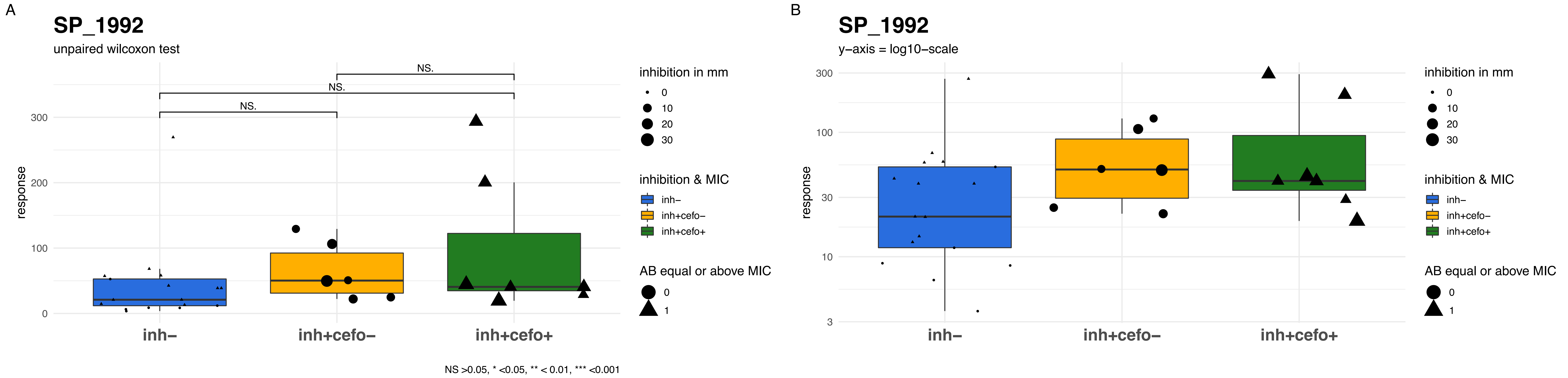
A
SP_1992
unpaired wilcoxon test
NS.
NS.
NS.
response
300
200
100
0
inh−
inh+cefo−
inh+cefo+
NS >0.05, * <0.05, ** < 0.01, *** <0.001
inhibition in mm
0
10
20
30
inhibition & MIC
inh−
inh+cefo−
inh+cefo+
AB equal or above MIC
0
1
B
SP_1992
y−axis = log10−scale
response
300
100
30
10
3
inh−
inh+cefo−
inh+cefo+
inhibition in mm
0
10
20
30
inhibition & MIC
inh−
inh+cefo−
inh+cefo+
AB equal or above MIC
0
1

A

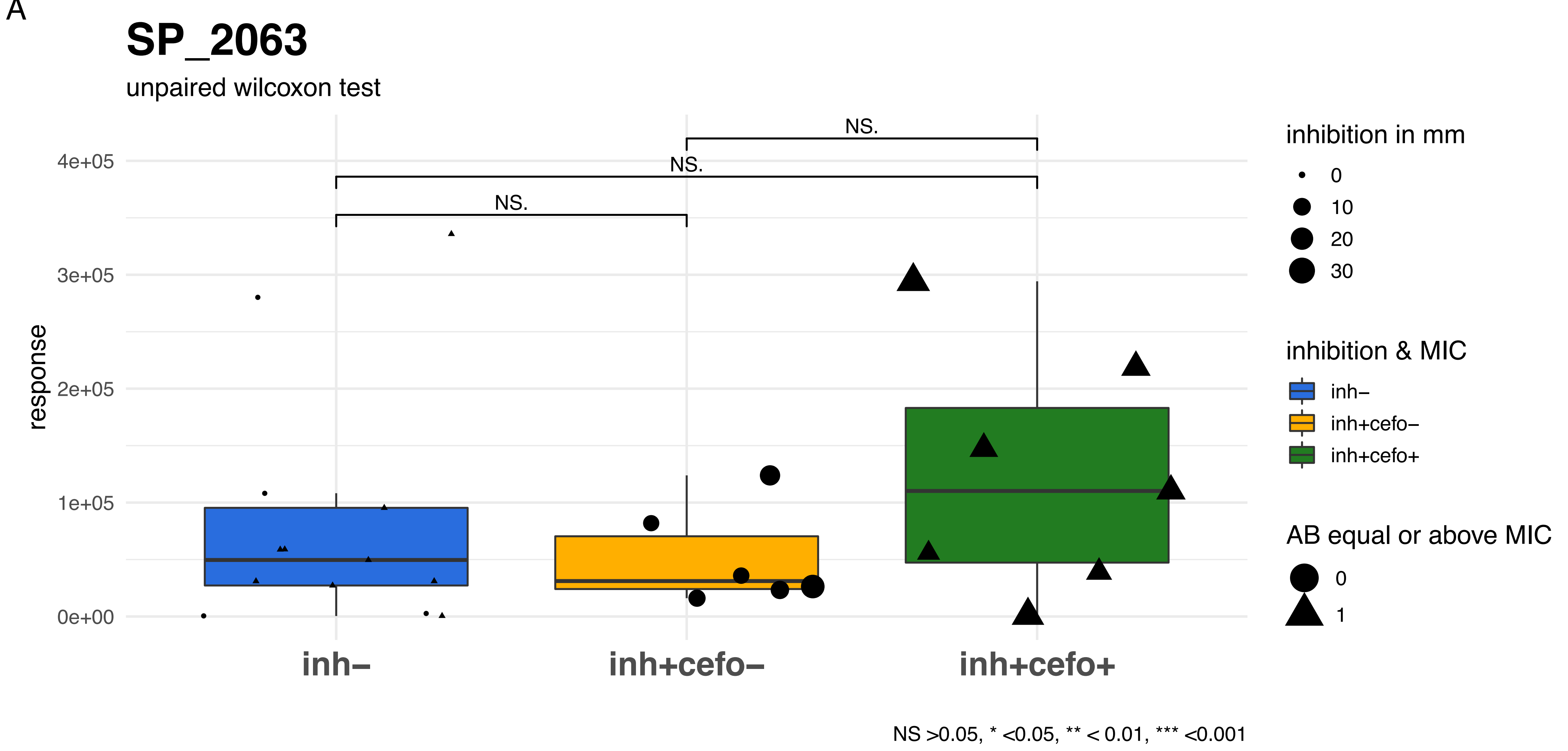
SP_2063
unpaired wilcoxon test
NS.
NS.
NS.
4e+05
3e+05
2e+05
1e+05
0e+00
response
inh−
inh+cefo−
inh+cefo+
inhibition in mm
0
10
20
30
inhibition & MIC
inh−
inh+cefo−
inh+cefo+
AB equal or above MIC
0
1
NS >0.05, * <0.05, ** < 0.01, *** <0.001

B

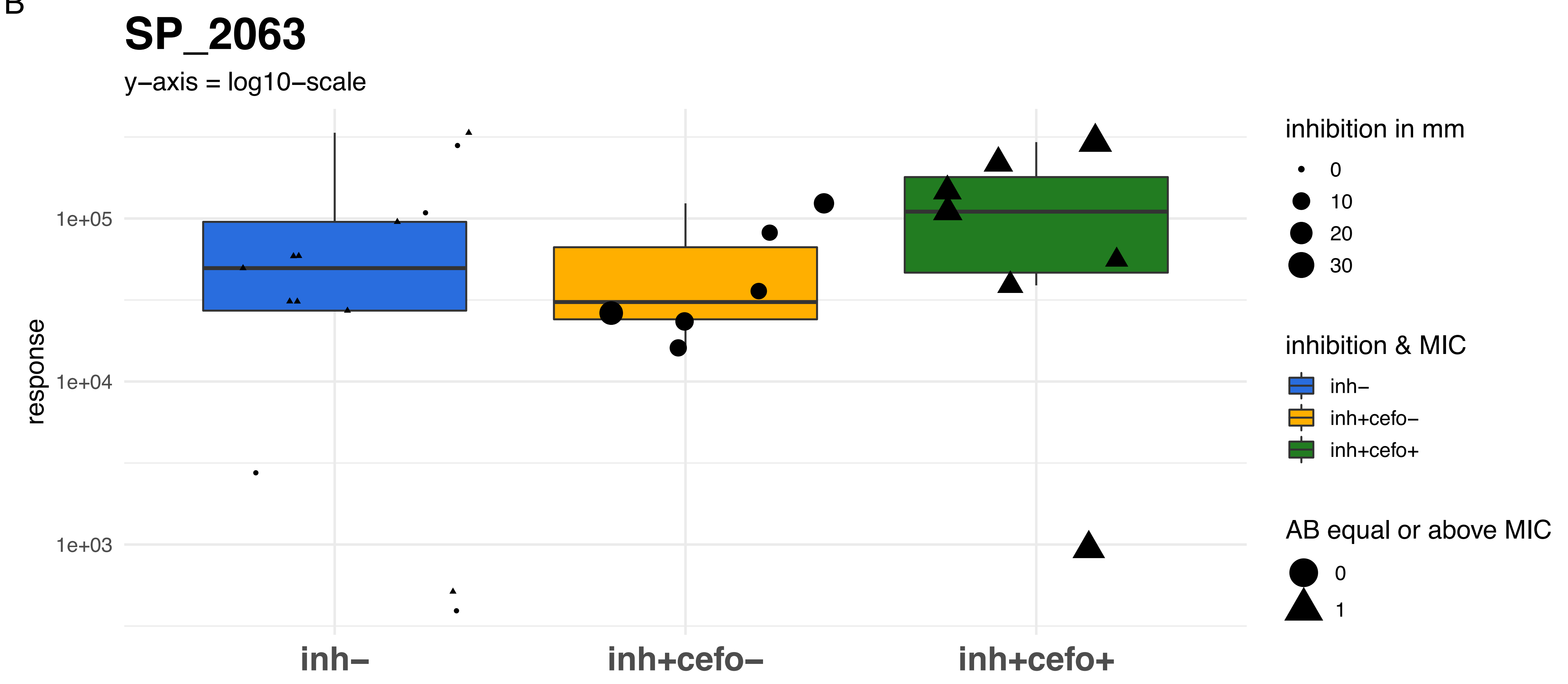
SP_2063
y−axis = log10−scale
1e+05
1e+04
1e+03
response
inh−
inh+cefo−
inh+cefo+
inhibition in mm
0
10
20
30
inhibition & MIC
inh−
inh+cefo−
inh+cefo+
AB equal or above MIC
0
1

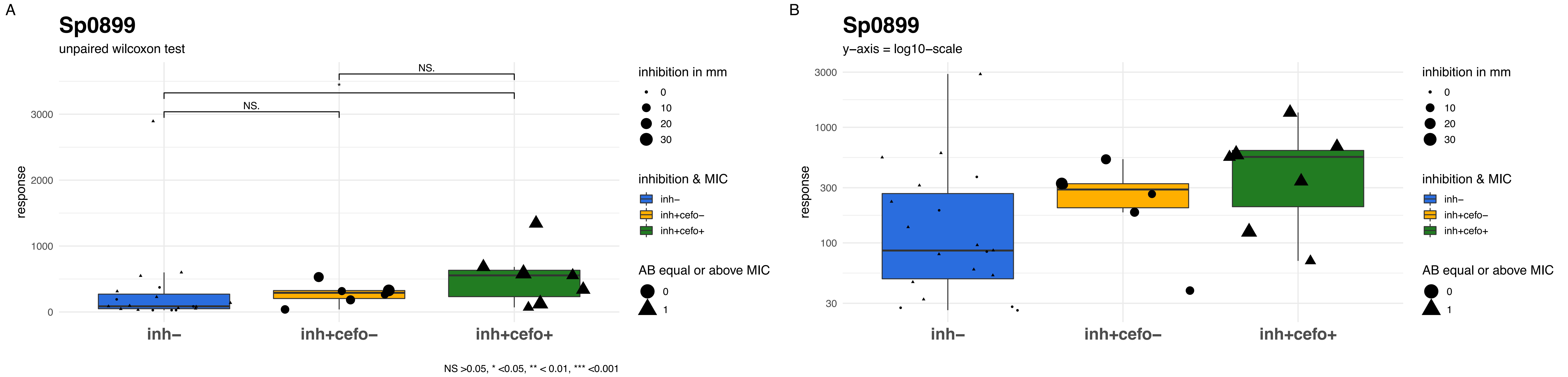
A
Sp0899
unpaired wilcoxon test
NS.
*
NS.
response
3000
2000
1000
0
inh−
inh+cefo−
inh+cefo+
inhibition in mm
0
10
20
30
inhibition & MIC
inh−
inh+cefo−
inh+cefo+
AB equal or above MIC
0
1
NS >0.05, * <0.05, ** < 0.01, *** <0.001
B
Sp0899
y−axis = log10−scale
response
3000
1000
300
100
30
inh−
inh+cefo−
inh+cefo+
inhibition in mm
0
10
20
30
inhibition & MIC
inh−
inh+cefo−
inh+cefo+
AB equal or above MIC
0
1

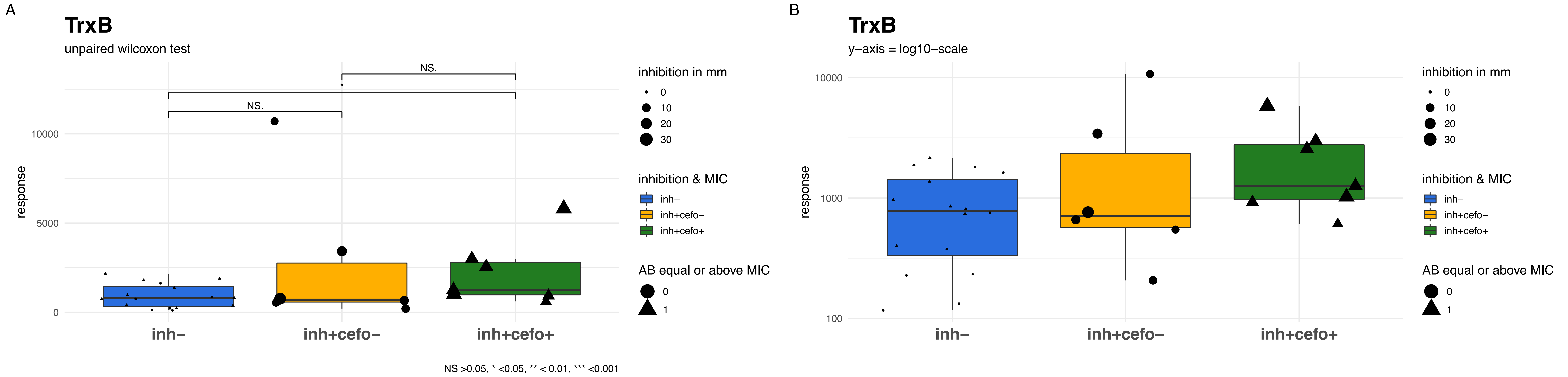

A
TrxB
unpaired wilcoxon test
NS.
*
NS.
10000
5000
0
response
inh−
inh+cefo−
inh+cefo+
inhibition in mm
0
10
20
30
inhibition & MIC
inh−
inh+cefo−
inh+cefo+
AB equal or above MIC
0
1
NS >0.05, * <0.05, ** < 0.01, *** <0.001
B
TrxB
y−axis = log10−scale
10000
1000
100
response
inh−
inh+cefo−
inh+cefo+
inhibition in mm
0
10
20
30
inhibition & MIC
inh−
inh+cefo−
inh+cefo+
AB equal or above MIC
0
1

FIG S5 Individual boxplot graphs of quantified IgG levels against the respective pneumococcal antigens. The Luminex xMAP technology and xMAPr app were used to quantify the levels of sputum IgGs specific for 55 *S. pneumoniae* antigens. The sizes of the symbols in the boxplots are proportional with the previously measured diameters of pneumococcal growth inhibition zones in mm (1). The colours of the boxes refer to the PLS-identified sputum sample groups as in Fig. 1A. The symbol shape indicates whether the quantified cefotaxime concentration was below (circle, 0), or equal/above (triangle, 1) the MIC for *S. pneumoniae* TIGR4. (A) Boxplot showing the response for a particular antigen on a non-log scale and the statistical outcome of the respective Wilcoxon rank sum test. (B) Boxplot with the response for a particular antigen on a $\log_{10}$ scale.

References

1. Seinen J, Dieperink W, Mekonnen SA, Lisotto P, Harmsen HJM, Hiemstra B, Ott A, Schultz D, Lalk M, Oswald S, Hammerschmidt S, de Smet A, van Dijl JM. 2019. Heterogeneous antimicrobial activity in broncho-alveolar aspirates from mechanically ventilated intensive care unit patients. Virulence 10:879-891.
