# Supplementary material for: Sputum Proteome Signatures of Mechanically Ventilated Intensive Care Unit Patients Distinguish Samples with or without Anti-pneumococcal Activity
Source: mSystems. 2021 Mar 2;6(2):e00702-20. doi: 10.1128/mSystems.00702-20 (PMC8546979; doi:10.1128/mSystems.00702-20)
Supplement: TABLE S1 [file msystems.00702-20-st001.docx]

**Table S1.** Overview of recombinantly expressed and purified pneumococcal antigens.

| No. | Proteins (TIGR4) | Proteins (D39) | Template DNA from *S. pneumoniae* | Primer forward (5'-3') | Primer reverse (5'-3') | Restric-tion site | Plasmid | Tag | Purifica-tion method^a-e^ | Reference |
| --- | --- | --- | --- | --- | --- | --- | --- | --- | --- | --- |
| 1 | AdcAII (SP_1002) | SPD_0888 | TIGR4(1) | GGGCGCTAGCGGTCAAAAGGAAAGTCAGAC | GCGGCCAAGCTTACTTTAATTCT  TCTGCTAG | *Nhe*I, *Hin*dIII | pTP1(2) | His_6_ | ^a^ | (3) |
| 2 | AliA (SP_0366) | SPD_0334 | D39, NCTC7466, serotype 2 | AATTGCTAGCTCTGGATCAGGTTCAAGC | GCGCGAGCTCATTTCACATGTTTTGC | *Nhe*I, *Sac*I | pTP1(2) | His_6_ | ^a^ | This work |
| 3 | AliB (SP_1527) | SPD_1357 | D39, NCTC7466, serotype 2 | CGCGTGCTAGCGGAAATTCTAGCACTGCATC | GCGCGAGCTCTTATTTGACATGTTTTGCC | *Nhe*I, *Sac*I | pTP1(2) | His_6_ | ^a^ | This work |
| 4 | AliC | - | MNZ41 | GCGCGCGCTAGCAAAAGTGAAAAGAATGC | GCGCGCGAGCTCATTTTATGTGCTTTTC | *Nhe*I, *Sac*I | pTP1(2) | His_6_ | ^a^ | This work |
| 5 | AliD | - | MNZ41 | GCGCGCACTAGTTCAGATACAAAAACTTAC | GCGCGCAAGCTTATTTAACATGTTTTTCTGC | *Spe*I, *Hin*dIII | pTP1(2) | His_6_ | ^a^ | This work |
| 6 | AmiA (SP_1891) | SPD_1671 | TIGR4(1) | GCGCGCGCTAGCAGTTCTTCAAAATCATCTGATTC | GCGCGCGAGCTCTTACTTCACATGACTTGCCAATTC | *Nhe*I, *Sac*I | pTP1(2) | His_6_ | ^a^ | This work |
| 7 | DacB (SP_0629) | SPD_0549 | TIGR4(1) | GCGCGCTAGCCAAGAAAAAACAAAAAATGAAG | GGCCAAGCTTAATCGACGTAGTCTCC | *Nhe*I, *Hin*dIII | pTP1(2) | His_6_ | ^a^ | (4) |
| 8 | Etrx1 (SP_0659) | SPD_0572 | TIGR4(1) | AAGCGCTAGCTCAGGCAAGTCCGTGACTAG | GGCCAAGCTTAGGCTAATTCCTTCAAAGTTTG | *Nhe*I, *Hin*dIII | pTP1(2) | His_6_ | ^a^ | (2) |
| 9 | Etrx2 (SP_1000) | SPD_0886 | TIGR4(1) | AAACGCTAGCGGTGAGGAAGAAACTAAAAAG | ACGCGAGCTCCTAGTTCATTTCT  TTAAATGC | *Nhe*I, *Sac*I | pTP1(2) | His_6_ | ^a^ | (2) |
| 10 | MetQ (SP_0149) | SPD_0151 | TIGR4(1) | AAAGCATATGAGCGGCGAAAACCTGTATTTTCAGGGCGCTAGCGGAAACTCAGAAAAGAAAGC | CCAACCTTCCAAGCTTACCAAAC  TGGTTGATCC | *Nde*I, *Hin*dIII | pTP1(2) | His_6_ | ^a^ | (3) |
| 11 | PccL (SP_0198) | SPD_0184 | TIGR4(1) | AAGCGCTAGCTCACAAAGAGCTCAACAGG | GGCCAAGCTTATTTAGTTAAAACGATTTGGTCCG | *Nhe*I, *Hin*dIII | pTP1(2) | His_6_ | ^a^ | This work |
| 12 | PiaA (SP_1032) | SPD_0915 | TIGR4(1) | GCGCGCGCTAGCTCTTCTAATTCTGTTAAAAATGAAG | GCGCGCGAGCTCTTATTTCGCATTTTTGCATGCAT | *Nhe*I, *Sac*I | pTP1(2) | His_6_ | ^a^ | This work |
| 13 | PnrA (SP_0845) | SPD_0739 | TIGR4(1) | AAGCGCTAGCGGTAACCGCTCTTCTCGTA | GGGGCCAAGCTTATTTTTCAGGAACTTTTACGC | *Nhe*I, *Hin*dIII | pTP1(2) | His_6_ | ^a^ | (3) |
| 14 | PpmA (SP_0981) | SPD_0868 | TIGR4(1) | CCATGGCTAGCCACCATCACCATCACCATTCGAAAGGGTCAGAAGGTGC | TCATGGATCCGGACTATTCGTTT  GATGTAC | *Nhe*I, *Bam*HI | pET11a | His_6_ | ^a^ | (5) |
| 15 | PsaA (SP_1650) | SPD_1463 | TIGR4(1) | GCGCGCTAGCGGAAAAAAAGATAC | GCGCAAGCTTATTTTGCCAATCCTTCAG | *Nhe*I, *Hin*dIII | pTP1(2) | His_6_ | ^a^ | (3) |
| 16 | SlrA (SP_0771) | SPD_0672 | TIGR4(1) | TTTACTGCATATGCACCATCAC  CATCACCATAGCAGCGTCCAACGCAGT | CATTAGGATCCAATCGCTGGGGAAGTG | *Nde*I, *Bam*HI | pET11a | His_6_ | ^a^ | (5) |
| 17 | SP_0148 | SPD_0150 | TIGR4(1) | GGCCACTAGTGGGGGTGCTAAGAAAGAA | GGCCAAGCTTATTTAATATCAGCTTCTGCCGG | *Spe*I, *Hin*dIII | pTP1(2) | His_6_ | ^a^ | This work |
| 18 | SP_0191 | SPD_0179 | TIGR4(1) | TATTTTCAGGGCGCTAGCGGACAGAAAAAAGAAACTGG | CCAACCTTCCAAGCTTATTGTTC  TGTCGCGCCATTTG | *Nhe*I, *Hin*dIII | pTP1(2) | His_6_ | ^a^ | (3) |
| 19 | SP_0899 | SPD_0792 | TIGR4(1) | GCGCGCTAGCCAACAACAACATGCTACTTC | GGCCGAGCTCTTAAAGTTTAACCCACTTATC | *Nhe*I, *Hin*dIII | pTP1(2) | His_6_ | ^a^ | (3) |
| 20 | CbpC (SP_0377) | SPD_0345 | TIGR4(1) | GCGCGCGCTAGCAATACCACAGGTGGCCGATTTG | GCGCGCAAGCTTAAATCCACTCACCAGATGAGGCG | *Nhe*I, *Hin*dIII | pTP1(2) | His_6_ | ^b^ | This work |
| 21 | CbpE (SP_0930) | SPD_0821 | TIGR4(1) | CCGAATTCAAGGAGATTAACATATGCAAGAAAGTTCAGGAAATAAAATCC | CGGGATCCTCATTATGTAGTTTT  AATTGTAGCAGATTTCTC | *Nde*I, *Bam*HI | pT7-7 | His_6_ | ^b^ | (6) |
| 22 | CbpF (SP_0391) | SPD_0357 | TIGR4(1) | GCGCGCACTAGTGATGATTCTGAAGGATGGCAG | GCGCGCAAGCTTACTTAACCCATTCACCATTATAGTTT | *Spe*I, *Hin*dIII | pTP1(2) | His_6_ | ^b^ | This work |
| 23 | CbpL (SP_0667) | SPD_0579 | TIGR4(1) | GCGCGCTAGCGAAGAAAACATCCATTTTTC | GGCCGAGCTCTTAATCATCTAAATGATCAATGG | *Nhe*I, *Sac*I | pTP1(2) | His_6_ | ^c^ | (7) |
| 24 | Chimeric (PspC+PspA) | - | NCTC 7465, serotype 1 + R 36A, ATCC11733, serotype 2 | GCGCGCGCGCGCGGATCCAAACCGGAAGCTTCAGATACAGCG(*pspC*),  CCATCTACTCCAAAAGATGCTGAAGAAGTCGCT (*pspA*) | CTTCTTCAGCATCTTTTGGAGTA  GATGGTGGTGCTGG (*pspC*), GCGCGCGCGCGCAAGCTTCTCATTAACTGCTTTCTTAAGGTC (*pspA*) | *Bam*HI, *Hin*dIII | pQE30 | His_6_ | ^a^ | This work |
| 25 | LytA (SP_1937) | SPD_1737 | D39, NCTC7466, serotype 2 | GCGCGCTAGCGAAATTAATGTGAGTAAATTAAGAACAG | GCGCAAGCTTATTTTACTGTAAT  CAAGCCATCTGGC | *Nhe*I, *Hin*dIII | pTP1(2) | His_6_ | ^b^ | This work |
| 26 | LytB (SP_0965) | SPD_0853 | TIGR4(1) | GCGCGCGCTAGCGGCCTGCATTTTGAT | GCGCGCGAGCTCTTAATCTTTGCCACCTAGCTTCTC | *Nhe*I, *Sac*I | pTP1(2) | His_6_ | ^b^ | This work |
| 27 | LytC (SP_1573) | SPD_1403 | TIGR4(1) | GCGCGCGCTAGCGCAAATGAAACTGAAGTAGC | GCGCGCAAGCTTAATACCAAACGCTGACATCTAC | *Nhe*I, *Hin*dIII | pTP1(2) | His_6_ | ^b^ | This work |
| 28 | PcpA (SP_2136) | SPD_1965 | TIGR4(1) | GGCCGCTAGCCCTAGTTCGGAAGTAATC | GCGCGCGAGCTCTTATCCTACCCACTCACCGTTAG | *Nhe*I, *Sac*I | pTP1(2) | His_6_ | ^b^ | This work |
| 29 | PspA (SP_0117)  QP2 | SPD_0126 | D39, NCTC7466, serotype 2 | GGATCCGAAGAAGAATCTCCCGTAGCC | AAGCTTATTAACTGCTTTCTTAA  GGTC | *Bam*HI, *Hin*dIII | pQE30 | His_6_ | ^a^ | (8) |
| 30 | PspC (SP_2190)  SH2 | SPD_2017 | NCTC 7465, serotype 1 | GCGCGCGCGCGCGGATCCACAGAGAACGAGGGAAGTACCC | AAGCTTTTCTTTAACTTTATCTT  CTTCTGCTG | *Bam*HI, *Hin*dIII | pQE30 | His_6_ | ^a^ | (9) |
| 31 | Hic 2  (PspC-like) | - | A66, NCTC7978, serotype 3 | GCGCGCTAGCACAGAGAAGGAGGTAACTACCC | GCGCAAGCTTATTTAGTGGAGGAGCCTGAATTCG | *Nhe*I, *Hin*dIII | pTP1(2) | His_6_ | ^a^ | (10) |
| 32 | PavB (SP_0082) SSURE 2+3 | SPD_0080 | TIGR4(1) | GGATCCAAAGACAGTATCGATGTTCCAGC | CTGCAGGTTTATGTTAATAGTGACTTTTTTAG- | *Bam*HI, *Pst*I | pQE30 | His_6_ | ^a^ | (11) |
| 33 | PfbA (SP_1833) | SPD_1617 | TIGR4(1) | GCGCGCGCTAGCGATGAAGTTGTTACTAGTTCTTC | GCGCAAGCTTATTTTTGTTTTAC  ATCTAC | *Nhe*I, *Hin*dIII | pTP1(2) | His_6_ | ^a^ | This work |
| 34 | PitB (spt_1059) | - | TIGR4(1) | GCGCGCGGATCCGATAATTCAGCAATAACCAAAG | GCGCGCCTGCAGGTCGTCGATTTTGTTAGTAAC | *Bam*HI, *Pst*I | pQE30 | His_6_ | ^a^ | This work |
| 35 | PrtA2 (SP_0641) | SPD_0558 | TIGR4(1) | CCAGGCTAGCTCACCTAGACAACAGGGAGCA | GAGTGAGCTCTCAGTGGTTCCAATTCCCAGCAA | *Nhe*I, *Sac*I | pTP1(2) | His_6_ | ^a^ | This work |
| 36 | PsrP (SP_1772) | SPD_0342 | TIGR4(1) | GCGCGCTAGCTCTGGCAATACGATT | GCGCAAGCTTACTGTGAAAGTGACTGACT | *Nhe*I, *Hin*dIII | pTP1(2) | His_6_ | ^a^ | This work |
| 37 | RrgA (SP_0462) | - | TIGR4(1) | GCGCGCTAGCGAAACGCCTGAAACCAGT | GCGCGAGCTCTTATTCTCTCTTT  GGAGGAATAG | *Nhe*I, *Sac*I | pTP1(2) | His_6_ | ^d^ | This work |
| 38 | RrgB (SP_0463) | - | TIGR4(1) | GCGCGCGGATCCGAAACGCCTGAAACCAGTC | GCGCGCGCATGCTTCTCTCTTTG  GAGGAATAGG | *Bam*HI, *Sph*I | pQE30 | His_6_ | ^a^ | This work |
| 39 | RrgC (SP_0464) | - | TIGR4(1) | GCGCGCGGATCCCAAGAAGATCACACGTTGG | GCGCGCCTGCAGATCAATCCGTGGTCGCTTG | *Bam*HI, *Pst*I | pQE30 | His_6_ | ^a^ | This work |
| 40 | SP_1992 | SPD_1789 | TIGR4(1) | GGCCGCTAGCACGATTCTAGGAAAAGATACAG | GGCCGAGCTCTAATTGTTTGCCAGCAGG | *Nhe*I, *Sac*I | pTP1(2) | His_6_ | ^a^ | This work |
| 41 | GpsB (SP_0372) | SPD_0339 | TIGR4(1) | GCGCGCGCTAGCGCAAGTATTATTTTTTCAGC | GCGCGCGAGCTCTTAAAAATCTGAGTTATCTAAAATTTG | *Nhe*I, *Sac*I | pTP1(2) | His_6_ | ^a^ | This work |
| 42 | PhpP (SP_1733) | SPD_ 1543 | TIGR4(1) | GGCCGCTAGCGAAATTTCATTATTAACAG | GCGCGCGAGCTCTTATTCTGCATCCTCCTCGTTCATAG | *Nhe*I, *Sac*I | pTP1(2) | His_6_ | ^a^ | This work |
| 43 | Pneumolysin (SP_1923) | SPD_1726 | TIGR4(1) | CGGGATCCGCAAATAAAGCAGTAAATGAC | GCGGTACCCTAGTCATTTTCTAC  CTGAG | *Bam*HI, *Kpn*I | pASK-IBA5 | Strep | ^e^ | This work |
| 44 | TrxB (SP_1458) | SPD_1287 | TIGR4(1) | GCGCGCGCTAGCTACGATACTATTATTATCGGTG | GCGCGCAAGCTTAACTATGTTCTGTAATGA | *Nhe*I, *Hin*dIII | pTP1(2) | His_6_ | ^a^ | This work |
| 45 | DacA (SP_0872) | SPD_0767 | TIGR4(1) | GCGCGCTAGCCAAGATTTTACCATTGCCGC | GGCCGAGCTCTATTTTTCAATTT  TCTTGTCTGCTACC | *Nhe*I, *Sac*I | pTP1(2) | His_6_ | ^a^ | (4) |
| 46 | MsrAB2 (SP_0573) | SPD_0660 | TIGR4(1) | ATATGCTAGCGGGCAGACAGATGCCTCG | CCCGGGCCGAGCTCTTAATCAACATAATCTAG | *Nhe*I, *Sac*I | pTP1(2) | His_6_ | ^a^ | (2) |
| 47 | PcsB (SP_2216) | SPD_2043 | RH1 | GAAACGACTGATGACAAAATTG | GATCGAATTCTTAATCTGCATAAATATATGTAAC | *Eco*RI | pRSET A | CHiC * | ^a^ | (12) |
| 48 | Enolase (SP_1128) | SPD_1012 | TIGR4(1) | GGATCCTTGTCAATTATTACTG  ATGTTTACGC | AAGCTTTTATTTTTTAAGGTTGT  AGAATGATTTC | *Bam*HI, *Hin*dIII | pQE30 | His_6_ | ^a^ | (13) |
| 49 | PGK (SP_0499) | SPD_0445 | D39, NCTC7466, serotype 2 | GGGGGATCCTTGGCAAAACTTACTGTTAAAGAG | CCCGTCGACTTATTTTTCTGTCA  AGGCTGCAAG | *Bam*HI, *Sal*I | pQE30 | His_6_ | ^a^ | (14) |
| 50 | PhtD (SP_1003) | SPD_0889 | TIGR4(1) | GCGCGCACTAGTGGTCAGGTTAAGAAAGAGTCTAATC | GCGCGCAAGCTTACTGTATAGGAGCCGGTTGAC | *Spe*I, *Hin*dIII | pTP1(2) | His_6_ | ^d^ | This work |
| 51 | SP_1069 | SPD_0954 | TIGR4(1) | GCGCGCGCTAGCCAGAATAATAAGGATGAGAAGAAAATAAC | GCGCGCAAGCTTATTCGATGACTTGTCCTGCTTC | *Nhe*I, *Hin*dIII | pTP1(2) | His_6_ | ^a^ | This work |
| 52 | NanA (SP_1326) | SPD_1499 | TIGR4(1) | GCGCGCGCTAGCCAGGAGACTGAAACTTCTGAAG | GCGCGCAAGCTTAGATCTTCAATCTTAAATG | *Nhe*I, *Hin*dIII | pTP1(2) | His_6_ | ^a^ | This work |
| 53 | PepO (SP_1647) | SPD_1460 | TIGR4(1) | GCGCGCACTAGTACACGTTATCAAGATGA | GCGCGCAAGCTTACCAAATAATCACGCGCTCCTC | *Spe*I, *Hin*dIII | pTP1(2) | His_6_ | ^a^ | This work |
| 54 | SP_0107 | SPD_0104 | TIGR4(1) | GCGCGCGCTAGCCAAGAATCATCAACTTAC | GCGCGCAAGCTTAATACCAGCCATTGTTAAGCCA | *Nhe*I, *Hin*dIII | pTP1(2) | His_6_ | ^a^ | This work |
| 55 | SP_2063 | SPD_1874 | TIGR4(1) | GCGCGCGCTAGCGAAGAAGTTCTTTGGACTGCAC | GCGCGCAAGCTTATCCATTCATTGAAACGTGAAC | *Nhe*I, *Hin*dIII | pTP1(2) | His_6_ | ^a^ | This work |

References:

1. Tettelin H, Nelson KE, Paulsen IT, Eisen JA, Read TD, Peterson S, Heidelberg J, DeBoy RT, Haft DH, Dodson RJ, Durkin AS, Gwinn M, Kolonay JF, Nelson WC, Peterson JD, Umayam LA, White O, Salzberg SL, Lewis MR, Radune D, Holtzapple E, Khouri H, Wolf AM, Utterback TR, Hansen CL, McDonald LA, Feldblyum TV, Angiuoli S, Dickinson T, Hickey EK, Holt IE, Loftus BJ, Yang F, Smith HO, Venter JC, Dougherty BA, Morrison DA, Hollingshead SK, Fraser CM. 2001. Complete genome sequence of a virulent isolate of Streptococcus pneumoniae. Science 293:498-506.

2. Saleh M, Bartual SG, Abdullah MR, Jensch I, Asmat TM, Petruschka L, Pribyl T, Gellert M, Lillig CH, Antelmann H, Hermoso JA, Hammerschmidt S. 2013. Molecular architecture of Streptococcus pneumoniae surface thioredoxin-fold lipoproteins crucial for extracellular oxidative stress resistance and maintenance of virulence. EMBO Mol Med 5:1852-70.

3. Voss F, Kohler TP, Meyer T, Abdullah MR, van Opzeeland FJ, Saleh M, Michalik S, van Selm S, Schmidt F, de Jonge MI, Hammerschmidt S. 2018. Intranasal Vaccination With Lipoproteins Confers Protection Against Pneumococcal Colonisation. Front Immunol 9:2405.

4. Abdullah MR, Gutierrez-Fernandez J, Pribyl T, Gisch N, Saleh M, Rohde M, Petruschka L, Burchhardt G, Schwudke D, Hermoso JA, Hammerschmidt S. 2014. Structure of the pneumococcal l,d-carboxypeptidase DacB and pathophysiological effects of disabled cell wall hydrolases DacA and DacB. Mol Microbiol 93:1183-206.

5. Hermans PW, Adrian PV, Albert C, Estevao S, Hoogenboezem T, Luijendijk IH, Kamphausen T, Hammerschmidt S. 2006. The streptococcal lipoprotein rotamase A (SlrA) is a functional peptidyl-prolyl isomerase involved in pneumococcal colonization. J Biol Chem 281:968-76.

6. Lagartera L, Gonzalez A, Stelter M, Garcia P, Kahn R, Menendez M, Hermoso JA. 2005. Crystallization and preliminary X-ray diffraction studies of the pneumococcal teichoic acid phosphorylcholine esterase Pce. Acta Crystallogr Sect F Struct Biol Cryst Commun 61:221-4.

7. Gutierrez-Fernandez J, Saleh M, Alcorlo M, Gomez-Mejia A, Pantoja-Uceda D, Trevino MA, Voss F, Abdullah MR, Galan-Bartual S, Seinen J, Sanchez-Murcia PA, Gago F, Bruix M, Hammerschmidt S, Hermoso JA. 2016. Modular Architecture and Unique Teichoic Acid Recognition Features of Choline-Binding Protein L (CbpL) Contributing to Pneumococcal Pathogenesis. Sci Rep 6:38094.

8. Hammerschmidt S, Bethe G, Remane PH, Chhatwal GS. 1999. Identification of pneumococcal surface protein A as a lactoferrin-binding protein of Streptococcus pneumoniae. Infect Immun 67:1683-7.

9. Hammerschmidt S, Tillig MP, Wolff S, Vaerman JP, Chhatwal GS. 2000. Species-specific binding of human secretory component to SpsA protein of Streptococcus pneumoniae via a hexapeptide motif. Mol Microbiol 36:726-36.

10. Kohler S, Hallstrom T, Singh B, Riesbeck K, Sparta G, Zipfel PF, Hammerschmidt S. 2015. Binding of vitronectin and Factor H to Hic contributes to immune evasion of Streptococcus pneumoniae serotype 3. Thromb Haemost 113:125-42.

11. Jensch I, Gamez G, Rothe M, Ebert S, Fulde M, Somplatzki D, Bergmann S, Petruschka L, Rohde M, Nau R, Hammerschmidt S. 2010. PavB is a surface-exposed adhesin of Streptococcus pneumoniae contributing to nasopharyngeal colonization and airways infections. Mol Microbiol 77:22-43.

12. Stamsas GA, Havarstein LS, Straume D. 2013. CHiC, a new tandem affinity tag for the protein purification toolbox. J Microbiol Methods 92:59-63.

13. Bergmann S, Rohde M, Chhatwal GS, Hammerschmidt S. 2001. alpha-Enolase of Streptococcus pneumoniae is a plasmin(ogen)-binding protein displayed on the bacterial cell surface. Mol Microbiol 40:1273-87.

14. Bernardo-Garcia N, Bartual SG, Fulde M, Bergmann S, Hermoso JA. 2011. Crystallization and preliminary X-ray diffraction analysis of phosphoglycerate kinase from Streptococcus pneumoniae. Acta Crystallogr Sect F Struct Biol Cryst Commun 67:1285-9.
