# Supplementary material for: Sputum Proteome Signatures of Mechanically Ventilated Intensive Care Unit Patients Distinguish Samples with or without Anti-pneumococcal Activity
Source: mSystems. 2021 Mar 2;6(2):e00702-20. doi: 10.1128/mSystems.00702-20 (PMC8546979; doi:10.1128/mSystems.00702-20)
Supplement: Supplementary file 6 [file msystems.00702-20-sf002.docx]

A

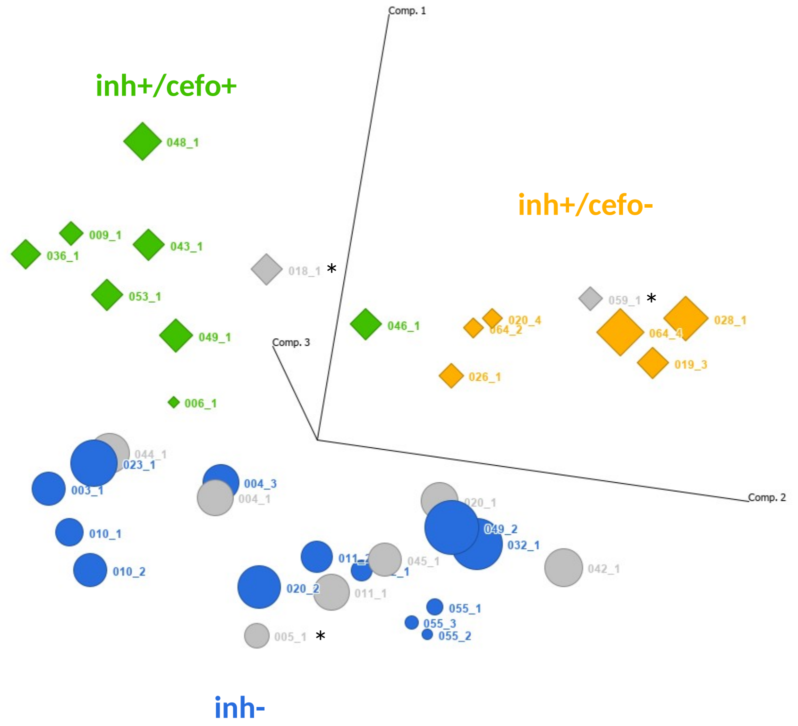

inh+/cefo+
inh+/cefo-
inh-
Comp. 1
Comp. 2
Comp. 3
048_1
009_1
036_1
043_1
018_1 *
053_1
049_1
046_1
006_1
059_1 *
020_4
064_2
064_4
028_1
019_3
026_1
044_1
023_1
003_1
004_3
004_1
010_1
010_2
020_1
049_2
032_1
042_1
045_1
020_2
011_1
055_1
055_3
055_2
005_1 *

B

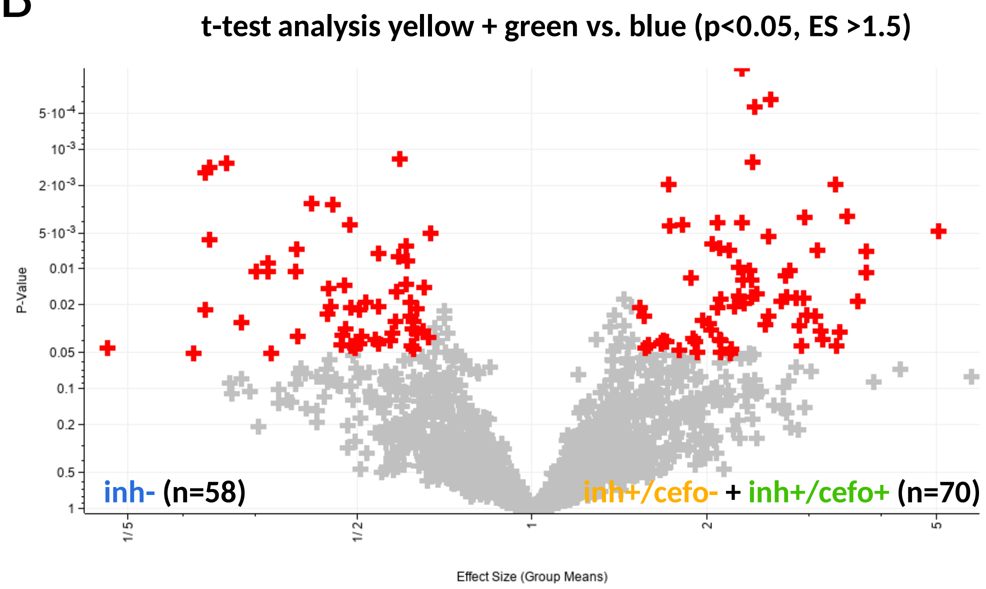

t-test analysis yellow + green vs. blue (p<0.05, ES >1.5)
P-Value
5·10⁻⁴
10⁻³
2·10⁻³
5·10⁻³
0.01
0.02
0.05
0.1
0.2
0.5
1
1/5
1/2
1
2
5
Effect Size (Group Means)
inh- (n=58)
inh+/cefo- + inh+/cefo+ (n=70)
