# Supplementary material for: Sputum Proteome Signatures of Mechanically Ventilated Intensive Care Unit Patients Distinguish Samples with or without Anti-pneumococcal Activity
Source: mSystems. 2021 Mar 2;6(2):e00702-20. doi: 10.1128/mSystems.00702-20 (PMC8546979; doi:10.1128/mSystems.00702-20)
Supplement: Supplementary file 7 [file msystems.00702-20-sf004.docx]

** **	* *	** *	*	** **
** *	** **	** **	** **	** *
** **	** *	*	** **	** **
			** **	
